# Morphology heterogeneity within a *Campylobacter jejuni* helical population: the use of calcofluor white to generate rod‐shaped *C. jejuni* 81‐176 clones and the genetic determinants responsible for differences in morphology within 11168 strains

**DOI:** 10.1111/mmi.13672

**Published:** 2017-04-24

**Authors:** Emilisa Frirdich, Jacob Biboy, Steven Huynh, Craig T. Parker, Waldemar Vollmer, Erin C. Gaynor

**Affiliations:** ^1^ Department of Microbiology and Immunology University of British Columbia Vancouver BC Canada V6T 1Z3; ^2^ Institute for Cell and Molecular Biosciences, The Centre for Bacterial Cell Biology Newcastle University Newcastle Upon Tyne NE2 4AX UK; ^3^ Agricultural Research Service, U.S. Department of Agriculture Produce Safety and Microbiology Research Unit Albany CA 94710 USA

## Abstract

*Campylobacter jejuni* helical shape is important for colonization and host interactions with straight mutants having altered biological properties. Passage on calcofluor white (CFW) resulted in *C. jejuni* 81‐176 isolates with morphology changes: either a straight morphology from frameshift mutations and single nucleotide polymorphisms in peptidoglycan hydrolase genes *pgp1* or *pgp2* or a reduction in curvature due a frameshift mutation in *cjj81176_1105*, a putative peptidoglycan endopeptidase. Shape defects were restored by complementation. Whole genome sequencing of CFW‐passaged strains showed no specific changes correlating to CFW exposure. The *cjj81176_1279* (*recR*; recombinational DNA repair) and *cjj81176_1449* (unknown function) genes were highly variable in all 81‐176 strains sequenced. A frameshift mutation in *pgp1* of our laboratory isolate of the straight genome sequenced variant of 11168 (11168‐GS) was also identified. The PG muropeptide profile of 11168‐GS was identical to that of Δ*pgp1* in the original minimally passaged 11168 strain (11168‐O). Introduction of wild type *pgp1* into 11168‐GS did not restore helical morphology. The *recR* gene was also highly variable in 11168 strains. Microbial cell‐to‐cell heterogeneity is proposed as a mechanism of ensuring bacterial survival in sub‐optimal conditions. In certain environments, changes in *C. jejuni* morphology due to genetic heterogeneity may promote *C. jejuni* survival.

## Introduction

*Campylobacter jejuni* is one of the most common causes of human bacterial diarrheal diseases worldwide (Epps *et al*., 2013). It is a Gram‐negative microaerophilic organism requiring rich media for growth *in vitro*. Despite its metabolic limitations, it can successfully compete with the human intestinal microflora with ingestion of as few as 500 bacteria resulting in human disease (Black *et al*., 1988). Unlike in humans, *C. jejuni* colonizes the gastrointestinal tract of birds and many animal species without causing disease (Epps *et al*., 2013). This has a direct effect on human health with contaminated meat (primarily poultry and poultry products), unpasteurized milk and water serving as the primary sources of human infection (Epps *et al*., 2013).

*Campylobacter jejuni* has a helical morphology that, along with its polar flagella, is responsible for the characteristic corkscrew motility hypothesized to confer an advantage over rod‐shaped bacteria in moving through viscous solutions, such as the mucus layer of the gastrointestinal tract (Lertsethtakarn *et al*., 2011).

Bacterial morphology is maintained by the peptidoglycan (PG) layer. The PG of Gram‐negative bacteria is composed of strands of alternating repeat units of β1‐4 linked *N*‐acetylglucosamine (GlcNAc) and *N*‐acetylmuramic acid (MurNAc) cross‐linked by pentapeptide side chains on the MurNAc residues. PG hydrolases cleaving the glycan backbone and peptide sidechains are important in the PG remodeling required to generate a particular morphology (Vollmer *et al*., 2008; Typas *et al*., 2012; Frirdich and Gaynor, 2013). As in *Helicobacter pylori*, *C. jejuni* homologs of the *H. pylori* CcmA, Csd1 endopeptidase, and Csd3/HdpA endo/carboxypeptidase affect the amount of curvature of the helical cell (E. Frirdich and E.C. Gaynor, unpublished). PG peptidases Pgp1 and Pgp2 are the only enzymes identified so far that result in a straight rod‐shaped morphology when deleted (Frirdich *et al*., 2012; 2014). Pgp1 has dl‐carboxypeptidase activity cleaving PG tripeptides to dipeptides (Frirdich *et al*., 2012) and Pgp2 is an ld‐carboxypeptidase cleaving PG tetrapeptides to tripeptides, providing the substrate for Pgp1 (Frirdich *et al*., 2014). The change in shape and PG structure resulting from *pgp1* and *pgp2* deletions altered the biological properties of *C. jejuni* and how it interacts with its environment: its ability to be transmitted between hosts was compromised (displaying reduced motility and biofilm formation), it was defective in colonization in a chick model, and it showed altered activation levels of host cell receptors that differed depending on the mutant strain. *C. jejuni* Δ*pgp1* PG hyperactivated the cytoplasmic nucleotide‐binding oligomerization domain 1 (Nod1) receptor in comparison with wild type and the Δ*pgp1* mutant increases secretion of the IL‐8 chemokine by epithelial cells, while Δ*pgp2* PG decreased Nod1 stimulation. The Δ*pgp2* mutant had no effect on IL‐8 secretion levels (Frirdich *et al*., 2012; 2014).

There have been reports in the literature of straight *C. jejuni* strains arising from helical strains, although the basis of the altered morphology in these cases has yet to be established. Passage of an avirulent *C. jejuni* strain through chick embryos led to the isolation of more virulent rod‐shaped strains better able to resist phagocytosis and survive *in vivo* (Field *et al*., 1991; 1993). In another study, various *C. jejuni* strains were used to colonize chickens and the strains were reexamined after passage through the chicken gut. The chicken isolates of a strongly colonizing helical bovine strain 305/94 were rod‐shaped (Hanel *et al*., 2009). Some flagellar mutants have been described as having a straight morphology (Wassenaar *et al*., 1991; Matz *et al*., 2002; Fernando *et al*., 2007), although in other instances the same mutation has also resulted in no morphology changes (Hendrixson and DiRita, 2003, Joslin and Hendrixson, 2009; E. Frirdich and E.C. Gaynor, unpublished), indicating that the morphology changes were unlinked to the flagellar mutants and occurring spontaneously. Spontaneous straight variants arising after laboratory passage of *C. jejuni* strain 11168 and *Campylobacter coli* have also been reported (Gaynor *et al*., 2004, Ziprin *et al*., 2005). The complete genome sequence of a rod‐shaped *C. jejuni* variant revealed a nucleotide deletion and subsequent truncation of the *pgp1* gene product (Gunther IV *et al*., 2015) important in helical shape determination (Frirdich *et al*., 2012), as described below. The presence of these numerous straight mutants would suggest that genes involved in the determination or regulation of *Campylobacter* helical shape may be undergoing phase variation or accumulating nonsense or missense mutations resulting in non‐functional proteins. Selection of these mutants indicates that a rod‐shaped morphology accompanied with the underlying PG changes may provide an advantage to *C. jejuni* survival under certain growth conditions.

The *pgp1* gene was identified in a transposon (Tn) mutant library screen on calcofluor white (CFW) for hypofluorescent or *dim* mutants (Frirdich *et al*., 2012). CFW binds β1‐3 and β1‐4 carbohydrate linkages such as those found in several bacterial cell surface carbohydrates and fluoresces under long wave UV light (Rattee and Breur, 1974, Wood, 1980). Despite the unknown nature of the exact *C. jejuni* surface carbohydrate contributing to CFW hypo‐ and hyperfluorescence, mutants displaying altered reactivity to CFW have been found to also display changes in stress survival and pathogenesis phenotypes (McLennan *et al*., 2008; Naito *et al*., 2010; Frirdich *et al*., 2012; E. Frirdich and E.C. Gaynor, unpublished). In the Tn mutant library screen on CFW that identified *pgp1* as having a role in maintaining helical cell shape, other Tn mutants were identified with altered cell morphology. However, unlike with *pgp1*, the cell shape of these mutants was unlinked to the Tn insertion. This study examined the genetic basis for these shape changes. Of the 8 mutants, 6 had frameshift mutations resulting in non‐functional copies of *pgp1*, and two had mutations in *cjj81176_1105* [the homolog of the *H. pylori csd1* gene encoding a PG peptidase involved in determining cell curvature in that organism (Sycuro *et al*., 2010)]. These were likely generated by passage on CFW, as passage of the wild type 81‐176 strain on CFW and not on media lacking CFW, also resulted in rod‐shape variants from mutations in *pgp1* or *pgp2*. The genetic variation arising from the passage of *C. jejuni* 81‐176 on CFW was examined by whole genome sequencing (WGS), as was that of *C. jejuni* 11168‐GS (genome sequenced) that has a rod‐shaped morphology and is the laboratory passaged variant of the 11168‐O (original) helical strain (Gaynor *et al*., 2004). The rod‐shaped morphology of 11168‐GS was also found to be attributed to a frameshift mutation in 11168 *pgp1*. Our results show that due to mutations in *pgp1* and *pgp2* and their effect on PG structure, *C. jejuni* can alter its shape to a straight rod. These changes in shape would also alter the biological properties of the organism and may be advantageous to an aspect of its lifecycle either in environmental survival, transmission, colonization or infection that remains to be determined.

## Results

### Straight Tn mutants isolated due to their hypofluorescent phenotype on CFW resulted from mutations in *pgp1* and CFW hypofluorescent Tn mutants with decreased curvature from mutations in *cjj81176_1105*

**Figure 1:**
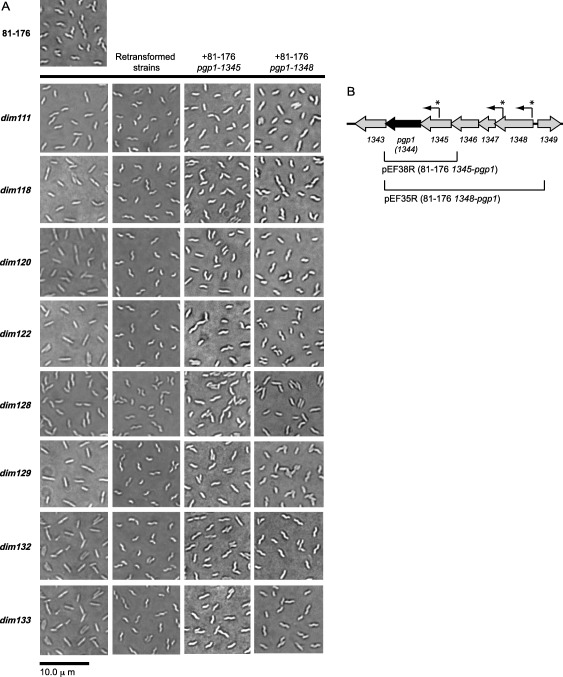
*Campylobacter jejuni* 81‐176 Tn mutants isolated from a screen on CFW for their *dim* phenotype exhibited a change in morphology unlinked to the Tn insertion. A. DIC microscope images of *C. jejuni* wild type 81‐176 and *dim* mutants grown on solid media for 18 h. Column I shows DIC images of the wild type 81‐176 strain and the *dim* mutants isolated from a screen on CFW which exhibited a loss in cell curvature and straightened cell morphology that were not previously reported. The rod‐shaped *dim100* mutant having an insertion in the *cjj81176_1344* gene (renamed *pgp1*) was previously published (Frirdich *et al*., 2012). The DIC images depicted in column II represent the morphology of the wild type strain 81‐176 strain transformed with genomic DNA from the respective *dim* mutants and selected for by the Km antibiotic resistance marker carried by the Tn to remake the mutations. These strains did not retain the changes in morphology seen after the mutants were isolated on CFW, indicating that the original changes in morphology were unlinked to the Tn insertion, which was not the case with *dim100* (Frirdich *et al*., 2012). The DIC images in columns III and IV are those of the original *dim* mutants expressing *pgp1* and varying amounts of the upstream region of the *pgp1* gene cluster in the rRNA spacer region of the chromosome. The plasmids used for this complementation analysis are shown in panel B. B. the *C. jejuni* 81‐176 *pgp1* gene locus. The regions cloned into the integrative vector pRRC (Cm^R^) to form plasmids pEF35R and pEF38R used for complementation analysis (Panel A, columns III and IV) are shown below the gene cluster. An R after the plasmid name indicates that the region is cloned in the opposite direction as the antibiotic resistance cassette promoter. Transcriptional start sites as predicted by *C. jejuni* transcriptome analysis carried out by Dugar *et al*. (2013) are designated by an asterisk and an arrow indicating the direction of transcription.

A mariner Tn mutant library was screened on CFW for hypofluorescent or *dim* mutants as part of a previous study (Frirdich *et al*., 2012). Of the approximately 400 *dim* mutants isolated, 8 mapped to distinct regions of the *cjj81176_1344* gene which was renamed *pgp1* (peptidoglycan peptidase 1) for its role as a PG dl‐carboxypeptidase (Frirdich *et al*., 2012). Deletion of *pgp1* resulted in a straight morphology and loss of the characteristic *C. jejuni* helical cell shape (Frirdich *et al*., 2012). The role of Pgp1 in *C. jejuni* PG biosynthesis and its effect on *C. jejuni* biology were described as the basis of a previous study (Frirdich *et al*., 2012). In addition to the mutants in *pgp1*, several other Tn mutants displayed an alteration in *C. jejuni* cell morphology resulting in cell straightening (Fig. 1A column I, Table 1): some were straight rods (*dim118*, *dim120*, *dim122*, *dim129*, *dim132*, and *dim133*) and others were still helical, but displayed a decreased amount of curvature (*dim111* and *dim128*). The location of the Tn insertions were mapped as described previously (Frirdich *et al*., 2012), with no obvious candidates in genes predicted to affect cell morphology (Table 1).

**Table 1:** Summary of the nucleotide changes in the *pgp1* gene of the *dim* mutants isolated from a Tn mutant library screen on CFW and cellular morphology of the mutants, retransformed mutants, and original mutants expressing *pgp1* and varying amounts of the upstream region of *pgp1* (as the promoter of *pgp1* is unknown) displayed in Fig. 1.

	Location of Tn insertion	Description of mutation in *pgp1* (1395 bp)^a^	Sequence surrounding mutation	Predicted length of Pgp1 (amino acids)^b^	Shape^c^	Shape of retransformed mutant^d^	Shape of mutant expressing *pgp1‐1345* at the rRNA locus^e^	Shape of mutant expressing *pgp1‐1348* at the rRNA locus^f^
*dim111, dim128* ^g^	Cjj81176_0478/ThiC Thiamine biosynthesis protein	Wild type *pgp1*	Not applicable	464	Very slightly helical	Helical	Very slightly helical	Very slightly helical
*dim118*	Cjj81176_ pTet0039/cmbg_11 VirB11‐like ATPase	A deleted at NT 1187	AAA‐AAAA (A tract)	402	Straight	Helical	Slightly curved	Helical
*dim120*	Cjj81176_pVir0003 VirB10 homolog (similar to VirB)	G inserted after NT 386	GGGGG (G tract)	131	Straight	Helical	Slightly curved	Helical
*dim122*	Cjj81176_0307 Transaldolase	G deleted at NT 412	GAT‐CGATGC (GATGC repeat)	149	Straight	Helical	Slightly curved	Helical
*dim129*	Cjj81176_0315 PEB3	A deleted at NT 1187	AAA‐AAAA (A tract)	402	Straight	Helical	Slightly curved	Helical, few straight
*dim132*	Cjj81176_0206/7 Hypothetical protein	A inserted after NT 1077	AAAA (A tract)	372	Straight	Helical	Helical, few straight	Helical, few straight
*dim133*	Cjj81176_0307 Transaldolase	G deleted at NT 412	GAT‐CGATGC (GATGC repeat)	149	Straight	Helical	Slightly curved	Slightly curved

**a.** The results of *pgp1* sequencing. The sequence of *pgp2* was identical to that of wild type.

**b.** The full‐length Pgp1 is 464 amino acids. Frameshift mutations introduce premature STOP codons.

**c.** Summary of column I Fig. 1A.

**d.** The wild type *C. jejuni* 81‐176 strain was transformed with genomic DNA from the respective *dim* mutants and selected for by the Km antibiotic resistance marker carried by the Tn to remake the mutations in a clean background. Summary of column II Fig. 1A.

**e.** The *1344‐1345* region from pEF38R (Fig. 1B) was integrated into the rRNA spacer region. Summary of column III Fig. 1A.

**f.** The *1344‐1348* region from pEF35R (Fig. 1B) was integrated into the rRNA spacer region. Summary of column IV Fig. 1A.

**g.** The *dim111* and *dim128* mutants have a deletion in *cjj81176_1105* deleting NT 743‐746 (ACCT) resulting in a frameshift mutation and premature stop codon (Supporting Information Table S3).

### Identifying the genetic basis for hypofluorescence in *dim111, dim118, dim120, dim122, dim128, dim129, dim132, and dim133*

To determine whether the changes in cell morphology in *dim* mutants *dim111*, *dim118*, *dim120*, *dim122*, *dim128*, *dim129*, *dim132*, and *dim133* were linked to the Tn insertion, the wild type *C. jejuni* helical 81‐176 strain was transformed with genomic DNA from the *dim* mutants and plated on the antibiotic corresponding to the antibiotic resistance cassette encoded by the mariner Tn antibiotic resistance cassette to remake the mutations. Unlike with the Tn insertions in *pgp1*, the straightened morphology (Fig. 1A column II; Table 1) and the *dim* phenotype *dim111*, *dim118*, *dim120*, *dim122*, *dim128*, *dim129*, *dim132*, and *dim133* mutants was not retained in the remade strains. Since *pgp1* and *pgp2* are the only *C. jejuni* genes known to produce rod‐shaped cells when deleted, the *pgp1* and *pgp2* genes in each *dim* mutant were sequenced to determine whether changes in either of these genes was affecting the morphology of the mutants. Nucleotide insertions or deletions were detected in *pgp1* in *dim118*, *120*, *122*, *129*, *132*, and *133* (Table 1, Supporting Information Fig. S1). Four different mutations were identified: a deletion from A8 to A7 in a poly‐A tract starting at nucleotide 1184 (*dim120*), an increase from G4 to G5 in a poly‐G tract at nucleotide 383 (*dim118* and *dim129*), a deletion of a G from a GATGC repeat sequence at nucleotide 412 (*dim122* and *dim133*), and an insertion from A3 to A4 in a poly‐A tract starting at nucleotide 1077 (*dim132*). These changes resulted in frameshift mutations introducing premature stop codons and truncated versions of Pgp1 (Table 1). The nucleotide deletion in *dim118* and *dim129* was identical to that identified in the *pgp1* gene of *C. jejuni* RM1285 (Gunther IV *et al*., 2015). The *pgp2* gene sequence was identical to wild type in all mutants.

The wild type 81‐176 *pgp1* gene was introduced into the *dim* mutants to ascertain whether the changes in morphology could be restored. Complementation analysis with *pgp1* is complicated by the fact that (1) *pgp1* copy number affects complementation (increased levels of *pgp1* results in changes in morphology); and (2) the *pgp1* gene is located in the middle of a putative operon so the location of the *pgp1* promoter is unclear (Frirdich *et al*., 2012). Transcriptional start sites predicted by *C. jejuni* transcriptome analysis (Dugar *et al*., 2013) are indicated in Fig. 1B. Plasmids pEF35R and pEF38R were used for complementation analysis and contain *pgp1* with varying amounts of the upstream region (Fig. 1B) cloned into the pRRC (Cm^R^) plasmid (Karlyshev and Wren, 2005). The *pgp1* region was cloned in the reverse orientation to the *cat* ^R^ cassette to avoid overexpression from the *cat* promoter and integrated into the rRNA spacer region. The pEF38R construct expressing *pgp1* and *cjj81176_1345* is the minimal construct that complements the shape defect in a *Δpgp1* mutant (data not shown); and the plasmid pEF35R contains the entire upstream region of *pgp1* (*cjj81176_1345‐ cjj81176_1348*) and complements all the *Δpgp1* mutant phenotypes to wild type (Frirdich *et al*., 2012). Wild type morphology was partially restored in *dim118*, *dim120*, *dim122*, *dim129*, *dim132*, and *dim133* with the introduction of *pgp1‐1345* (from pEF38R) and completely restored with *pgp1‐1348* (from pEF35R) (Fig. 1A, Table 1). Complementation of the *dim118* and *dim129* morphology defect with wild type *pgp1* indicates that the rod shaped morphology of the *C. jejuni* strain described by Gunther IV *et al*. (2015) (with a similar mutation in *pgp1* as *dim118* and *dim129*) could likely also be complemented by wild type *pgp1*, although complementation analysis is required to confirm this. The morphology of *dim111* and *dim128* was unchanged with the introduction of *pgp1*, as expected, since these mutants did not have a mutated copy of *pgp1* (Fig. 1A, Table 1). WGS of *dim111* and *dim128* identified a deletion in *cjj81176_1105* deleting nucleotides 743‐746 (‐ACCT) resulting in a frameshift mutation and premature stop codon (Supporting Information Table S3). The *cjj81176_1105* gene product is a homolog of *H. pylori* Csd1 (30% identity/52% similarity). Csd1 is a PG endopeptidase involved in determination of cell curvature in *H. pylori* (Sycuro *et al*., 2010). The morphology of a mutant in *cjj81176_1105* resembled that of *dim111* and *dim128* with cell straightening, but not a complete loss of curvature (Supporting Information Fig. S2). Introduction of 1105 from pRRC‐*1105* into the rRNA locus of *dim111* and *dim128* restored the morphology defect of those mutants (Supporting Information Fig. S2).

### Selection of straight *C. jejuni* 81‐176 isolates from a helical wild type population by passage on CFW

**Figure 2:**
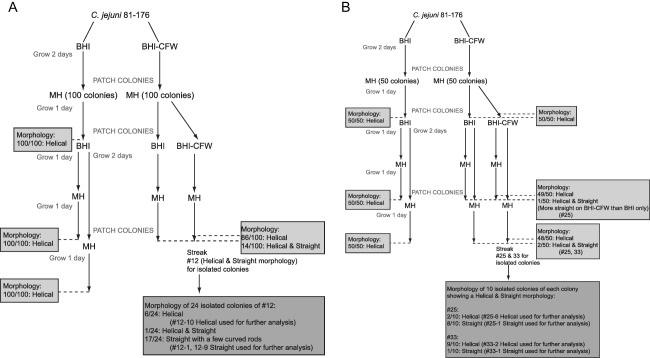
CFW screen used to select for straight *C. jejuni* 81‐176 isolates from a helical wild type population, mimicking the CFW screen used to isolate *dim* mutants from a Tn mutant library. An 18 h plate grown culture of *C. jejuni* 81‐176 was resuspended in BHI broth, standardized to an OD of 0.3 and 0.1 ml of a 10^−5^ dilution of the broth suspension of OD 0.3 was plated on BHI only (control) and BHI plates with CFW. The plates were incubated for 2 days and 100 (for screen I; A) or 50 (for screen II; B) colonies were picked from each of the BHI and BHI‐CFW plates and patched to MH. After 1 day, the colonies were re‐patched to new BHI and BHI‐CFW plates. After a subsequent 1 or 2 days, the colonies were patched to MH. Cell morphology was examined by DIC microscopy at different points in the screen from colonies patched to MH, as indicated in the figure. Numbers represent how many colonies of the total have the observed morphology. Select colonies displaying bacteria with a straight morphology were streaked for isolated colonies [colony #12 for screen I (A), and colonies #25 and 33 for screen II (B)] and the morphology of these were re‐examined. Of these, colonies selected for further analysis are described in the figure.

**Figure 3:**
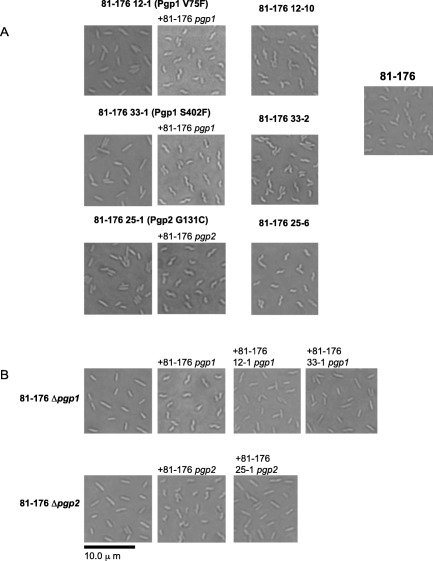
Cellular morphology of *C. jejuni* 81‐176 straight isolates from CFW passaging and complementation analysis. DIC microscope images of *C. jejuni* strains grown on solid media for 18 h. A. cellular morphology of isolates with a straight and helical phenotype from colonies #12, 25, and 33 isolated from passaging of *C. jejuni* 81‐176 on CFW (Fig. 2). The *pgp1* and *pgp2* genes were sequenced in all isolates. Nucleotide differences resulting in amino acid changes are indicated in brackets. For complementation analysis, the *pgp1* or *pgp2* genes were introduced into the rRNA spacer region in isolates in which a mutation was identified. The *C. jejuni* 81‐176 region used for *pgp1* and *pgp2* complementation was that from plasmid pEF38R indicated in Fig. 1B and pJV4 described in (Frirdich *et al*., 2014) respectively. B. complementation analysis of *pgp1* and *pgp2* mutations demonstrating the lack of function of the single amino acid changes in isolates 12‐1, 25‐1, and 33‐1. The *C. jejuni* 81‐176 *pgp1* region inserted into the rRNA spacer region was that from pEF38R (Fig. 1B) and the 12‐1 and 33‐1 *pgp1* regions were identical to the region used in pEF38R forming plasmids pEF82R and pEF80R respectively. The *C. jejuni* 81‐176 *pgp2* gene and upstream region was that of pJV4 and a similar region from 25‐1 that was cloned into pEF81.

Since a Tn mutant library screen on CFW selected for mutants with changes in cell morphology unlinked to the Tn insertion, the *C. jejuni* wild type strain 81‐176 was passaged on CFW to determine whether it was the passage on CFW that generates or selects for straight isolates. A similar protocol to the original CFW screen (Frirdich *et al*., 2012) was used and is outlined in Fig. 2 with the exception that colonies were randomly selected and not selected by fluorescence. Two screens on CFW were carried out (Fig. 2A and B). In the first screen (Fig. 2A), a total of 100 colonies were carried through the screen. The controls plated on BHI only for 1 or 2 passages were all helical. The colonies exposed to CFW were examined for shape only after a second passage either on BHI‐CFW or on BHI alone and only after 1 day of growth (Fig. 2A). Of the 100 colonies, 14 displayed a mixture of cell morphologies with both helical and straight cells after one passage on CFW, regardless of whether the second passage was on BHI‐CFW or BHI. One of these colonies (colony 12) was re‐streaked for isolated colonies on Mueller‐Hinton (MH). Of 24 of the isolated colonies from colony 12 examined for cell morphology, 6 were entirely helical, 1 was a mixture of helical and straight cells, and 17 were mainly straight with a few curved cells. From these, colonies 12‐1 and 12‐9 (straight with a few curved cells; 12‐1 is shown in Fig. 3A) and 12‐10 (helical; Fig. 3A) were selected for further analysis. For the second screen, fewer colonies (50 rather than 100), but more conditions were tested for their effect on morphology (Fig. 2B). As in the first screen, all colonies plated on BHI alone remained helical (Fig. 2B). After the first passage on BHI‐CFW, all 50 colonies were helical (Fig. 2B). After a second passage on either BHI or BHI‐CFW, colony 25 had helical and straight cells in the population after 1 day of growth (Fig. 2B), with more straight cells after growth on CFW. After 2 days of growth on BHI and CFW, straight cells appeared in the population of colony 33 (Fig. 2B). Colonies 25 and 33 were re‐streaked for isolated colonies on MH. For colony 25, of the 10 isolated colonies examined, 2 were helical and 8 were straight (Fig. 2B). Straight colony 25‐1 and helical colony 25‐6 were selected for further analysis (Fig. 3A). For colony 33, of the 10 isolated colonies examined, 9 were helical and 1 was straight (Fig. 2B). Straight colony 33‐1 and helical colony 33‐2 were selected for further analysis (Fig. 3A). Increasing the amount of CFW added to the BHI plates up to tenfold had no effect on viability and did not increase the observed number of colonies displaying a straight morphology.

### Straight *C. jejuni* 81‐176 isolates from passage on CFW result from point mutations in *pgp1* and *pgp2*

To determine whether the straight morphology of CFW‐passaged *C. jejuni* 81‐176 isolates were due to changes in *pgp1* or *pgp2*, these genes were sequenced in 12‐1, 12‐9, 12‐10, 25‐1, 25‐6, 33‐1 and 33‐2. Isolates 12‐1, 12‐9, and 33‐1 all had single nucleotide polymorphisms (SNPs) in *pgp1* (G1205T for 12‐1 and 12‐9, and C523T in 33‐1; Supporting Information Fig. S1) and 25‐1 a SNP in *pgp2* (G391T; Supporting Information Fig. S3), as confirmed by WGS (Supporting Information Table S3). These mutations result in single amino acid changes (Supporting Information Figs S1 and S3). The *pgp1* and *pgp2* sequences in helical isolates were identical to those of the wild type 81‐176 (Supporting Information Table S3). Since 12‐1 and 12‐9 had identical mutations, only 12‐1 was used for further analysis.

The wild type 81‐176 *pgp1* and *pgp2* genes were introduced into the rRNA spacer region of the corresponding SNP‐containing isolates to determine if the SNPs resulted in the shape phenotype. The *C. jejuni* 81‐176 region used for *pgp1* and *pgp2* complementation was that from plasmid pEF38R indicated in Fig. 1B and pJV4 described previously (Frirdich *et al*., 2014) respectively. Expression of wild type *pgp1* in isolates 12‐1 and 33‐1 and wild type *pgp2* in isolate 25‐1 restored wild type helical morphology (Fig. 3A). The mutated 12‐1 and 33‐1 *pgp1* alleles and upstream sequence equivalent to the *pgp1* region in pEF38R (Fig. 1A) were cloned into pRRC in the reverse orientation, as in pEF38R. Introduction of the mutated 12‐1 and 33‐1 *pgp1* alleles could not restore the helical shape in a *pgp1* deletion strain (Fig. 3B). The 25‐1 *pgp2* allele was cloned into pRRC using the primers used to generate pJV4 (Frirdich *et al*., 2014) used for *pgp2* complementation and integrated in the *pgp2* deletion strain. The 25‐1 allele did not complement a *pgp2* mutant (Fig. 3B).

### The muropeptide profile of the 81‐176 33‐1 (Pgp1 S402F) and 25‐1 (Pgp2 G131C) isolates resemble that of a *pgp1* and *pgp2* deletion strain respectively

Since 33‐1 and 25‐1 lack a functional *pgp1* and *pgp2* allele, respectively, it was expected that the muropeptide profile would be identical to that of the corresponding deletion mutant. PG was isolated from *C. jejuni* 81‐176 wild type, 25‐1, 25‐6, 33‐1, and 33‐2, and the muropeptides were separated by HPLC (Table 2, Supporting Information Table S4). The muropeptide changes between the straight isolates and wild type were compared with those of Δ*pgp1* and Δ*pgp2* analyzed previously (Frirdich *et al*., 2012; 2014). The 25‐1 isolate had a similar muropeptide profile to that of Δ*pgp2* with decreased dipeptides, no tripeptides, and increased tetrapeptides. The 33‐1 isolate muropeptide profile resembled that of Δ*pgp1* with similar changes in muropeptide species: a decrease in dipeptides, an increase in tripeptides and decrease in tetrapeptides in the monomeric, dimeric, and trimeric forms. These results are consistent with the sequencing data identifying a SNP in *pgp2* in 25‐1 and a SNP in 33‐1 in *pgp1*. The muropeptide profile of 81‐176 12‐1 was not analyzed, but would likely resemble that of 33‐1. Helical 81‐176 that had been passaged on CFW (isolates 25‐6 and 33‐2) showed a relative increase in O‐acetylation levels and decrease in tripeptides in comparison with 81‐176 that had been grown only on MH.

**Table 2:** Summary of the muropeptide composition of *C. jejuni* wild‐type 81‐176 passaged on CFW (25‐1, 25‐6, 33‐1, 33‐2) in comparison with 81‐176 wild type, Δ*pgp1* (Frirdich *et al*., 2012) and Δ*pgp2* (Frirdich *et al*., 2014) mutant strains characterized previously.^a^

	*C. jejuni* strains								
						81‐176	81‐176	81‐176	81‐176
	81‐176	Δ*pgp1*	81‐176	Δ*pgp2*	81‐176	25‐1	25‐6	33‐1	33‐2
Shape	Helical	Straight	Helical	Straight	Helical	Straight	Helical	Straight	Helical
Date analyzed	09/2010	09/2010	01/2011	01/2011	08/2013	08/2013	08/2013	08/2013	08/2013
Strain compared with		81‐176		81‐176		25‐6	81‐176	33‐2	81‐176
		09/2010		01/2011		08/2013	08/2013	08/2013	08/2013
Muropeptide species	% Peak area^b^								
Monomers (total)	43.3	43.4	41.6	40.8	42.2	41.5	43.0	41.2	40.0
Di	14.5	**5.9** *	15.3	**9.1** *	13.6	**8.0** *	15.2	**7.4** *	14.1
Tri	10.5	**34.4** *	8.9	**0.0** *	10.1	**0.5** *	8.3 (18%)	**29.5** *	8.1*
Tetra	18.4	**3.1** *	17.7	**31.7** *	17.2	**31.5** *	18.2	**3.6** *	16.5
PentaGly^5^	nd^d^	nd	nd	nd	0.9	**1.0** *	0.8	**0.4** *	0.9
Acetylated^c^	3.3	**5.1** *	2.9	1.6	1.9	3.2	**3.7** *	2.4	**2.9** *
Dimers (total)	49.7	52.5	47.9	47.6	48.8	49.8	48.2	50.0	49.7
Tetra tri	14.7	**37.0** *	16.0	**0.0** *	16.0	**0.0** *	12.7*	**33.9** *	15.0
Tetra tetra	33.5	**14.7** *	31.1	**46.8** *	32.0	**49.0** *	34.9	**15.7** *	33.8
Tetra pentaGly^5^	1.5	**0.7** *	0.7	0.8	0.8	0.8	0.7	**0.5** *	0.9
Anhydro	10.8	11.7	12.5	12.1	11.8	12.5	10.6	12.6	12.5
Acetylated^c^	4.8	**6.8** *	2.4	0.5	3.2	5.0	**4.8** *	3.7	3.8
Trimers (total)	6.9	**4.2** *	10.5	11.6	7.8	8.7	7.9	**6.0** *	9.1
Tetra tetra tri	0.8	**1.9** *	1.0	**0.0** *	1.0	**0.0** *	0.8*	**2.1** *	1.0
Tetra tetra tetra	6.1	**2.3** *	9.5	11.6	6.8	8.7	7.1	**3.9** *	8.1 (16%)
Dipeptides (total)	14.5	**5.9** *	15.3	**9.1** *	13.6	**8.0** *	15.2	**7.4** *	14.1
Tripeptides (total)	18.1	**53.6** *	17.3	**0.0** *	18.4	**0.5** *	14.9 (19%)	**47.1** *	15.9 (14%)
Tetrapeptides (total)	66.7	**40.2** *	67.0	**90.5** *	65.1	89.7*	67.4	**41.8** *	67.0
Pentapeptides (total)	0.7	**0.4** *	0.4	0.4	1.2	1.3	1.1	**0.7** *	1.3
Acetylated (total)^c^	5.7	**8.5** *	4.1	1.8	3.5	5.7	**6.1** *	4.3	4.8*
Anhydro chain ends (total)	6.8	6.4	7.9	8.0	7.7	8.1	7.1	8.0	8.3
Average chain length	14.7	15.7	12.7	12.5	13.0	12.4	14.1	12.5	12.0
Degree of cross‐linkage	29.5	29.0	31.0	31.6	29.6	30.7	29.4	29.1	30.9
% Peptides in cross‐links	56.7	56.6	58.4	59.2	57.8	58.5	57.0	58.8	60.0
						**Similar to Δ*pgp2***	**Similar to 81‐176**	**Similar to Δ*pgp1***	**Similar to 81‐176**

**a.**The morphology of each strain is indicated below the strain description, as is the date of analysis and the strain to which comparisons were made.

**b.** Numbers represent the percent area of each muropeptide from Table S4 calculated to give a total of 100%. Values indicated with an asterisk (*) or both bolded and with an asterisk, represent a greater than or equal to 20% or 30% difference, respectively in comparison with the strain mentioned in the table.

**c.** The values for the percentage of O‐acetylated species do not represent the true level of O‐acetylation in these strains, as most of these substitutions are lost in the standard alkaline reduction procedure used in this study to prepare the PG. These values were included to demonstrate the relative difference in O‐acetylation between the samples.

**d.** nd = not determined.

### Whole genome sequencing (WGS) to examine the effect of growth on CFW

WGS was used to determine whether CFW was having mutagenic effects on our *C. jejuni* strains. Genomic comparisons were carried out between 81‐176 strains passaged on CFW (*dim111*, *dim128*, 12‐1, 12‐10, 25‐1, 25‐6, 33‐1, and 33‐2) to our non‐CFW passaged wild type. In addition, WGS was used to compare our laboratory strain of *C. jejuni* 81‐176 to the published sequence available in GenBank (NC_008787).

There were a total of 32 variants in 14 different genes and 9 in intergenic regions in our wild type 81‐76 strain in comparison with the published 81‐176 sequence, with 12 of the 32 changes occurring at homopolymeric tracts (Supporting Information Table S3). The *cjj81176_1449* gene of unknown function showed a large amount of variation, with 6 individual SNPs and 2 substitutions and 1 frameshift at a variant frequency ranging from 18.9% to 37.50%. The same 9 changes in *cjj81176_1449* were also present in the CFW passaged strains at a slightly higher variant frequency ranging from 26.8% to 54.7%. A SNP occurring at a variant frequency of 34.6% in the *recR* gene (*cjj81176_1279*) involved in the recombinational process of DNA repair was also present in all other sequenced strains at frequencies ranging from 27.1% to 41.1%. The *cjj81176_1449* and *recR* genes are therefore likely to be highly variable genes in the *C. jejuni* 81‐176 population. Three SNPs were present at variant frequencies under 20%. SNPs in *cjj81176_0227* (*purF*), *cjj81176_1105*, and *cjj81176_1354* occurred at frequencies of 94.9%, 100% and 100%, respectively and were present in all other strains sequenced, indicating that they were present in the starting strain and maintained during passage.

The *dim* mutants sequenced (*dim111* and *dim128*) harbored a deletion in *cjj81176_1105* at a variant frequency of 95.1–99.3% (Supporting Information Table S3) and this deletion was responsible for the changes in cell shape observed in these strains (as described above). These mutants showed an accumulation of a greater total number of genomic changes (57, *dim111*; and 53, *dim128*) than the 81‐176 strains passaged on CFW (34 in 12‐1; 32 in 12‐10; 40 in 25‐1; 31 in 25‐6; 36 in 33‐1; and 39 in 33‐2) and the wild type 81‐176 (32 total changes). The *dim* mutants had 10 genomic changes not present in the other strains at frequencies of 15.6–58.6% in *cjj81176_0477*, the gene encoding the DNA polymerase III subunit epsilon responsible for the 3′‐5′ exonuclease activity. The *dim* mutants also showed the occurrence of more SNPs in *cjj81176_1397*, the *feoB* gene responsible for ferrous iron acquisition (Naikare *et al*., 2006) with strains 25‐6 and 33‐2 having one SNP in *feoB*. All these SNPs occurred at very low frequencies (<25%). Excluding the SNPs in *pgp1* (in 12‐1 and 33‐1) and *pgp2* (in 25‐1) that occurred at 98.9–100%, the 81‐176 strains passaged on CFW (12‐1, 12‐10, 25‐1, 25‐6, 33‐1, and 33‐2) did have a few genomic changes not present in the wild type strain, but they were not consistent across the strains and occurred at low frequencies (11 of them <25% and 3 of them <36%) (Supporting Information Table S3).

### The *pgp1* gene of the straight 11168‐GS (genome sequenced) strain is non‐functional

**Figure 4:**
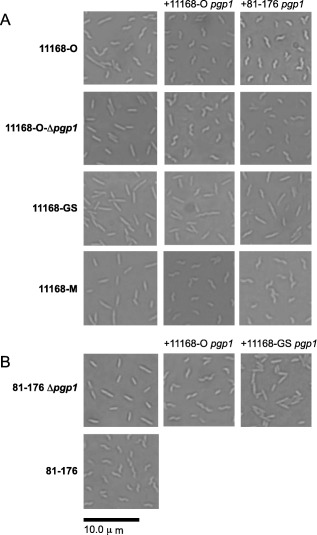
Cellular morphology and complementation analysis of *C. jejuni* 11168‐O (original), 11168‐GS (genome sequenced), and a *pgp1* mutant strain in 11168‐O. DIC microscope images of *C. jejuni* strains grown on solid media for 18 h. A. cellular morphology of the helical 11168‐O strain and straight 11168‐O‐Δ*pgp1*, 11168‐GS, and 11168‐M strains. The *pgp1* and *pgp2* genes were sequenced in 11168‐O, 11168‐GS, and 11168‐M. 11168‐GS and 11168‐M had a frameshift mutation in *pgp1* resulting in a premature stop codon. For complementation analysis, the *pgp1* gene from 11168‐O and 81‐76 was introduced at the rRNA spacer region. The *C. jejuni* 81‐176 region used for *pgp1* complementation was that from plasmid pEF38R indicated in Fig. 1B. A similar region from 11168 was used. B. complementation analysis of an 81‐76 *pgp1* mutation demonstrating complementation from the functional 11168‐O gene and non‐functional 11168‐GS gene.

The *C. jejuni* NCTC11168 strain was originally isolated from a human diarrheic patient in 1977 [11168‐O (original)] (Skirrow, 1977) and then sequenced in 2000 [11168‐GS (genome sequenced)] (Parkhill *et al*., 2000). The 11168‐O and 11168‐GS strains have several phenotypic differences decreasing the virulence of the 11168‐GS strain (Gaynor *et al*., 2004). The 11168‐O strain is helical while the laboratory passaged 11168‐GS variant has lost its helical morphology and is rod‐shaped, similar to a *pgp1* mutant constructed in 11168‐O (Fig. 4A). The *pgp1* and *pgp2* genes of 11168‐GS and 11168‐O were sequenced and compared with the NCTC 11168 sequence deposited in GenBank (accession number NC_002163). A G nucleotide was deleted in *pgp1* at position 157 of a total of 1395 nucleotides in the 11168‐GS from our laboratory collection (Supporting Information Fig. S1), but not the 11168 sequence deposited in GenBank (Supporting Information Table S5). This change resulted in a frameshift mutation and truncated Pgp1 protein (the predicted length of Pgp1 is 56 amino acids in comparison with the 464 amino acid full‐length Pgp1). The *pgp2* gene sequences were identical in all strains (Supporting Information Table S5). To demonstrate that 11168‐GS *pgp1* was non‐functional, the 11168‐GS *pgp1* gene was expressed in 81‐176 Δ*pgp1*. Unlike the 11168‐O *pgp1* gene, 11168‐GS *pgp1* could not complement a 81‐176 Δ*pgp1* mutant (Fig. 4B). Despite 7 amino acid differences between the *pgp1* gene of 81‐176 and 11168‐O (Supporting Information Fig. S1), either gene could complement the shape phenotype of 11168‐O‐Δ*pgp1* (Fig. 4A). Neither 81‐176 nor 11168‐O *pgp1* alone could restore the wild type helical shape of 11168‐GS; however, some of the cells did regain a very slight degree of helicity (Fig. 4A). WGS was carried out to determine whether another peptidase was affected in 11168‐GS that would affect complementation to wild type morphology (see below).

A 11168‐O strain microaerophilically passaged 13 times reported previously by our laboratory (Gaynor *et al*., 2004), referred to here as 11168‐M (microaerophilically passaged), has a straight morphology due to a deletion in *pgp1* resulting in a frameshift mutation, identified by WGS (Supporting Information Table S5). The 11168‐M morphology defect could be complemented back to a wild type helical morphology by introduction of *pgp1* (Fig. 4A). It should be noted that the exact nature of how this strain was generated is unknown, as repeated attempts at passaging 11168‐O over 25 times under microaerophilic conditions did not result in a straight strain.

### The 11168‐GS muropeptide profile is similar to that of 11168‐O‐Δ*pgp1*

**Figure 5:**
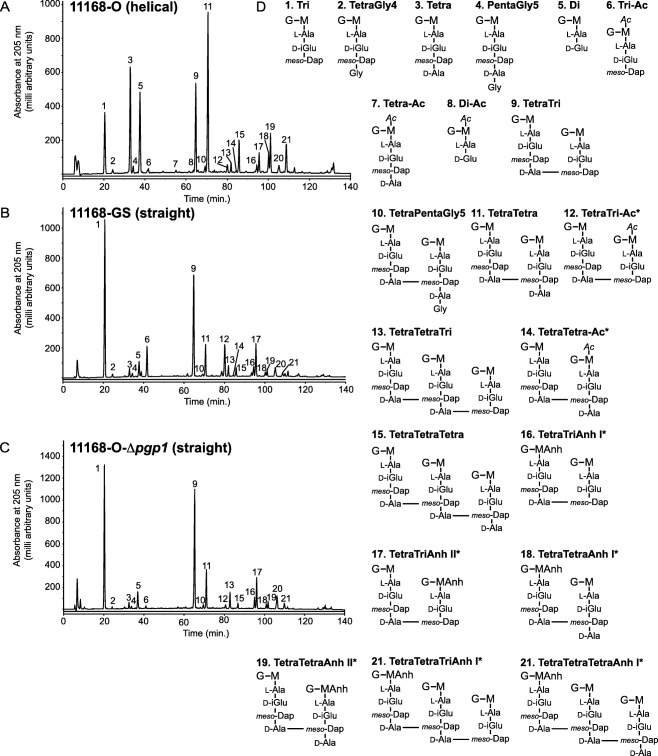
HPLC elution profile of *C. jejuni* 11168‐O, 11168‐GS, and 11168‐O‐Δ*pgp1* muropeptides. Purified PG from strains all grown for 1 day was digested with cellosyl and the resulting muropeptides were reduced with sodium borohydride and separated on a Prontosil 120‐3‐C18 AQ reverse‐phase column. HPLC profiles are shown for A, *C. jejuni* 11168‐O strain with helical morphology; B, 11168‐GS strain with straight morphology; and C, the 11168‐O‐Δ*pgp1* mutant strain with straight morphology. Peak numbers correspond to the main muropeptide peak fractions of *C. jejuni* 81‐176 analyzed by LTQ‐FT‐MS (Frirdich *et al*., 2012), with two additionally characterized peaks, and shown in panel D. G, *N*‐acetylglucosamine; M, reduced *N*‐acetylmuramic acid; l‐Ala, l‐alanine; d‐iGlu, d‐isoglutamic acid; d‐Glu, d‐glutamic acid; *meso‐*DAP, *meso‐*diaminopimelic acid; Gly, Glycine; Ac, O‐acetyl groups at the C‐6 hydroxyl group of MurNAc; Anh, 1,6‐anhydro group at MurNAc. The asterisk (*) indicates that it is not known on which MurNAc residue the modification occurs.

PG was isolated from 11168‐GS, 11168‐O, and 11168‐O‐Δ*pgp1* and the muropeptides were separated by HPLC (Fig. 5, Table 3, Supporting Information Table S4) to compare the changes between helical 11168‐O and straight 11168‐GS and whether these were consistent with the changes in a 11168‐O‐Δ*pgp1* mutant. As with 81‐176 Δ*pgp1*, 11168‐GS and 11168‐O‐Δ*pgp1* showed a decrease in dipeptides, increase in tripeptides and decrease in tetrapeptides in both the monomeric and multimeric forms. Despite the inability of *pgp1* to completely restore the helical shape of 11168‐GS, 11168‐GS and 11168‐O‐Δ*pgp1* had very similar muropeptide profiles. The relative levels of O‐acetylation in 11168‐GS were 5.1 fold higher than 11168‐O, while 11168‐O‐Δ*pgp1* O‐acetylation was 2.8‐fold lower than 11168‐O. Analysis of the O‐acetylation levels would have to be carried out to determine the exact differences, as these substitutions are lost by the standard alkaline reduction procedures used in this study to analyze the muropeptide composition. The muropeptide profiles of 11168‐O and 81‐176 were relatively similar.

**Table 3:** Summary of the muropeptide composition of the *C. jejuni* wild‐type 11168‐O (original), 11168‐GS (genome sequenced) and 11168‐O‐Δ*pgp1* mutant strain. The 81‐176 Δ*pgp1* (Frirdich *et al*., 2012) mutant strain used for comparison was characterized previously.^a^

	*C. jejuni* strains							
	11168‐O	11168‐GS	11168‐GS	11168‐O	11168‐O Δ*pgp1*	81‐176	81‐176	81‐176 Δ*pgp1*
Shape	Helical	Straight	Straight	Helical	Straight	Helical	Helical	Straight
Date analyzed	01/2013	01/2013	01/2013	08/2013	08/2013	08/2013	09/2010	09/2010
Strain compared with		11168‐O 01/2013	11168‐O Δ*pgp1* 08/2013		11168‐O 08/2013	11168‐O 08/2013		81‐176 09/2010
Muropeptide species	% Peak area^b^							
Monomers (Total)	40.8	44.0	44.0	42.6	38.7	42.2	43.3	43.4
Di	12.8	**3.3** *	3.3*	15.9	**4.3** *	13.6	14.5	**5.9** *
Tri	10.3	**37.8** *	37.8	8.2	**32.1** *	10.1	10.5	**34.4** *
Tetra	16.1	**1.6** *	1.6	17.1	**1.4** *	17.2	18.4	**3.1** *
PentaGly^5^	1.0	**0.7** *	0.7	0.9	**0.6** *	0.9	nd^d^	nd
Acetylated^c^	1.3	**6.5** *	**6.5** *	1.8	**0.6** *	1.9	3.3	**5.1** *
Dimers (Total)	47.6	48.4	48.4	47.8	51.5	48.8	49.7	52.5
Tetra tri	16.6	**39.6** *	39.6	15.5	**39.9** *	16.0	14.7	**37.0** *
Tetra tetra	30.0	**8.4** *	8.4*	31.4	**10.6** *	32.1	33.5	**14.7** *
Tetra pentaGly^5^	1.0	**0.4** *	**0.4** *	0.9	1.0	0.8	1.5	**0.7** *
Anhydro	11.2	11.4	11.4	12.2	12.2	11.8	10.8	11.7
Acetylated^c^	1.5	**7.8** *	**7.8** *	2.4	**0.9** *	3.2*	4.8	**6.8** *
Trimers (total)	10.1	**4.7** *	4.7*	8.2	6.0*	7.8	6.9	**4.2** *
Tetra tetra tri	1.2	**1.9** *	**1.9** *	1.0	**3.4** *	1.0	0.8	**1.9** *
Tetra tetra tetra	8.9	**2.8** *	2.8	7.2	**2.7** *	6.8	6.1	**2.3** *
Dipeptides (Total)	12.8	**3.3** *	3.3*	15.9	**4.3** *	13.6	14.5	**5.9** *
Tripeptides (Total)	19.0	**58.2** *	58.2	16.3	**53.2** *	18.4	18.1	**53.6** *
Tetrapeptides (Total)	64.6	34.1	34.1	64.6	**37.3** *	65.1	66.7	**40.2** *
Pentapeptides (Total)	1.5	**0.9** *	0.9*	1.4	1.1*	1.2	0.7	**0.4** *
Acetylated (Total)^c^	2.0	**10.4** *	**10.4** *	3.1	**1.1** *	3.5	5.7	**8.5** *
Anhydro chain ends (Total)	7.7	7.0	7.0	8.0	7.9	7.7	6.8	6.4
Average chain length	13.0	14.3	14.3	12.4	12.7	13.0	14.7	15.7
Degree of cross‐linkage	30.5	27.3	27.3	29.4	29.8	29.6	29.5	29.0
% Peptides in cross‐links	59.2	56.0	56.0	57.4	61.3	57.8	56.7	56.6

**a.**The morphology of each strain is indicated below the strain description, as is the date of analysis and the strain to which comparisons were made.

**b.** Numbers represent the percent area of each muropeptide from Table S4 calculated to give a total of 100%. Values indicated with an asterisk (*) or both bolded and with an asterisk, represent a greater than or equal to 20% or 30% difference, respectively in comparison with the strain mentioned in the table.

**c.** The values for the percentage of O‐acetylated species do not represent the true level of O‐acetylation in these strains, as most of these substitutions are lost in the standard alkaline reduction procedure used in this study to prepare the PG. These values were included to demonstrate the relative difference in O‐acetylation between the samples.

**d.** nd = not determined.

### WGS to compare 11168‐O and 11168‐GS and our laboratory strain of 11168‐GS to the published sequence of 11168‐GS

WGS allowed comparison between our laboratory strain of *C. jejuni* 11168‐GS and the published sequence available in GenBank [NC_002163 (Parkhill *et al*., 2000)]. There were a total of 25 variants in 20 different genes and 2 in intergenic regions in our 11168‐GS strain (Supporting Information Table S5). Of the 25 variants, 10 occurred at homopolymeric tracts, 13 were SNPs, one was the deletion in *cj1345c* (*pgp1*) and one was an insertion in *cj0628* (a putative lipoprotein). Excluding the expected variants in homopolymeric tracts, 8 of the changes occurred at very low frequencies <26%, one change in *cj0628* (a putative lipoprotein) occurred at a frequency of 74.1%, and the remaining 6 [in *cj0431* (putative periplasmic ATP/GTP‐binding protein), *cj0437* (*sdhA*), *cj0807* (putative oxidoreductase), *cj1262* (*racS*), *cj1345c* (*pgp1*) and *cj1401c* (*tpiA*)] occurred at frequencies >98%. The mutation in *pgp1*, as described above, is responsible for the straight morphology of our laboratory strain.

Our laboratory 11168‐O strain had 36 changes in 28 different genes in comparison with the 11168‐GS published sequence, with 5 of the changes in intergenic regions. Of the 36 changes, 20 were in homopolymeric tracts, 15 were SNPs, and there was one insertion in *cj0945c* (a putative helicase). Excluding the changes in homopolymeric tracts or in intergenic regions, 8 of the changes occurred at very low frequencies of <34%, one change in *cj1259* (*porA*, the major outer membrane protein) occurred at a frequency of 71.3%, and the remaining 5 [in *cj0276* (*mreB*), *cj0284c* (*cheA*), *cj0431* (putative periplasmic ATP/GTP‐binding protein), *cj0455* (novel glycopeptide), and *cj0807* (putative oxidoreductase)] occurred at frequencies >98% (summarized in Supporting Information Table S6). There were 6 changes common to both our laboratory 11168‐O and 11168‐GS strains: changes in *cj0431*, *cj0807*, *cj0935c* (2 changes; putative sodium:amino‐acid symporter family protein), *cj1263* (*recR*), and *cj1437c* (aminotransferase). Changes in *cj0276* (*mreB*), *cj0284c* (*cheA*), *cj0431* (putative periplasmic ATP/GTP‐binding protein), *cj0455* (novel glycopeptide), *cj0807* (putative oxidoreductase), and *cj1259* (*porA*, the major outer membrane) also occurred in NCTC 11168‐BN148, a laboratory variant of 11168‐GS obtained from the Centers of Disease Control in 1979 and with invasion, motility and morphology phenotypes resembling 11168‐O and not 11168‐GS (Revez *et al*., 2012). As seen in the genome sequencing of 81‐176, the *recR* gene was also variable in 11168. The *C. jejuni* 11168 homolog (*cj1456c*) of the 81‐176 gene (*cjj81176_1449*) that was variable in 81‐176 was not variable in 11168.

The motile strains 11168‐O and 11168‐BN148 (Revez *et al*., 2012; Supporting Information Table S5) had a SNP in *cj0284c* (*cheA*) resulting in an amino acid substitution of I290T that was not present in our laboratory 11168‐GS strain (non‐motile) or the published 11168‐GS strain. Mutants in *cheA* are non‐motile (Golden and Acheson, 2002), so the amino acid substitution may explain the motility defect of 11168‐GS.

### Examination of the basis why expression of the 11168‐O pgp1 gene does not restore wild‐type helical morphology to 11168‐GS

The 11168‐GS strain expressing wild type Pgp1 produced cells with a very minimal helical pitch (Fig. 4A). This could result from: (1) differences in *pgp1* expression levels affecting the levels of complementation, (2) mutation in a 11168‐GS gene responsible for dictating helical pitch, or (3) alterations in the abundance of proteins affecting cell wall and cell shape biogenesis, or (3) differences in pgp1 expression levels affecting the levels of complementation. Experiments were carried out to address these hypotheses.

#### Differences in levels of Pgp1

**Figure 6:**
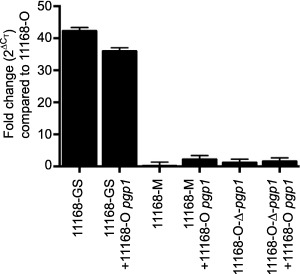
Expression of the *pgp1* gene is higher in a 11168‐GS background than in 11168‐M or 11168‐O strains. RNA was extracted from rod shaped *C. jejuni* strains 11168‐GS, 11168‐GS expressing 11168‐O *pgp1*, 11168‐M, and 11168‐O Δ*pgp1*, and helical strains 11168‐O, 11168‐M overexpressing 11168‐O *pgp1*, and 11168‐O Δ*pgp1* overexpressing 11168‐O *pgp1* grown in broth for 24 h post‐inoculation at OD 0.002. The transcript levels of the *pgp1* gene were examined by q‐RT‐PCR and relative quantification was carried out by normalizing against unit mass (10 ng cDNA). Relative expression was calculated by the ΔC_T_ method and fold differences are displayed in comparison with the helical 11168‐O strain. Reactions were performed in triplicate on three independent samples.

Since overexpression of *pgp1* as well as deletion of *pgp1* results in cell straightening (Frirdich *et al*., 2012), the proper levels of Pgp1 are required for wild‐type helical morphology. Genetic expression levels of *pgp1* were examined by real‐time quantitative PCR (RT‐qPCR) in 11168‐O, 11168‐GS, 11168‐GS expressing 11168‐O *pgp1*, 11168‐M, 11168‐M overexpressing 11168‐O *pgp1*, 11168‐O‐Δ*pgp1*, 11168‐O‐Δ*pgp1* expressing 11168‐O *pgp1* (Fig. 6). Primers used for RT‐pPCR bound outside of the area deleted in 11168‐O‐Δ*pgp1*. The 11168‐GS strains showed much higher expression levels of *pgp1* than the 11168‐M and 11168‐O‐Δ*pgp* strains. Expression levels of *pgp1* were identical to those of 11168‐O in 11168‐M, 11168‐M overexpressing 11168‐O *pgp1*, 11168‐O‐Δ*pgp1*, and 11168‐O‐Δ*pgp1* expressing 11168‐O *pgp1*.

#### Identification of genomic changes in 11168‐GS potentially affecting the helical pitch of 11168‐GS helical morphology

WGS of 11168‐GS, 11168‐O, 11168‐M and the published sequence of NCTC 11168‐BN148 were compared with determine whether another PG peptidase or shape determining gene could be altered in 11168‐GS that would prevent complementation to wild type morphology (Supporting Information Table S5). The morphology of the sequenced strain of 11168 deposited in GenBank (Parkhill *et al*., 2000) is most likely helical (since it did not have a frameshift mutation in *pgp1* like our laboratory version of this strain), although the degree of helical pitch in this strain is unknown. Therefore, genomic changes from Supporting Information Table S5 present in >50% of the population that are either unique to our laboratory strain of 11168‐GS or unique to the helical 11168‐O strain and ‐M strain that could be complemented to a helical morphology were identified (summarized in Supporting Information Table S6) as possible causes for the lack of complete restoration of morphology by introduction of *pgp1* into 11168‐GS.

Interestingly, 11168‐O, 11168‐M, and 11168‐BN148 all had a SNP in *cj0276* (*mreB*) (present in 98.8% and 100% of the 11168‐O and 11168‐M population, respectively) resulting in amino acid substitution D48G that was present in neither our laboratory 11168‐GS strain nor the published 11168‐GS (Supporting Information Table S6). MreB from *C. jejuni* 81‐176 has also a Gly residue at position 48. Since MreB is generally the major determinant of cell shape in non‐spherical bacteria and is involved directly or indirectly in positioning the PG synthesis machinery (reviewed in Typas *et al*., 2012; Errington, 2015), it is conceivable that an amino acid change in MreB could alter its function or its ability to interact properly with its protein interaction partners thereby affecting the helical shape of *C. jejuni*. However, introduction of 11168‐O *mreB* at the rRNA spacer region of 11168‐GS expressing 11168‐O *pgp1* did not result in wild type helical morphology (data not shown). Conversely, expressing 11168‐GS *mreB* at the rRNA spacer region in 11168‐O followed by deletion of the 11168‐O *mreB* had no effect on morphology (data not shown).

WGS also identified a SNP in the *cj0455c* gene of 11168‐O and 11168‐M present at 98.7% and 100%, respectively, that resulted in extension of the gene product by 61 amino acids (*115Q, where the asterisk represents the stop codon). In a study using proteomics and glycoproteomics to compare 11168‐O to 11168‐GS, the *cj0455c* gene was found to encode a glycopeptide with >100‐fold increase in 11168‐O in comparison with 11168‐GS (Scott *et al*., 2014). Cj0455c is a putative membrane protein of unknown function. Expression of 11168‐O *0455* at the rRNA spacer region of 11168‐GS expressing 11168‐O *pgp1* also had no effect on morphology (data not shown).

Our laboratory strain of 11168‐GS differed from the sequenced 11168‐GS strain, 11168‐O and 11168‐M strains in the *cj1306c* gene. Our laboratory variant had a frameshift mutation resulting from a deletion of a C nucleotide at a tandem repeat, resulting in a C9 to a C8 mutation leading to a premature stop codon truncating the protein from 409 amino acids to 202 amino acids. Cj1306c and the downstream gene product Cj1305c show 78%/86% identity/similarity, respectively, and both are putative hydrolases with a peptidase S28 domain. Expression of 11168‐O *1305*‐*1306* at the rRNA spacer region of 11168‐GS expressing 11168‐O *pgp1* had no effect on morphology (data not shown).

#### Identification of proteomic changes potentially affecting cell wall or cell shape in 11168‐GS

A proteomic study carried out by Scott *et al*. (2014) indicated that the Cj0796c putative PG hydrolase along with MurF (Cj0795c) were found in higher amounts in 11168‐O than in 11168‐GS (Scott *et al*., 2014). Cj0796c contains an alpha/beta hydrolase fold, as with Cj1305c. MurF (Cj0795c) is a UDP‐*N*‐acetylmuramoyl‐tripeptide d‐alanyl‐d‐alanine ligase responsible for ligating the d‐Ala‐d‐Ala dipeptide to UDP‐*N*‐acetylmuramoyl‐tripeptide generating UDP‐MurNAc pentapeptide in PG precursor synthesis. Ddl (11168 Cj0798c) just upstream of 11168 Cj0796c is also involved in PG precursor biosynthesis. It is a d‐alanyl‐d‐alanine ligase catalyzing the formation of d‐alanyl‐d‐alanine from two d‐alanine residues. The annotation of Cj0796c as a putative hydrolase and its location near PG precursor biosynthetic proteins indicate that it could also be involved in PG biosynthesis. To examine the potential influence of levels of the Cj0796c protein on morphology, the 11168‐O *0796* gene was expressed at the rRNA spacer region of 11168‐GS expressing 11168‐O *pgp1*. Most transformants showed no change in morphology (Supporting Information Fig. S4). Interestingly, approximately 1/10 transformants produced filamented cells with wild‐type helical morphology. These pick‐up suppressors easily and lose their filamented phenotype upon passage. Transformation of genomic DNA from filamented, helical 11168‐GS strains expressing 11168‐O *pgp1* resulted in a ratio of filamented cells with wild‐type helical morphology to rod‐shaped cells of 1/10 seen, as seen with the initial transformation. Preliminary attempts at constructing a 11168‐GS deletion mutant were unsuccessful, indicating that Cj0796c may be essential, which is supported by the literature (Metris *et al*., 2011).

### The 11168‐GS variant is phenotypically different from 11168‐O and a 11168‐O‐*pgp1* mutant

**Figure 7:**
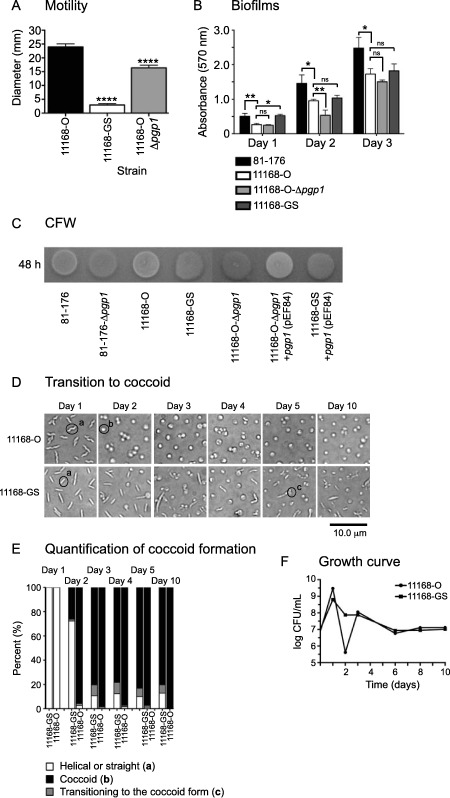
The *C. jejuni* 11168‐GS and 11168‐O‐Δ*pgp1* strains have a defect in motility, biofilm formation and delayed transition to the coccoid form in comparison with 11168‐O. A. 11168‐GS and 11168‐O‐Δ*pgp1* exhibited a 67.6% and 37.0% decrease in motility in comparison with 11168‐O, respectively, as assayed by measuring halo diameters in soft agar plates. Standard error of the mean was calculated from 6 measurements. B. 11168‐GS showed an increase in biofilm formation in comparison with 11168‐O at Day 1 and then similar levels thereafter, while 11168‐O‐Δ*pgp1* was defective for biofilm formation in comparison with 11168‐O at Day 2 and Day 3. Biofilm formation was assessed by crystal violet staining of standing cultures in borosilicate tubes and quantification of dissolved crystal violet at 570 nm. Standard errors of the mean were calculated from triplicate cultures and are representative of three independent experiments. C. similar to 81‐176 Δ*pgp1*, 11168‐GS and 11168‐O‐Δ*pgp1* showed a hypofluorescent phenotype on CFW in comparison with the wild type fluorescence of 81‐176 and 11168‐O after growth for 48 h on plates containing 0.002% CFW. Introduction of 11168 *pgp1* restored wild type fluorescence to the 11168‐O Δ*pgp1* mutant, but did not affect the fluorescence of 11168‐GS. D. DIC microscope images of 11168‐O and 11168‐GS grown on solid media at 38°C to follow the transition to the coccoid form over time. E. the percentage of helical, coccoid and cells transitioning to the coccoid form as determined from DIC images such as those shown in D. At least three separate fields of view of approximately 100–200 bacteria were counted for each strain at each time point and this was carried out in triplicate. Representative cells considered to be helical, coccoid or transitioning to the coccoid form are indicated by a, b, or c, respectively in the DIC images in D. F, growth curve analysis of *C. jejuni* wild type 11168‐O and 11168‐GS grown in broth at timepoints corresponding to those used for DIC microscopy (D) and quantification for coccoid formation (E). 11168‐O shows a drop in viability in comparison with 11168‐GS at Day 2, but no differences thereafter, despite continuing differences in population morphology. The asterisk (*) indicates a statistically significant difference using the unpaired Student's *t*‐test, with *, **, or **** indicating *p* < 0.05, *p* < 0.01, and *p* < 0.0001 respectively.

Some of the phenotypes found to be defective in a rod shaped *C. jejuni* 81‐176 mutant were examined in 11168 (Frirdich *et al*., 2012): motility, biofilms, CFW reactivity and the transition to a coccoid form. As determined previously, the 11168‐GS strain was defective for motility in soft agar, displaying 12.1% motility compared with 11168‐O. Similar to 81‐176 Δ*pgp1* (Frirdich *et al*., 2012), 11168‐O‐Δ*pgp1* had a slight motility defect at 68.5% of 11168‐O motility (Fig. 7A). The level of biofilm production was assessed over 3 days using the crystal violet assay (Fig. 7B). The 11168‐O strain produced less biofilm than 81‐176. In comparison with 11168‐O, biofilm levels of 11168‐GS were consistently 2.0‐fold higher at Day 1 and then identical to 11168‐O at Day 2 and 3. The 11168‐O‐Δ*pgp1* strain produced less biofilms than the 11168‐O strain only at Day 2. The CFW fluorescence of 11168‐O was identical to that of 81‐176 (Fig. 7C), both being helical strains with a wild type copy of *pgp1*. The 81‐176 Δ*pgp1* mutant was hypofluorescent on CFW (as mentioned above; Frirdich *et al*., 2012), as were the 11168‐O Δ*pgp1* mutant and 11168‐GS strains, expected from their straight phenotype resulting from the absence of Pgp1 activity. The 11168‐O Δ*pgp1* mutant could be complemented to wild type CFW fluorescence by insertion of 11168 *pgp1* at the rRNA locus (from plasmid pEF84). Introduction of 11168 *pgp1* (from plasmid pEF84) into 11168‐GS did not complement the morphology defect, or the CFW hypofluorescence.

*Campylobacter jejuni* transitions to a coccoid form during exposure to environmental stresses such as aging, starvation, suboptimal temperatures, changes in oxygen tension, pH, osmolarity and pressure (reviewed in Svensson *et al*., 2008; Ikeda and Karlyshev, 2012). For ease of analysis, aging was used as the condition of choice for coccoid formation. Cells were grown at 38°C on solid media under microaerophilic conditions and coccoid formation was monitored by microscopy over time (Fig. 7D). All samples were taken from the center of the plate, as sampling from different areas of the plate showed some variability. The percentage of helical, coccoid, and cells transitioning to the coccoid form was quantified from DIC images (Fig. 7E). After 2 days post‐inoculation, approximately 96% of the 11168‐O population had transformed from the helical to coccoid form on solid media, while only 26% of 11168‐GS was coccoid. The percentage of coccoid bacteria in the 11168‐O strain gradually increased to 100% by Day 10 (98% at Day 3, 4, and 5 and up to 100% at Day 10). By Day 3, 11168‐GS was 80% coccoid and remained at 80% up to and including Day 10. The delay in coccoid formation observed for 11168‐GS was identical to that seen for a Δ*pgp1* mutant in 81‐176 (E. Frirdich and E. Gaynor, manuscript in preparation). Growth curve analysis (Fig. 7F) was used to assess the viability of *C. jejuni* 11168‐O and 11168‐GS at long‐term timepoints used to examine the transition to the coccoid form in Fig. 7D and E. The viability of 11168‐O and 11168‐GS was similar at all days despite differences in population morphology, except at Day 2. The drop in 11168‐O viability at Day 2 (48 h) was observed consistently and was also observed in *C. jejuni* NCTC 11351 (Martinez‐Rodriguez *et al*., 2004). A maximum cell count is reached upon entering stationary phase at Day 1 (24 h) of 3.0 × 10^9^ for 11168‐O (2.0 × 10^9^ for 11351), followed by a decline to 4.2 × 10^5^ (10^6^ for 11351) at Day 2 (48 h), and then an increase at Day 3 (72 h) to 1.1 × 10^8^ (10^6^ for 11351) (Fig. 7F; Martinez‐Rodriguez *et al*., 2004). For 11168‐GS, the maximum cell count of 6.4 × 10^8^ is also reached at Day 1 (24 h) which is followed by a decline to 7.5 × 10^7^ at Day 2 (48 h) and no further increase in viability beyond the Day 2 timepoint. Strain passage (such as that used to generate 11168‐GS from 11168‐O) has been noted to result in a reduced rate of viability loss in stationary phase and lack of a re‐growth phase (Martinez‐Rodriguez *et al*., 2004), explaining the differences in growth curves for 11168‐O and 11168‐GS.

## Discussion

Variations in virulence between *C. jejuni* strains can at least be partly attributed to the large degree of genetic and resulting phenotypic differences between isolates (van Putten *et al*., 2009). *C. jejuni* genetic variation not only occurs between different strains, but also within individual strains, as we have shown in this study. Therefore, *C. jejuni* is considered to have a partly non‐clonal population structure with some clones remaining stable and others showing genetic instability arising during growth. This has been typically attributed to the natural ability of *C. jejuni* to take up and integrate DNA, intragenomic rearrangements, and phase variation (Wassenaar, 2002). The *C. jejuni* intra‐strain genetic diversity in turn generates phenotypic variation that is hypothesized to increase the ability of some members of the offspring generation to survive varying environmental stresses encountered during transmission under which *C. jejuni* cannot replicate. The surviving clone(s) would then be able to colonize a new host, and clonal expansion would occur, facilitating bacterial survival. *C. jejuni* genomic instability may therefore serve as a method of rapid adaptation to different environmental conditions and an alternative strategy to more conventional genetic regulatory mechanisms for sensing the environment and modifying gene expression accordingly. Cell‐to‐cell heterogeneity, as seen in *C. jejuni*, has been identified as a widespread microbial phenomenon that can be regulated and is typically observed in genes associated with stress and metabolic functions, and not essential genes (reviewed in Martins and Locke, 2015).

Previously, the *C. jejuni* genome was shown to encode hypervariable regions or contingency loci that could not be resolved to a consensus sequence during WGS (Parkhill *et al*., 2000). These hypervariable regions include clusters of genes, such as those involved in the synthesis of the LOS and capsule, flagellar modification and DNA restriction and modification systems (Parkhill *et al*., 2000). The hypervariable genes contain sequences of short runs of homopolymeric nucleotides (Parkhill *et al*., 2000) prone to a high rate of slipped‐strand mispairing (SSM) during replication. This results in insertions and/or deletions and subsequent frameshift mutations that switch gene function on/off at a rate that is higher than that of spontaneous mutation, creating a subpopulation within the bacterial population. These mutations are heritable and usually reversible. SSM can also occur in promoter regions thereby affecting promoter activity and the expression levels of downstream proteins. SSM is a mechanism of phase variation, a process of generating bacterial population heterogeneity. Phase variation is widespread among bacterial genera of different taxonomic groups and influences bacterial adaptation and virulence by modulating the synthesis of surface exposed molecules such as capsule, flagella, pili, adhesins, and iron‐acquisition factors, affecting processes such as motility, biofilm formation and detachment, adhesion, host recognition and evasion of host immune responses (Wisniewski‐Dye and Vial, 2008; Darmon and Leach, 2014). Phase variation can also modulate genes involved in metabolism, regulation and DNA restriction‐modification systems (Wisniewski‐Dye and Vial, 2008; Darmon and Leach, 2014). *C. jejuni* displays a high rate of phase variation (Bayliss *et al*., 2012), occurring at poly‐G and poly‐C tracts (Parkhill *et al*., 2000), as well as in shorter poly‐A tracts (Hendrixson, 2006, 2008) with the frequency of variation depending on the tract length (Bayliss *et al*., 2012). *C. jejuni* also lacks a functional DNA mismatch repair (MMR) system responsible for correcting replication errors from single base pair mismatches or SSM arising at homopolymeric tracts, likely adding to the mutation rate seen in *C. jejuni* and increasing the degree of phase variation (Parkhill *et al*., 2000; Gaasbeek *et al*., 2009). Another factor involved in influencing the population heterogeneity and the mutation rate of *C. jejuni* is the *Campylobacter mfd* (mutation frequency decline) gene product encoding a transcription‐repair coupling factor (Han *et al*., 2008). In *Escherichia coli*, Mfd was found to be linked to a “mutation frequency decline” phenotype, preventing the formation of mutations by mediating DNA repair at DNA lesions that stall RNA polymerase during transcription (Savery, 2007; Ganesan *et al*., 2012). In contrast, *C. jejuni* Mfd was found to actually promote the appearance of spontaneous mutants (Han *et al*., 2008). How *C. jejuni* Mfd affects mutation rates in *C. jejuni* and whether other proteins are involved in the process is currently unknown.

An increasing number of studies are demonstrating the genomic instability and adaptable mutative properties of *C. jejuni*. Comparison of the whole genome sequence of a minimally passaged *C. jejuni* 81‐176 strain (81‐76/55) (Mohawk *et al*., 2014) and our laboratory 81‐176 strain to the reference 81‐76 strain sequenced by The Institute for Genomic Research (TIGR) revealed mutations throughout the genome. These included variations at homopolymeric tracts, SNPs, indels (short insertions or deletions), and substitutions. Non‐motile colonial variants of *C. jejuni* 81‐176/55 were isolated by media passaging and were found to result from mutations in the two component regulatory system *flgRS* regulating flagellar expression [previously reported to be phase variable (Hendrixson, 2006; 2008)] and from high frequency spontaneous mutations in the *motA* gene coding for the flagellar motor protein (Mohawk *et al*., 2014). Another study observed differential motility between defined mutant clones in the *flaB* gene of *C. jejuni* M1 (de Vries *et al*., 2015). Genome sequencing identified second site mutations consisting of SNPs and indels that were responsible for the motility defects observed in some of the *flaB* mutants (de Vries *et al*., 2015). This highlights the importance of the examination of several mutant clones and complementation to establishing *C. jejuni* mutant phenotypes. In this study, exposure to the stress of growth on CFW resulted in selection of *C. jejuni* 81‐176 clones with a straight morphology due to mutations in the *pgp1* and *pgp2* genes. These mutations included SNPs in *pgp1* and *pgp2*, and SSM in *pgp1* at poly‐A and ‐G tracts, as well as at a duplicated sequence. Reversion of the *pgp1^‐^/pgp2^‐^* mutants was not seen by passage on MH. Since straight and helical *C. jejuni* grow equally well on MH, this is not surprising. Reversion may be seen under conditions favoring a helical morphology, such as during chick colonization.

All 81‐176 strains sequenced in this study showed variation in *cjj81176_1449* and *recR* gene (*cjj81176_1279*), indicating that these genes are likely to be highly variable in *C. jejuni* 81‐176. The *recR* gene was also varied in *C. jejuni* 11168 strains sequenced, but the 11168 homolog of *cjj81176_1449* was not. The *cjj81176_1449* gene is unique to *Campylobacter* species and encodes a hypothetical protein of unknown function. RecR is a recombination mediator protein involved in the homologous recombination pathway of chromosomal DNA repair which is important in maintaining genomic stability. It is conceivable that changes in *C. jejuni* RecR would affect DNA recombinational repair and could influence the generation of genetic diversity. In *H. pylori*, RecR functions with RecO to form the RecRO pathway of recombinational repair which was shown to mediate intra‐genomic recombination at direct repeat sequences and repair DNA damage in response to oxidative and acid stress (Wang *et al*., 2011). *H. pylori* mutants in *recR* and *recO* were also defective in host colonization (Wang *et al*., 2011). The importance of genomic variability in *C. jejuni recR* and *cjj81176_1449* remains to be examined.

It is unknown why passage of the wild type strain on CFW favored accumulation of SNPs in *pgp1* or *pgp2*, while passage of Tn mutants on CFW favored frameshift mutations in *pgp1* (Table 1). A subsequent Tn mutant screen on CFW likewise identified SNPs in *pgp1* and *pgp2* in Tn mutants unlinked to the Tn insertion (J. Vermeulen, E. Frirdich, and E. Gaynor, unpublished). All frameshift mutants resulted in truncated versions of Pgp1 (Table 1) thereby abrogating function. The point mutations in *pgp1* and *pgp2* also produced non‐functional proteins. The Pgp1 S402F and V175F substitutions in 81‐176 rod shaped isolates 33‐1 and 12‐1, respectively, were not in active site residues of Pgp1, as determined by conserved domain analysis and comparison with the crystal structure of the *H. pylori* Csd4 homolog (Chan *et al*., 2015), but are required for Pgp1 activity. Introducing a Phe preserves the hydrophobic nature of the wild type amino acids, but is also more bulky and may push the protein apart. The V175F mutation is in the N‐terminal carboxypeptidase domain located on a helix away from the active site (Chan *et al*., 2015), but may have an allosteric effect on activity. The S402F mutation is in a semi‐flexible surface loop region in the last domain (domain 3) that is required for activity and predicted to be involved in protein–protein or protein–PG interactions (Chan *et al*., 2015). The point mutation in Pgp2 resulted in a G131C. There is no crystal structure available for Pgp2, but the amino acid change is in the conserved ld‐catalytic domain YkuD as predicted from conserved domain analysis (Supporting Information Fig. S3). Changing a Gly to a Cys could cause considerable changes in protein structure that could affect activity such as: a reduction in flexibility of that region which may be required for function or the formation of disulfide bonds between Cys residues resulting in a loss of protein stability and aggregation. All mutated copies of *pgp1* and *pgp2* could be complemented with a wild type copy of the gene.

The nature of the stress imposed by CFW on *C. jejuni* is unclear as well as why straight mutants are more prevalent after growth on CFW. Helical and straight strains both grow equally well in media with and without CFW and have a similar MIC to CFW (data not shown), so CFW‐induced selection of straight variants is not due to differences in growth rate. Since straight mutants all display a *dim* phenotype on CFW and therefore fail to bind CFW, this must be somehow advantageous to the cell. CFW does bind to the β 1‐4 linkages of the PG backbone (E. Frirdich and E. Gaynor, unpublished). CFW binds equally well to isolated PG from wild type and Δ*pgp1* (E. Frirdich and E. Gaynor, unpublished); however, this may not be the case with cellular PG. The differences in the length of the PG amino acid side chains in a straight mutant in comparison with the helical strain may alter the accessibility of CFW to the PG backbone, limiting CFW binding and the deleterious effects of CFW on the cell.

The selection of straight variants after passage on CFW can be attributed to either: (1) the presence of straight cells in the original *C. jejuni* 81‐176 population prior to exposure to CFW, or (2) the development of straight variants after exposure to CFW. In the first scenario, examination of the cellular morphology of a population of *C. jejuni* 81‐176 should show the presence of straight cells among the helical cells even at a very low percentage. This has not been observed in our laboratory at any stage of *C. jejuni* 81‐176 growth (E. Frirdich and E.C. Gaynor, unpublished). In the second scenario, variation in *pgp1* and *pgp2* would occur likely by an increase in the level of phase variation (as in the *pgp1* frameshift mutations) and mutation rate (to generate SNPs, as in 12‐1, 25‐1, and 33‐1) after exposure to CFW. Environmental or intracellular host factors (such as temperature, pH, nutrient availability, oxygen levels, and the availability of iron) have been shown to affect the rate and timing of phase variation in other bacteria, thereby controlling the generation of variants and increasing the appearance of subpopulations that are more likely to successfully survive the stress (van der Woude and Broadbent, 2011; Darmon and Leach, 2014). WGS of strains passaged on CFW (transposon mutants *dim111* and *dim128*, and 81‐176 variants 12‐1, 12‐10, 25‐1, 25‐6, 33‐1, and 33‐2; Supporting Information Table S3) did not display any genomic changes unique to CFW exposure when compared with a non‐CFW passaged wild‐type 81‐176 strain. In addition, initial proteomic studies with 81‐176 grown with and without CFW showed no observable differences in protein abundance (N.E. Scott, E. Frirdich, J. Vermeulen, and E.C. Gaynor, unpubl. obs.). Unlike the CFW‐passaged 81‐176 variants, the *dim* mutants selected for on CFW had an increased total number of genomic changes in comparison with non‐CFW passaged 81‐176 (78% for *dim111*, and 66% for *dim128*) (Supporting Information Table S3). The process of Tn mutagenesis or maintenance of the Tn may have induced additional variation in these strains.

Our current hypothesis is that the binding of CFW to *C. jejuni* PG results in envelope stress that triggers the upregulation of phase variation and increases mutation rates in *C. jejuni*, thereby resulting in *pgp1* and *pgp2* mutations that produce *C. jejuni* variants more tolerant to the effects of CFW. To further clarify this we need to determine the nature of the stress posed by CFW on *C. jejuni*, how envelope stress is recognized in *C. jejuni*, and whether phase variation is regulated in *C. jejuni*. Passaging of *C. jejuni* NCTC 11168 through mice (Jerome *et al*., 2011), chickens (Bayliss *et al*., 2012) and through humans (after accidental infection) (Revez *et al*., 2013; Thomas *et al*., 2014), resulted in the selection of variants often displaying increased virulence. The variants were primarily at homopolymeric tracts in phase variable contingency genes, although one study did identify the presence of SNPs in the genome (Thomas *et al*., 2014). The variants isolated after human infection were distinct from those isolated from animal models (Revez *et al*., 2013) and a human infection isolate when inoculated into mice underwent additional genetic variation (Thomas *et al*., 2014). This emphasizes the important role that *C. jejuni* genetic heterogeneity plays in adaptation to different host environments. Interestingly, substantial genetic changes only occurred during host passage and not during growth *in vitro* (Bayliss *et al*., 2012; Kim *et al*., 2012; Thomas *et al*., 2014), supporting the idea that phase variation may be regulated in *C. jejuni*.

Sequence analysis showed that the nature of the straight morphology of the 11168‐GS strain characterized previously in our laboratory (Gaynor *et al*., 2004) resulted from a frameshift mutation in *pgp1*. This frameshift mutation occurred due to the loss of a G nucleotide at a –GG– sequence (Supporting Information Fig. S1). Whether this occurred due to SSM is difficult to determine, as a rough threshold of four repeat units has been proposed as the minimum number resulting in mutation by SSM (Zhou *et al*., 2014). The straight 11168‐GS variant of the helical 11168‐O strain is thought to have arisen due to laboratory passage (Gaynor *et al*., 2004), although repeated passage of 11168‐O (up to 25 times) in our laboratory has not resulted in the appearance of a straight 11168‐O variant (E. Frirdich and E.C. Gaynor, unpublished). The 11168‐GS strain may therefore have been subjected to additional stresses during laboratory passage that resulted in the selection of this straight variant. With the now relative ease of WGS, several complete genome sequences of NCTC 11168 are currently available and have been used to identify genetic changes affecting virulence by comparing more virulent strains to the avirulent 11168‐GS strain (Jerome *et al*., 2011; Revez *et al*., 2013, 2012; Cooper *et al*., 2013). None of the genome sequenced 11168 strains [including the 11168‐GS deposited in GenBank by Parkhill *et al*. (2000)] have mutations in *pgp1*, indicating that the 11168‐GS strain used by our laboratory may be the only variant with a morphology defect. This further highlights the fact that NCTC 11168 strains vary greatly between laboratories, making experimental comparisons extremely difficult.

The similar muropeptide profiles between the 11168‐GS strain and a 11168‐O‐Δ*pgp1* mutant indicate that the frameshift mutation in 11168‐GS *pgp1* resulted in an inactive version of the Pgp1 protein and that this loss of Pgp1 activity is responsible for the straight morphology of the 11168‐GS strain. However, unlike the 11168‐O‐Δ*pgp1* mutant and the 11168‐M strain also harboring a frameshift mutation in *pgp1*, introduction of a wild type copy of *pgp1* was unable to restore the wild type helical morphology to 11168‐GS and produced cells with a very minimal helical pitch. This could result from several factors. (1) Differences in the levels of Pgp1 [increases and decreases in Pgp1 levels affect morphology (Frirdich *et al*., 2012)]. The level of *pgp1* expression was higher in 11168‐GS and 11168‐GS overexpressing 11168‐O *pgp1* than the 11168‐O strain. The levels of *pgp1* in 11168‐O were similar to those of the rod‐shaped 11168‐M and 11168‐O‐Δ*pgp1* strains, as well as the 11168‐M and 11168‐O‐Δ*pgp1* strains overexpressing 11168‐O *pgp1* that could be complemented to a wild‐type helical morphology. Therefore, the higher level of Pgp1 in 11168‐GS strains could be preventing proper shape complementation. (2) Mutation in a gene responsible for dictating the degree of helical pitch (candidates are summarized in Supporting Information Table S6). Of the ones examined, cell shape determinant *mreB* (cj0276), hydrolase *cj1305c* and the glycopeptide *cj0455c*, none were able to restore wild type helicity. Lastly, (3) a change in abundance of proteins affecting the cell wall and cell shape (Scott *et al*., 2014). Several proteins affecting peptidoglycan biosynthesis and thereby morphology were present in lower amounts in 11168‐GS in comparison with 11168‐O: the penicillin binding‐proteins PbpA and PbpC, the tubulin‐like cell division protein FtsZ [previously shown to affect cell shape (Varma and Young, 2004)], the amino acid ligase MurF involved in UDP‐MurNAc pentapeptide precursor synthesis (Scott *et al*., 2014), and the putative hydrolase Cj0796/Cjj81176_0817 (although its role in cell morphology remains to be established). It is possible that the levels of these proteins could affect morphology.

Despite the similar cellular morphology and PG muropeptide profiles between our 11168‐GS strain and a 11168‐O‐Δ*pgp1* mutant, other phenotypes associated with a deletion of *pgp1* [such as motility and biofilm formation; (Frirdich *et al*., 2012)] that are altered in comparison with wild type 11168‐O also differ between 11168‐O‐Δ*pgp1* and our 11168‐GS strain (Fig. 7). This can be attributed to the numerous other genomic changes (Gaynor *et al*., 2004) and differences in protein abundance (Scott *et al*., 2014) between 11168‐GS and 11168‐O.

It is now well accepted that *C. jejuni* isolates consist of a dynamic population of varying genotypes and therefore phenotypes that undergo diversification during exposure to stress and different host environments. This population heterogeneity allows for rapid adaptation and survival under new conditions. The appearance of rod shaped variants, such as those arising from exposure to CFW described in this study, our laboratory 11168‐GS strain (Gaynor *et al*., 2004), those generated by passage through chick embryos (Field *et al*., 1991, 1993) and chickens (Hanel *et al*., 2009), and those that appeared in some flagellar mutants (Wassenaar *et al*., 1991; Matz *et al*., 2002; Fernando *et al*., 2007) that are unlinked to the flagellar mutation indicate that the ability of *C. jejuni* to alter its shape from helical to straight provides a selective advantage to *C. jejuni* under certain stress conditions. The exact nature of this stress remains to be determined. The *pgp1* gene can now be added to the repertoire of phase variable genes in *C. jejuni*. Loss of *pgp1* and the associated changes in PG structure affect numerous *C. jejuni* pathogenic attributes and recognition by host receptors, so it is not surprising that changes in this gene and in morphology would affect how *C. jejuni* interacts with its environment.

## Experimental procedures

### Bacterial strains and growth conditions

The bacterial strains and plasmids used in this study and their construction are described in the Supplemental files (Supporting Information Tables S1 and S2). *C. jejuni* strains were grown at 38°C in Mueller‐Hinton (MH; Oxoid) broth or on 8.5% (w/v) agar plates supplemented with vancomycin (V; 10 μg/ml) and trimethoprim (T; 5 μg/ml) (unless otherwise indicated) under microaerobic and capnophilic conditions (6% O_2_, 12% CO_2_) in a Sanyo tri‐gas incubator for plates or using the Oxoid CampyGen system for broth cultures. Growth media was supplemented with chloramphenicol (20 μg/ml), kanamycin (50 μg/ml), and apramycin (60 μg/ml) where appropriate. *E. coli* strains used for plasmid construction were grown at 38°C in Luria‐Bertani (LB; Sigma) broth or 7.5% agar (w/v) agar plates and supplemented with ampicillin (100 μg/ml), chloramphenicol (15 μg/ml), or kanamycin (25 μg/ml), as necessary.

For growth analysis and RNA analysis, *C. jejuni* strains were streaked from fresh plate cultures and grown for 5–7 h. They were harvested in MH‐TV broth and inoculated at an OD_600_ of 0.002 into MH‐TV broth and grown shaking for 18 h. Strains were subcultured to an OD_600_ of 0.002 and samples were taken at various time points for CFU and microscopic analysis.

### Microscopy

For visualization under bright field or differential interference contrast (DIC) microscopy, 1 μl of a broth or plate culture was immobilized on a thin 1% agar (w/v in H_2_0) slab and overlaid with a cover slip. Images were captured with a Nikon Eclipse TE2000‐U microscope equipped with 100× objective and a Hamamatsu Orca camera system. Quantification of the percentage of helical, coccoid, and cells transitioning to the coccoid form in the DIC images was carried out by counting the number of each using ImageJ software (NIH). At least three separate fields of view of approximately 150–200 bacteria were counted for each strain at each time point and this was repeated for three separate cultures.

### Whole‐genome sequencing

Genomic DNA for all experiments was harvested via Wizard genomic DNA purification (Promega). Illumina libraries were prepared using the KAPA Low‐Throughput Library Preparation Kit with Standard PCR Amplification Module (Kapa Biosystems, Wilmington, MA), following manufacturer's instructions except for the following changes: 750 ng DNA was sheared using an M220 instrument (Covaris, Woburn, MA) in 50 μl screwcap microtubes at 50 peak power, 20 duty factor, 20°C, 200 cycles per burst and 25 s duration. Adapter ligated fragments were size selected to 700–800 bp following Illumina protocols. Standard desalting TruSeq LT and PCR Primers were ordered from Integrated DNA Technologies (Coralville, IA) and used at 0.375 µM and 0.5 µM final concentrations respectively. PCR was reduced to 4 cycles. Libraries were quantified using the KAPA Library Quantification Kit (Kapa), except with 10 µl volume and 90 s annealing/extension PCR, then pooled and normalized to 4 nM. Pooled libraries were re‐quantified by ddPCR on a QX200 system (Bio‐Rad), using the Illumina TruSeq ddPCR Library Quantification Kit and following manufacturer's protocols, except with an extended 2 min annealing/extension time. The libraries were sequenced 2 × 250 bp paired end v2 on a MiSeq instrument (Illumina) at 13.5 pM, following manufacturer's protocols. The MiSeq reads were reference assembled to the genome of the background strains *C. jejuni* 81‐76 (NC_008787) or NCTC 11168 (AL11116) using Geneious 9.1 reference assembler (Biomatters, Auckland, NZ).

### Isolation of straight *C. jejuni* 81‐176 isolates by passage on CFW

A similar protocol to the CFW screen used to identify Tn mutants with altered CFW reactivity (McLennan *et al*., 2008; Frirdich *et al*., 2012) was carried out to isolate straight *C. jejuni* 81‐176 variants (outlined in Fig. 2). Since CFW binds to sugars with β 1,3 and β 1,4 linkages, CFW fluoresces with the starch in MH media, so BHI lacking starch is used in a CFW screen. Even though fluorescence on CFW was not measured in this study, BHI was still used in case the BHI had an effect on cell shape. Passage on BHI without CFW was used as a control. *C. jejuni* 81‐176 was passaged twice from frozen stock on MH plates and harvested into BHI. The culture was diluted to give approximately 2000 CFU/ml (a 10e‐5 dilution of a starting culture at an OD of 0.3) and 100 μl aliquots were spread plated onto BHI and BHI‐CFW (a final concentration of 0.002% CFW was used). The plates were incubated for 48 h under microaerophilic conditions at 38°C. Colonies were patched onto MH, the cell morphology was examined after growth for 18–24 h, and the colonies were repatched to either BHI or BHI‐CFW. After the second passage on BHI or BHI‐0.002% CFW, plates were incubated for either 1 or 2 days, patched onto MH and the cell morphology was examined after 18–24 h. Any straight variants were restreaked for isolated colonies which were examined for cell morphology.

### Phenotypic analyses: motility, biofilm formation, and CFW reactivity

Phenotypic assays were carried out with strains grown in shaking MH‐TV broth for 18 h. For motility, cultures were diluted to an OD_600_ of 0.2 in MH‐TV and 2 μl was point inoculated into MH‐TV plates containing 0.4% agar. Plates were incubated for 20 h and the halo diameter was measured. Biofilm formation assayed using crystal violet staining and CFW fluorescence was described previously (Frirdich *et al*., 2012).

### Peptidoglycan isolation and muropeptide analysis

PG isolation, muropeptide generation, separation by HPLC and identification was carried out as described previously (Frirdich *et al*., 2012; 2014). Briefly, *C. jejuni* strains were passaged once from frozen stocks, passaged to 20‐25 MH plates and grown for 18–20 h. Cells were collected into cold MH broth by scraping to give a final OD of 200–600, harvested by centrifugation at 8000 × *g* for 15 min and then resuspended in 6 ml ice cold H_2_O. Cells were lysed by dropwise addition to 6 ml 8% SDS boiling under reflux. PG was purified from the cell lysate, digested with the muramidase cellosyl (kindly provided by Hoechst, Frankfurt, Germany), and the resulting muropeptides were reduced with sodium borohydride and separated by HPLC as described (Glauner, 1988). Muropeptide structures were assigned based on (i) comparison with retention times of known muropeptides from *C. jejuni* (Frirdich *et al*., 2012) and (ii) by mass spectrometry (MS). For MS analysis, muropeptide fractions were collected, concentrated in a SpeedVac, acidified by 1% trifluoroacetic acid, and analyzed by offline electrospray mass spectrometry on a Finnigan LTQ‐FT mass spectrometer (ThermoElectron, Bremen, Germany) at the Newcastle University Pinnacle facility as described (Bui *et al*., 2009).

### Real‐time quantitative PCR

*Campylobacter jejuni* strains were grown as described above. After 24 h of growth from a culture inoculated at an OD600 of 0.002, RNA was isolated and cDNA was synthesized as described (Svensson *et al*., 2009). Absence of genomic DNA within RNA samples was confirmed by PCR and by including a no reverse transcriptase control in the cDNA reactions. Quantitative PCR was performed using primers designed to *pgp1* (1344‐SYBR‐F2/‐R2; Supporting Information Table S2). Reactions were set up with IQ SYBR green Supermix (Bio‐Rad) and performed with a CFX96 real‐time PCR detection system (Bio‐Rad) as directed by the manufacturer. Reactions were performed in triplicate on three independent samples. Relative quantification was carried out by normalizing against unit mass (10 ng cDNA) and relative expression of *pgp1* was calculated by the ΔC_T_ method. A reference gene was not used for relative quantification as the amplification efficiency calculated from a standard curve with various template preparations and different sets of primers to the commonly used *C. jejuni* reference genes *rrs* (16S rRNA; Hyytiäinen *et al*., 2012) or *rpoA* (α‐subunit of DNA‐directed RNA polymerase; (Ritz *et al*., 2009) was too low (<90%).

## Supporting information

Supporting Information
