# Supplementary material for: Morphology heterogeneity within a Campylobacter jejuni helical population: the use of calcofluor white to generate rod‐shaped C. jejuni 81‐176 clones and the genetic determinants responsible for differences in morphology within 11168 strains
Source: Mol Microbiol. 2017 Apr 24;104(6):948–71. doi: 10.1111/mmi.13672 (PMC5530802; doi:10.1111/mmi.13672)
Supplement: Supplementary file 1 — Supporting Information [file MMI-104-948-s001.pdf]

Supplementary Information for:

Morphology heterogeneity within a *Campylobacter jejuni* helical population: the use of calcofluor white to generate rod-shaped *C. jejuni* 81-176 clones and the genetic determinants responsible for differences in morphology within 11168 strains.

Emilisa Frirdich[1#], Jacob Biboy[2], Steven Huynh[3], Craig T. Parker[3], Waldemar Vollmer[2], and Erin C. Gaynor[1]

[1]Department of Microbiology and Immunology, University of British Columbia, Vancouver, BC, V6T 1Z3, Canada

[2]The Centre for Bacterial Cell Biology, Institute for Cell and Molecular Biosciences, Newcastle University, Newcastle upon Tyne, NE2 4AX, United Kingdom

[3]Produce Safety and Microbiology Research Unit, Agricultural Research Service, U.S. Department of Agriculture, Albany, CA, 94710, USA

#Corresponding author:
Tel: 604-827-5796
Fax: 604-822-6041
Email: emilisa@mail.ubc.ca

Content

Supplemental Materials and Methods

Table S1. Bacterial strains or plasmids used in this study.

Table S2. Primers used in this study.

Table S3. Genomic changes relative to the *C. jejuni* 81-176 reference sequence in GenBank (NC_008787) of our laboratory strain of *C. jejuni* 81-176 and derivatives of 81-176 passaged on CFW: transposon mutants with a slight loss of helical morphology, *dim111* and *dim128*, and variants of 81-176 with helical (12-10, 25-6 and 33-2) and straight (12-1, 25-1 and 33-1) morphologies.

Table S4. Muropeptide composition of *C. jejuni* wild-type 81-176, 25-1, 25-6, 33-1, 33-2, 11168-O, 11168-GS and 11168-O-Δ*pgp1* mutant strain.

Table S5. Genomic changes relative to NCTC 11168-GS in Gen Bank (NC_002163; (Parkhill *et al.*, 2000) in our laboratory stocks of the 11168-O (original) and 11168-GS (genome sequenced strain), and 11168-M strain (a 11168-O strain microaerophilically passaged 13 times).

Table S6. Genomic changes relative to NCTC 11168-GS in Gen Bank (NC_002163; (Parkhill *et al.*, 2000) in our laboratory stocks of the 11168-O (original) and 11168-GS (genome sequenced strain), and 11168-M strain (a 11168-O strain microaerophilically passaged 13 times) that are either unique to our laboratory strain of 11168-GS or unique to 11168-O and 11168-M.

Figure S1. Clustal O (1.2.1) alignment of the *C. jejuni pgp1* gene and gene product from the helical wild type 81-176 strain, straight 81-176 strains derived from CFW passage, helical 11168-O and straight 11168-GS.

Figure S2. The change in morphology of C. jejuni 81-176 Tn mutants dim111 and dim128 are not due to the Tn insertion, but are a result of a deletion mutation in cjj81176_1105.

Figure S3. Clustal O (1.2.1) alignment of the *C. jejuni pgp2* gene and gene product from the wild type 81-176 strain and 81-176-derived strain 25-1.

Figure S4. Expression of 11168-O 0796 in a 11168-GS strain expressing 11168-O pgp1 results in helical filaments in 10% of the colonies.

References

Supplemental Materials and Methods

Construction of *C. jejuni pgp1* and *pgp2* expression and complementation strains

For complementation studies with *pgp1* and *pgp2*, the gene was PCR amplified from genomic DNA and cloned into the pRRC vector that integrates at the rRNA spacer region with varying amounts of the upstream region. The *pgp1* gene was cloned with varying amounts of upstream sequence (3816 bp in pEF35R and 1310 bp in pEF38R, pEF80R, pEF82R, pEF83R and pEF84R). The *pgp1* gene in the above plasmids was cloned in the reverse orientation as the promoter for the antibiotic resistance cassette (designated with an R) to prevent overexpression of *pgp1* (Frirdich *et al.*, 2012). Construction of the pEF35R coding the *pgp1*-1348 region was described previously (Frirdich *et al.*, 2012). Plasmids pEF38R, pEF80R, pEF82R, pEF83R and pEF84R were constructed by amplifying the *pgp1* gene and upstream *1345* gene from *C. jejuni* 81-176, 81-176 33-1, 81-176 12-1, 11168-GS and 11168-O genomic DNA, respectively, with primers 1346-1 (*SpeI*) and 1344-15 (*SpeI*). The *SpeI*-digested PCR product was ligated to pRRC digested with *XbaI* and dephosphorylated. The resulting construct was verified for orientation by PCR and sequenced. The *C. jejuni* 81-176 *pgp2* gene was cloned with 196 bp of upstream sequence in the same orientation as the pRRC CmR cassette to form pJV4 (Frirdich *et al.*, 2014). A similar construct was cloned using 81-176 25-1 genomic as the template. The *pgp2* gene and 196 bp of the upstream sequence containing the putative promoter were PCR amplified with primers 0915-JV-6 (*SpeI*) and 0915-JV-2 (*MfeI*). The PCR product was digested with *SpeI* and *MfeI* and ligated to pRRC digested with *XbaI* and *MfeI*. The resulting construct was verified by PCR and sequencing. Plasmids were inserted into *C. jejuni* strains by natural transformation and transformants selected on the appropriate antibiotics. Single insertions into the rRNA spacer region were verified by PCR with primers ak233, ak234, ak235 (Karlyshev & Wren, 2005) and cat-2 for pRRC. The sequence of the gene at the *pgp1/pgp2* locus and in the rRNA spacer region were confirmed by sequencing.

Construction of *C. jejuni Δ1105* mutant and *1105* complementation strains

The *1105* gene was PCR amplified with iProof (Biorad) from 81-176 genomic DNA using primers 1105-L1 and 1105-R1. A polyA tag was added to the PCR product and it was ligated to a commercially available pGEM-T vector (Promega). The resulting construct pGEMT-*1105* was verified by restriction enzyme digestion and sequencing. The *aphA3* cassette was digested out of pUC18K-2 with *XbaI* and *KpnI* and ligated to the *XbaI* and *KpnI* digested inverse PCR product of pGEMT-*1105* amplified with primers 1105-IL1 (*KpnI*) and 1105-IR1 (*XbaI*), deleting 358 bp from the middle of the gene, forming pGEMT-*1105*Km. Orientation of the Km resistance cassette was verified by restriction enzyme digestion and sequencing. *C. jejuni* 81-176 was naturally transformed with pGEMT-*1105*Km. 81-176 Δ*1105* mutant strains were selected by a Km^R phenotype and verified by PCR and restriction enzyme digestion.

For complementation studies with *1105*, the gene and 288 bp of upstream DNA was PCR amplified from 81-176 genomic DNA with 1105-L2 (*XbaI*) and 1105-R2 (*MfeI*), digested by restriction sites encoded by the primers, and cloned into the similarly digested pRRC vector to form plasmid pRRC-*1105*. The resulting construct was verified by PCR and sequenced. The *1105* gene was cloned in the same orientation as the promoter for the antibiotic resistance cassette. Plasmids were inserted into *dim111* and *dim128* by natural transformation and transformants

were selected on the appropriate antibiotics. Single insertions into the rRNA spacer region were verified by PCR with primers ak233, ak234, ak235 and cat-2.

Construction of *C. jejuni* 11168-O-Δ*pgp1*

To generate a Δ*pgp1* mutant in 11168-O, 11168-O was naturally transformed with 81-176 Δ*pgp1* genomic DNA and transformants were selected by their Km^R phenotype and verified by PCR analysis.

Complementation with *mreB*

Construction of pRRA-11168-O *mreB* and pRRA-11168-GS *mreB* expression vectors

The *mreB* genes of 11168-O and 11168-GS with 68 bp of upstream DNA were PCR amplified from genomic DNA using primers mreB-FWDC (*XbaI*) and mreB-REVC (*MfeI*), digested with sites introduced into the primers and cloned into pRRA digested with similar restriction enzymes. The resulting plasmids were verified by PCR and sequencing and renamed pEF89 (pRRA-11168-GS *mreB*) and pEF90 (pRRA-11168-O *mreB*).

Introduction of 11168-O *mreB* into 11168-GS+*pgp1-1345*$_{\mathbf{11168\text{-}O}}$

The pEF90 (pRRA-11168-O *mreB*) plasmid was methylated (Donahue *et al.*, 2000) with 11168-GS cell free extract and introduced into 11168-GS+ *pgp1-1345*$_{11168\text{-}O}$ by electroporation. It was selected for on MH-TV-Apr_{60}-Cm_{15}. Insertion of the 11168-O *mreB* gene into the rRNA spacer region was verified by PCR analysis and sequencing, as was the maintenance of *pgp1*.

Introduction of 11168-GS *mreB* into 11168-O and deletion on 11168-O *mreB*

The pEF89 (pRRA-11168-GS *mreB*) plasmid was methylated (Donahue *et al.*, 2000) with 11168-O cell free extract and introduced into 11168-O by natural transformation. The native 11168-O *mreB* gene was then inactivated using the pEF101 plasmid constructed previously in attempts to construct a *mreB* deletion in the *C. jejuni* strain 81-176. The pEF101 plasmid is a derivative of pEF100. For pEF100, the *mreB* gene was PCR amplified with iProof (Biorad) from 81-176 genomic DNA using primers mreB-1 and mreB-2, a polyA tag was added to the PCR product and it was ligated to a commercially available pGEM-T vector (Promega). The resulting plasmid was verified by restriction enzyme digestion and sequencing. The *aphA3* cassette was digested out of pUC18K-2 with *KpnI* and *HincII* and ligated to the *KpnI* and *EcoRV* digested inverse PCR product of pEF100 amplified with primers mreB-9 (*KpnI*) and mreB-10 (*EcoRV*), deleting 887 bp from the middle of the gene, forming pEF101. The *C. jejuni* strain 11168-O+*mreB*$_{11168\text{-}GS}$ was naturally transformed with pEF101. The 11168-O-Δ*mreB*+ *mreB*$_{11168\text{-}GS}$ strain was selected by a Apr^RKm^R phenotype and verified by PCR and sequencing.

Expression of 11168-O *0455, 1305-1306* and *0796*

The *C. jejuni* 11168-O *0455, 0796,* and *1306-1305* genes were PCR amplified from genomic DNA using primers 0455-1 (*XbaI*) and 0455-2 (*MfeI*), 0796-1 (*XbaI*) and 0796-2 (*MfeI*), and 1305/6-1 (*XbaI*) and 1305/6-2 (*MfeI*), respectively, digested with sites introduced into the primers and cloned into pRRK digested with similar restriction enzymes. The resulting plasmids were verified by PCR and sequencing and renamed pEF91 (pRRK+*0455*), pEF92 (pRRK+*0796*)and pEF93 (pRRK+*1306-1305*). The plasmids were introduced into 11168-GS+ *pgp1-1345*$_{11168\text{-}O}$ by electroporation, selected by a Cm^RKm^R phenotype and verified by PCR and sequencing.

Table S1. Bacterial strains or plasmids used in this study.

Strain or Plasmid	Genotype, serotype or description	Reference or Source
C. jejuni		
81-176	Wild type *C. jejuni* strain isolated from a diarrheic patient	(Korlath *et al.*, 1985)
Δ*pgp1*	81-176 *pgp1*::*aphA3*; Km^R	(Frirdich *et al.*, 2012)
Δ*pgp2*	81-176 Δ*pgp2*	(Frirdich *et al.*, 2014)
Δ*1105*	81-176 *1105*::*aphA3*; Km^R	This study
11168-O (original)	The original clinical isolate of human origin isolated in the UK in 1977 by M. Skirrow. Minimally passaged.	(Gaynor *et al.*, 2004, Skirrow, 1977)
11168-GS (genome sequenced)	The genome sequenced NCTC 11168. Subculture history unknown. Provided by B. Wren.	(Gaynor *et al.*, 2004)
11168-M (microaerophilically passaged)	11168-O strain microaerophilically passaged 13 times at 37 °C on sheep blood agar plates containing actidione, vancomycin, trimethoprim and polymyxin B	(Gaynor *et al.*, 2004)
dim111	81-176 *thiC*::*solo (aphA3)* Tn; Km^R	This study
dim118	81-176 *pTET0039*::*solo (aphA3)* Tn; Km^R	This study
dim120	81-176 *pVir0003::solo (aphA3)* Tn; Km^R	This study
dim122	81-176 *0307*::*solo (aphA3)* Tn; Km^R	This study
dim128	81-176 *thiC*::*solo (aphA3)* Tn; Km^R	This study
dim129	81-176 *peb3*::*solo (aphA3)* Tn; Km^R	This study
dim132	81-176 *0206/7*::*solo (aphA3)* Tn; Km^R	This study
dim133	81-176 *0307*::*solo (aphA3)* Tn; Km^R	This study
dim111+pgp1-1345	*dim111 rrn::pgp1-1345* (from pEF38R); Km^R Cm^R	This study
dim118+pgp1-1345	*dim118 rrn::pgp1-1345* (from pEF38R); Km^R Cm^R	This study
dim120+pgp1-1345	*dim120 rrn::pgp1-1345* (from pEF38R); Km^R Cm^R	This study
dim122+pgp1-1345	*dim122 rrn::pgp1-1345* (from pEF38R); Km^R Cm^R	This study
dim128+pgp1-1345	*dim128 rrn::pgp1-1345* (from pEF38R); Km^R Cm^R	This study
dim129+pgp1-1345	*dim129 rrn::pgp1-1345* (from pEF38R); Km^R Cm^R	This study
dim132+pgp1-1345	*dim132 rrn::pgp1-1345* (from pEF38R); Km^R Cm^R	This study
dim133+pgp1-1345	*dim133rrn::pgp1-1345* (from pEF38R); Km^R Cm^R	This study
dim111+pgp1-1348	*dim111 rrn::pgp1-1348* (from pEF35R); Km^R Cm^R	This study
dim118+pgp1-1348	*dim118 rrn::pgp1-1348* (from pEF35R); Km^R Cm^R	This study
dim120+pgp1-1348	*dim120 rrn::pgp1-1348* (from pEF35R); Km^R Cm^R	This study
dim122+pgp1-1348	*dim122 rrn::pgp1-1348* (from pEF35R); Km^R Cm^R	This study
dim128+pgp1-1348	*dim128 rrn::pgp1-1348* (from pEF35R); Km^R Cm^R	This study
dim129+pgp1-1348	*dim129 rrn::pgp1-1348* (from pEF35R); Km^R Cm^R	This study
dim132+pgp1-1348	*dim132 rrn::pgp1-1348* (from pEF35R); Km^R Cm^R	This study
dim133+pgp1-1348	*dim133 rrn::pgp1-1348* (from pEF35R); Km^R Cm^R	This study
dim111+1105	*dim111 rrn::1105* (from pRRC-*1105*); Km^R Cm^R	This study

dim128+1105	*dim128 rrn::1105* (from pRRC-*1105*); Km^R Cm^R	This study
81-176 12-1	Straight variant of 81-176 isolated by passage on CFW	This study
81-176 12-9	Straight variant of 81-176 isolated by passage on CFW	This study
81-176 12-10	Helical variant of 81-176 isolated by passage on CFW	This study
81-176 25-1	Straight variant of 81-176 isolated by passage on CFW	This study
81-176 25-6	Helical variant of 81-176 isolated by passage on CFW	This study
81-176 33-1	Straight variant of 81-176 isolated by passage on CFW	This study
81-176 33-2	Helical variant of 81-176 isolated by passage on CFW	This study
81-176 12-1+*pgp1-1345*	81-176 12-1 *rrn::pgp1-1345* (from pEF38R); Cm^R	This study
81-176 12-9+*pgp1-1345*	81-176 12-9 *rrn::pgp1-1345* (from pEF38R); Cm^R	This study
81-176 25-1+*pgp2*	81-176 25-1 *rrn::pgp2* (from pJV4); Cm^R	This study
81-176 33-1+*pgp1-1345*	81-176 33-1 *rrn::pgp1-1345* (from pEF38R); Cm^R	This study
Δ*pgp1+pgp1-1345*	81-176 Δ*pgp1 rrn::pgp1-1345* (from pEF38R)	This study
Δ*pgp1*+12-1 *pgp1-1345*	81-176 Δ*pgp1 rrn::* 12-1 *pgp1-1345* (from pEF82R)	This study
Δ*pgp1*+33-1 *pgp1-1345*	81-176 Δ*pgp1 rrn::* 33-1 *pgp1-1345* (from pEF80R)	This study
Δ*pgp1+pgp1-1345*$_{11168\text{-}GS}$	81-176 Δ*pgp1 rrn::* 11168-GS *pgp1-1345* (from pEF83R)	This study
Δ*pgp1+pgp1-1345*$_{11168\text{-}O}$	81-176 Δ*pgp1 rrn::* 11168-O *pgp1-1345* (from pEF84R)	This study
Δ*pgp2+pgp2* (Δ*pgp2*c)	81-176 Δ*pgp2 rrn::pgp2* (from pEF81)	(Frirdich *et al.*, 2014)
Δ*pgp2*+25-1 *pgp2*	81-176 Δ*pgp2 rrn::* 25-1 *pgp2* (from pJV4)	This study
11168-O-Δ*pgp1*	11168-O *pgp1*::*aphA3*; Km^R	This study
11168-GS+*pgp1-1345*$_{11168\text{-}O}$	11168-GS *rrn::* 11168-O *pgp1-1345* (from pEF84R); Cm^R	This study
11168-GS-Δ*mreB+pgp1-1345*$_{11168\text{-}O}$ *mreB*$_{11168\text{-}O}$	11168-GS *rrn::* 11168-O *pgp1-1345* (from pEF84R) 11168-O *mreB* (from pEF90); $Km^R Cm^R Apr^R$	This study
11168-O-Δ*mreB*+ *mreB*$_{11168\text{-}GS}$	11168-O *mreB*::*aphA3 rrn::* 11168-GS *mreB* (from pEF89); $Km^R Apr^R$	This study
11168-GS+*pgp1-1345*$_{11168\text{-}O}$ + *0455*$_{11168\text{-}O}$	11168-GS *rrn::* 11168-O *pgp1-1345* (from pEF84R) 11168-O *0455* (from pEF91); $Cm^R Km^R$	This study
11168-GS+*pgp1-1345*$_{11168\text{-}O}$ + *0796*$_{11168\text{-}O}$	11168-GS *rrn::* 11168-O *pgp1-1345* (from pEF84R) 11168-O *0796* (from pEF92); $Cm^R Km^R$	This study
11168-GS+*pgp1-1345*$_{11168\text{-}O}$ + *1305-1306*$_{11168\text{-}O}$	11168-GS *rrn::* 11168-O *pgp1-1345* (from pEF84R) 11168-O *1305-1306* (from pEF93); $Cm^R Km^R$	This study
11168-M+*pgp1-1345*$_{81\text{-}176}$	11168-M *rrn::* 81-176 *pgp1-1345* (from pEF38R); Cm^R	This study
11168-M+*pgp1-1345*$_{11168\text{-}O}$	11168-M *rrn::* 11168-O *pgp1-1345* (from pEF84R); Cm^R	This study
pRRC	*C. jejuni* rRNA spacer integration vector; Cm^R	(Karlyshev & Wren, 2005)

pRRA	*C. jejuni* rRNA spacer integration vector; Apr^R	(Cameron & Gaynor, 2014)
pGEM-T	PCR cloning vector; Ap^R	Promega
pUC18-K2	Source of non-polar *aphA3* cassette; Ap^RKm^R	(Menard *et al.*, 1993)
pEF35R	pRRC-1344-1348 coding for 81-76 *pgp1* and 3816 bp upstream of *pgp1* in the reverse orientation as the *cat* cassette; Cm^R	(Frirdich *et al.*, 2012)
pEF38R	pRRC-1344-1345 coding for 81-176 *pgp1* and 1310 bp upstream of *pgp1*; in the reverse orientation as the *cat* cassette; Cm^R	This study
pEF80R	pRRC-1344-1345 coding for 81-76 33-1 *pgp1* and 1310 bp upstream of *pgp1*; in the reverse orientation as the *cat* cassette; Cm^R	This study
pEF81	pRRC containing 81-176 25-1 *pgp2* cloned with 196 bp of upstream sequence in the same orientation as the *cat* cassette; Cm^R	This study
pEF82R	pRRC-1344-1345 coding for 81-176 12-1 *pgp1* and 1310 bp upstream of *pgp1*; in the reverse orientation as the *cat* cassette; Cm^R	This study
pEF83R	pRRC-1344-1345 coding for 11168-GS *pgp1* and 1310 bp upstream of *pgp1*; in the reverse orientation as the *aphA3* cassette; Cm^R	This study
pEF84R	pRRC-1344-1345 coding for 11168-O *pgp1* and 1310 bp upstream of *pgp1*; in the reverse orientation as the *cat* cassette; Cm^R	This study
pGEMT-*1105*	pGEMT ligated to *1105* amplified with 1105-L1 and 1105-R1; Ap^R	This study
pGEMT-*1105*Km	pGEMT-*pgp1* with the *1105* gene disrupted with the *aphA3* cassette; Ap^RKm^R	This study
pJV4	pRRC containing 81-176 *pgp2* cloned with 196 bp of upstream sequence in the same orientation as the *cat* cassette; Cm^R	(Frirdich *et al.*, 2014)
pEF89	pRRA containing 11168-GS *mreB* cloned with 68 bp of upstream sequence in the same orientation as the *aac(3)IV* cassette; Apr^R	This study
pEF90	pRRA containing 11168-O *mreB* cloned with 68 bp of upstream sequence in the same orientation as the *aac(3)IV* cassette; Apr^R	This study
pEF91	pRRK containing 11168-O *0455* cloned with 18 bp of upstream sequence in the same	This study
pEF92	pRRK containing 11168-O *0796* cloned with 85 bp of upstream sequence in the same orientation as the *aphA3* cassette; Km^R	This study
pEF93	pRRK containing 11168-O *1306-1305* cloned with 262 bp of sequence upstream of 1306 in the same orientation as the *aphA3* cassette; Km^R	This study
pEF100	pGEMT ligated to *mreB* (81-176) amplified with	This study

	mreB-1 and mreB-2; Ap^R	
pEF101	pEF100 with the *mreB* gene disrupted with the *aphA3* cassette; Ap^RKm^R	This study

Table S2. Primers used in this study. Restriction sites are underlined and nucleotides differing from the wild type sequenced and changed in the primers are indicated by lower case letters.

Primer	Sequence 5′ to 3′	Restriction Site	Reference
ak233	GCAAGAGTTTTGCTTATGTTAGCAC		(Karlyshev & Wren, 2005)
ak234	GAAATGGGCAGAGTGTATTCTCCG		(Karlyshev & Wren, 2005)
ak235	GTGCGGATAATGTTGTTTCTG		(Karlyshev & Wren, 2005)
cat-2	GTTTTTTGGATGAATTACAAGA		(Frirdich *et al.*, 2012)
0915-JV-2	atacaattgCTTCTTCTTCACTCTTAACAGC	*MfeI*	(Frirdich *et al.*, 2014)
0915-JV-6	gcgactagtTGCCTAAAAAAGACATTCCTATAAA	*SpeI*	(Frirdich *et al.*, 2014)
1105-L1	CTGCTAAGGCTATGCTTGAT		This study
1105-R1	TGAGCGAGTAAATCTGCTTG		This study
1105-IL1	acgggtaccTTCTACTTCTAAGCCCAAAGC	*KpnI*	This study
1105-IR1	gcgtctagaGGCAGTTGATAGGTTATAGCG	*XbaI*	This study
1105-L2	gcgtctagaCTGCTAAGGCTATGCTTGAT	*XbaI*	This study
1105-R2	gcacaattgTACCACTTAACTCTCCATCAAC	*MfeI*	This study
1346-1	GCTTGATTTGTactAGTGTTATTGGAAC	*SpeI*	This study
1344-15	TGAAGTCTTGCAaCtAGTTCAGGTAC	*SpeI*	(Frirdich *et al.*, 2012)
mreB-1	TTGATGAGATTGATAAAATTGC		This study
mreB-2	AAGGCTTTACTTCTACACTTTGA		This study
mreB-9	CTATACCggTAcCTTTTACCAA	*KpnI*	This study
mreB-10	CTTTAGAGGAtATcTCATTATTGC	*EcoRV*	This study
mreB-FWDC	gcgtctagaCCAAAGAAGTTGTTAAAGACAATGC	*XbaI*	This study
mreB-REVC	atacaattgATTTTGCTTGATAAGTCCTCCATA	*MfeI*	This study
0455-1	ctagtctagaGATGCGAAAGGAGGTTAAATGACT	*XbaI*	This study
0455-2	gcgcaattgGCTCTAAACTTTTGTTGTTTTCGCTT	*MfeI*	This study
0796-1	ctagcttctagaGTAATGCTTAGTATGAAAGAATTTGAACGC	*XbaI*	This study
0796-2	gcgcaattgAAGAATACCATTGTAAAGCACTCATAAGATA	*MfeI*	This study
1305/6-1	ctagcttctagaGCAAGATGATTAAAACAGGAGCTTG	*XbaI*	This study
1305/6-2	gcgcaattgGATATGCTACTTGATGATATAGATGAGTGg	*MfeI*	This study
1344-SYBR-F2	CGC TGT TAT CGC AGA AGG A		This study
1344-SYBR-R2	GGA ACG GTG GTT TCA TTG C		This study

Table S3. Genomic changes relative to the *C. jejuni* 81-176 reference sequence in GenBank (NC_008787) of our laboratory strain of *C. jejuni* 81-176 and derivatives of 81-176 passaged on CFW: transposon mutants with a slight loss of helical morphology, *dim111* and *dim128*, and variants of 81-176 with helical (12-10, 25-6 and 33-2) and straight (12-1, 25-1 and 33-1) morphologies.

									Sample (morphology)[1]								
	Locus	Putative function	Nucleotide position	Change	Codon change	Amino acid change	Polymorphism	Protein effect	81-176 (helical)	*dim111* (loss of curvature)	*dim128* (loss of curvature)	12-1 (straight)	12-10 (helical)	25-1 (straight)	25-6 (helical)	33-1 (straight)	33-2 (helical)
1		Intergenic between hypothetical proteins CJJ81176_0082 and CJJ81176_0083	76,411	(C)9 -> (C)10			Insertion (tandem repeat)		16.80%	21.10%						20.90%	
2	CJJ81176_0086	anion transporter	78,365	(G)9 -> (G)10			Insertion (tandem repeat)	Frame Shift			80.50%	76.80%	86.40%		80.00%		
3	CJJ81176_0113	lctP CDS	99,044	(T)7 -> (T)8			Insertion (tandem repeat)	Frame Shift	89.40%	89.70%	95.20%	90.90%	92.80%	92.90%	93.20%	91.40%	90.40%
4	CJJ81176_0206	conserved hypothetical protein CDS	178,080	C -> G	ACC -> ACG		SNP (transversion)	None		21.50%							
			178,090	(G)9 -> (G)10			Insertion (tandem repeat)	Frame Shift	55.40%	50.90%	56.30%	71.60%	71.90%	76.60%	66.30%	68.50%	72.30%
5	CJJ81176_0227	purF CDS	197,289	C -> T	AGC -> AAC	S -> N	SNP (transition)	Substitution	94.90%	98.70%	100.00%	100.00%	100.00%	100.00%	100.00%	99.00%	100.00%
6	CJJ81176_0236	oligopeptide transporter, OPT family CDS	205,081	G -> T	TTG -> TTT	L -> F	SNP (transversion)	Substitution				19.20%		17.40%			
7	CJJ81176_0415	pyk CDS, pyruvate kinase	362,392	G -> T	AGC -> AGA	S -> R	SNP (transversion)	Substitution			17.20%	19.70%					
8	CJJ81176_0477	DNA polymerase III subunit epsilon CDS, 3'-5' exonuclease of DNA polymerase III	419,846	TATAGAA -> GGGGACT	ACT,ATA,GAA -> ACG,GGG,ACT	TIE -> TGT	Substitution	Substitution		29.20%							
			419,854	T -> A	TTT -> TAT	F -> Y	SNP (transversion)	Substitution		34.80%	22.60%						
			419,856	GCA -> CAG	GCA -> CAG	A -> Q	Substitution	Substitution		48.10%	33.30%						
			419,860	A -> C	CAA -> CCA	Q -> P	SNP (transversion)	Substitution		48.30%	41.90%						
			419,862	TGT -> ACC	TGT -> ACC	C -> T	Substitution	Substitution		56.7% -> 58.6%	50.00%						
			419,868	-A			Deletion	Frame Shift		59.40%	50.00%						
			419,871	GA -> AC	GAA -> ACA	E -> T	Substitution	Substitution		25.70%	41.20%						
			419,874	A -> G	AGT -> GGT	S -> G	SNP (transition)	Substitution		23.50%	38.90%						
			419,877	CC -> TG			Substitution	Truncation		15.60%							
			419,877	CCAAAA -> TGGCTG	CCA,AAA -> TGG,CTG	PK -> WL	Substitution	Substitution			15.8% -> 31.6%						
9	CJJ81176_0505	rplK CDS, binds directly to 23S ribosomal RNA	438,497	T -> G	GGT -> GGG		SNP (transversion)	None					17.30%	21.00%	17.70%	17.10%	
10		Intergenic between hypothetical proteins CJJ81176_0589 and CJJ81176_0590	524,734	(G)10 -> (G)11			Insertion (tandem repeat)		58.50%	59.60%	67.80%	77.30%	27.80%/ 61.10%	63.90%	60.70%		
11	CJJ81176_0614	ligA CDS, NAD-dependent DNA ligase LigA, this protein catalyzes the formation of phosphodiester linkages between 5'-phosphoryl and 3'-hydroxyl groups in double-stranded DNA using NAD as a coenzyme and as the energy source for the reaction; essential for DNA replication and repair of damaged DNA; similar to ligase LigB	543,209	A -> T	TTA -> TTT	L -> F	SNP (transversion)	Substitution	16.30%								
12	CJJ81176_0708	invasion phenotype protein CDS	634,752	(G)9 -> (G)8			Deletion (tandem repeat)	Frame Shift		56.40%	75.40%					88.60%	84.60%
13	CJJ81176_0758*	conserved hypothetical protein CDS	685,347	C -> G	CGG -> GGG	R -> G	SNP (transversion)	Substitution						35.60%			
			685,356	(G)9 -> (G)8			Deletion (tandem repeat)	Frame Shift	69.50%	73.10%	61.50%	82.20%	71.10%			68.80%	80.00%
			685,357	(G)9 -> (G)10			Insertion (tandem repeat)	Frame Shift						20.00%			
			685,357	A -> G	GAT -> GGT		SNP (transition)	Substitution						32.10%			
14		Intergenic, between CJJ81176_0765 and CJJ81176_1724 (rrsC, 16S	692,440	(C)11 -> (C)10			Deletion (tandem repeat)		72.60%	77.40%	80.30%	82.20%	88.10%	16.40%	82.00%	76.70%	80.30%

		ribosomal RNA)															
15	CJJ81176_0915	0915/conserved hypothetical protein CDS	846,347	C -> A	GGT -> TGT	G -> C	SNP (transversion)	Substitution						100.00%			
16		Intergenic between CJJ81176_0920 (CysK, cysteine synthase A) and CJJ81176_1731 (Hup, DNA-binding protein HU)	849,698	+T			Insertion		95.30%	93.20%	90.70%	98.50%	94.60%	95.20%	95.90%	92.40%	98.60%
			849,704	(T)2 -> (T)3			Insertion (tandem repeat)		98.80%	95.30%	95.00%	92.90%	96.00%	98.40%	98.60%	95.70%	97.30%
17	CJJ81176_0969	conserved hypothetical protein CDS	892,364	A -> C	TAT -> GAT	Y -> D	SNP (transversion)	Substitution						25.60%			
18		Intergenic between CJJ81176_1741 (putative periplasmic protein) and CJJ81176_1742 (hypothetical protein)	913,102	C -> A			SNP (transversion)						16.70%				
19	CJJ81176_1074	murC CDS, Catalyzes the formation of UDP-N-acetylmuramoyl-L-alanine from UDP-N-acetylmuramate and L-alanine in peptidoglycan synthesis	993,371	A -> C	GGT -> GGG		SNP (transversion)	None								15.40%	
20	CJJ81176_1103	mfd CDS	1,018,688	T -> C	GGA -> GGG		SNP (transition)	None	15.40%								
21	CJJ81176_1105	1105/peptidase, M23/M37 family CDS	1,021,639	-ACCT			Deletion	Frame Shift		95.1% -> 97.0%	98.0% -> 99.3%						
			1,021,802	T -> C	AGC -> GGC	S -> G	SNP (transition)	Substitution	100.00%	100.00%	100.00%	100.00%	100.00%	100.00%	100.00%	100.00%	100.00%
22	CJJ81176_1160	beta-1,4-N-acetylgalactosaminyltransferase CDS	1,079,106	(G)10 -> (G)9			Deletion (tandem repeat)	Frame Shift	85.70%	90.30%	88.10%	90.00%	93.20%	89.20%	76.80%	90.80%	88.40%
23	CJJ81176_1198	cyclopropane-fatty-acyl-phospholipid synthase, putative CDS	1,111,962	A -> C	GTT -> GGT	V -> G	SNP (transversion)	Substitution	16.10%		17.10%		21.20%				
24	CJJ81176_1226	glcD CDS, glycolate oxidase, subunit GlcD	1,144,423	A -> C	GTG -> GGG	V -> G	SNP (transversion)	Substitution									17.00%
25	CJJ81176_1279	recR CDS, recombination protein RecR, involved in a recombinational process of DNA repair, independent of the recBC complex	1,195,414	G -> A	GGA -> GAA	G -> E	SNP (transition)	Substitution	34.60%	27.90%	29.20%	34.30%	28.30%	38.70%	36.70%	41.10%	27.10%
26	CJJ81176_1290	pyrH CDS, uridylate kinase, Catalyzes the phosphorylation of UMP to UDP	1,209,229	A -> C	GGT -> GGG		SNP (transversion)	None									16.90%
27		Intergenic between CJJ81176_1321 (hypothetical protein) and CJJ81176_1322 (amino acid adenylation domain-containing protein)	1,236,763	(C)9 -> (C)10			Insertion (tandem repeat)		44.30%	46.90%	57.10%	16.90%	68.60%		56.70%	58.30%	67.40%
28	CJJ81176_1327	conserved hypothetical protein CDS	1,241,165	(C)9 -> (C)10			Insertion (tandem repeat)	Frame Shift	15.20%			55.30%	65.80%				18.80%
29	CJJ81176_1338	flagellin B, FlaB; structural flagella protein; in Helicobacter the flagella are composed of flagellin A and flagellin B; the amounts of each seem to be controlled by environmental conditions	1,253,555	T -> C	GAT -> GGT	D -> G	SNP (transition)	Substitution						15.50%			
30	CJJ81176_1341	hypothetical protein CDS	1,259,535	(C)9 -> (C)8			Deletion (tandem repeat)	Frame Shift						75.00%	74.60%	80.30%	
			1,259,536	(C)9 -> (C)10			Insertion (tandem repeat)	Frame Shift									69.90%
31	CJJ81176_1344	1344/conserved hypothetical protein CDS	1,261,811	G -> A	TCT -> TTT	S -> F	SNP (transition)	Substitution								100.00%	
			1,262,493	C -> A	GTC -> TTC	V -> F	SNP (transversion)	Substitution				98.90%					
32	CJJ81176_1354	enterochelin ABC transporter, periplasmic enterochelin-binding protein, authentic frameshift CDS	1,271,069	A -> G	AAT -> GAT	N -> D	SNP (transition)	Substitution	100.00%	100.00%	100.00%	100.00%	100.00%	100.00%	100.00%	100.00%	100.00%
33	CJJ81176_1381	selB CDS, selenocysteine-specific elongation factor	1,306,353	T -> G	GGT -> GGG		SNP (transversion)	None		15.80%							16.20%
34	CJJ81176_1397	feoB CDS, ferrous iron transport protein B	1,320,233	A -> C	TAT -> TCT	Y -> S	SNP (transversion)	Substitution		16.20%	20.40%						
			1,320,243	A -> G	CCA -> CCG		SNP (transition)	None		24.70%					20.50%		
			1,320,243	AA -> GT	CCA,AAT -> CCG,TAT	PN -> PY	Substitution	Substitution									15.0% -> 19.5%
			1,320,243	AAA -> GTC	CCA,AAT -> CCG,TCT	PN -> PS	Substitution	Substitution			23.2% -> 27.4%						

			1,320,244	AA -> TC	AAT -> TCT	N -> S	Substitution	Substitution		21.1% -> 21.4%							
			1,320,248	GG -> CT	TGG -> TCT	W -> S	Substitution	Substitution			21.00%						
			1,320,248	GGTA -> CTGC	TGG,TAT -> TCT,GCT	WY -> SA	Substitution	Substitution		19.7% -> 20.3%							
			1,320,250	TA -> GC	TAT -> GCT	Y -> A	Substitution	Substitution			21.6% -> 21.8%						
			1,320,254	T -> G			SNP (transversion)	Truncation		16.20%	19.60%						
			1,320,262	T -> A	TTT -> ATT	F -> I	SNP (transversion)	Substitution		16.20%	16.20%						
35	CJJ81176_1420	putative glycosyltransferase CDS	1,343,539	(C)10 -> (C)9			Deletion (tandem repeat)	Frame Shift		88.90%	85.00%				78.10%	72.50%	70.00%
	CJJ81176_1421		1,343,539	(C)10 -> (C)9			Deletion (tandem repeat)	Frame Shift		88.90%	85.00%				78.10%	72.50%	70.00%
36	CJJ81176_1429	conserved hypothetical protein CDS	1,349,694	(C)10 -> (C)9			Deletion (tandem repeat)	Frame Shift			53.00%						
37	CJJ81176_1432	galactosyltransferase CDS	1,352,287	(C)9 -> (C)10			Insertion (tandem repeat)	Frame Shift	67.20%	18.60%	38.80%	72.00%	74.40%	40.30%		79.00%	
			1,352,287	(CC)4 -> (CC)5			Insertion (tandem repeat)	Frame Shift						29.90%			
38	CJJ81176_1435	putative sugar transferase CDS	1,358,372	(C)9 -> (C)8			Deletion (tandem repeat)	Frame Shift		64.60%	53.20%						
39		Intergenic between CJJ81176_1448 (PrfB, peptide chain release factor 2 recognizing the termination signals UGA and UAA during protein translation) and CJJ81176_1449c (hypothetical protein)	1,371,836	+GAA			Insertion					15.20%		18.90%			19.20%
			1,371,853	T -> C			SNP (transition)		18.30%	23.30%	24.60%	26.40%	34.60%	29.80%	21.90%	23.00%	30.40%
			1,371,890	G -> A			SNP (transition)		21.40%	35.00%	34.40%	27.40%	36.70%	32.80%	27.50%	32.90%	33.80%
			1,371,914	C -> T			SNP (transition)		20.30%	41.90%	38.90%	25.80%	38.40%	32.40%	29.70%	33.80%	35.20%
			1,371,927	A -> G			SNP (transition)		43.70%	38.90%	20.00%	25.80%	37.70%	32.60%	28.40%	34.90%	36.40%
40	CJJ81176_1449	conserved domain protein CDS	1,371,968	(T)5 -> (T)6			Insertion (tandem repeat)	Frame Shift	18.90%	47.10%	44.30%	26.80%	38.40%	32.40%	31.00%	36.20%	37.90%
			1,372,021	CT -> TC	TTA,GAA -> TTG,AAA	LE -> LK	Substitution	Substitution	31.70%	51.90%	50.3% -> 50.4%	38.90%	41.50%	44.8% -> 45.5%	36.60%	44.00%	48.60%
			1,372,043	A -> G	ATT -> ATC		SNP (transition)	None	37.10%	51.50%	51.80%	42.10%	41.50%	45.90%	41.10%	47.40%	46.90%
			1,372,049	G -> A	GGC -> GGT		SNP (transition)	None	35.20%	50.70%	52.20%	42.40%	39.20%	45.80%	43.40%	46.60%	46.10%
			1,372,060	TG -> CT	AAC,AAT -> AAA,GAT	NN -> KD	Substitution	Substitution	33.90%	51.5% -> 51.7%	51.9% -> 52.4%	40.5% -> 41.4%	38.70%	46.00%	42.8% - > 43.0%	44.7% -> 44.8%	49.00%
			1,372,078	T -> C	ACA -> GCA	T -> A	SNP (transition)	Substitution	35.20%	51.00%	52.70%	41.20%	40.50%	45.60%	41.80%	43.50%	49.70%
			1,372,103	T -> C	AAA -> AAG		SNP (transition)	None	37.10%	51.30%	53.00%	46.70%	47.20%	50.00%	44.40%	47.50%	51.80%
			1,372,108	G -> A	CAT -> TAT	H -> Y	SNP (transition)	Substitution	37.30%	50.60%	53.20%	46.30%	46.40%	50.60%	44.70%	47.00%	50.90%
			1,372,120	T -> G	AAA -> CAA	K -> Q	SNP (transversion)	Substitution	37.50%	50.60%	54.70%	47.50%	46.80%	52.10%	45.30%	47.00%	50.30%
		Intergenic between CJJ81176_1449 (hypothetical protein) and CJJ81176_1450 TruD (catalyzes the modification of U13 in tRNA(Glu))	1,372,263	-ATT			Deletion			24.5% -> 25.2%	22.6% -> 22.9%		16.3% -> 16.9%	24.30%		20.00%	23.60%
			1,372,269	G -> A			SNP (transition)			25.00%	22.50%		16.30%	23.70%		20.10%	24.20%
			1,372,276	G -> T			SNP (transversion)			21.50%	19.00%			21.10%			18.20%
			1,372,280	T -> A			SNP (transversion)			21.40%	17.70%			20.70%			18.20%
			1,372,299	C -> T			SNP (transition)			16.70%							
41	CJJ81176_1510	molybdopterin converting factor, subunit 2 CDS	1,431,738	G -> T	GCA -> TCA	A -> S	SNP (transversion)	Substitution					17.80%				
								Total changes	32	57	53	34	32	40	31	36	39

[1]The percentages shown represent the variant frequency. A blank cell indicates no change in that strain in comparison to the published sequence.

Table S4. Muropeptide composition of *C. jejuni* wild-type 81-176, 25-1, 25-6, 33-1, 33-2, 11168-O, 11168-GS and 11168-O-Δ*pgp1* mutant strain.

		In *C. jejuni* strains								
		**81-176**	**81-176 25-1**	**81-176 25-6**	**81-176 33-1**	**81-176 33-2**	**11168-O**	**11168-GS**	**11168-O**	**11168-O Δ*pgp1***
	Date analyzed	08/20 13	08/2013	08/2013	08/2013	08/2013	01/2013	01/2013	08/2013	08/2013
**Peak number[1]**	**Muropeptide[2]**	**% Peak area**								
1	Tri	8.75	0.00	6.31	26.16	6.53	9.49	28.55	7.00	31.04
2	TetraGly4	0.41	0.44	0.46	0.29	0.41	0.62	0.59	0.57	0.37
3	Tetra	16.06	27.28	15.72	3.41	14.93	15.49	1.51	16.24	1.34
4	PentaGly5	0.82	0.92	0.70	0.41	0.84	1.01	0.67	0.89	0.57
5	Di	12.96	7.58	13.22	6.76	12.42	12.30	3.05	14.98	4.20
6	Tri-Ac	1.01	0.49	1.40	1.98	1.12	0.63	6.06	0.94	0.59
7	Tetra-Ac	0.61	2.53	1.10	0.00	0.70	0.35	0.00	0.39	0.00
8	Di-Ac	0.24	0.00	0.90	0.31	0.91	0.28	0.00	0.46	0.00
9	TetraTri	10.53	0.00	8.54	21.47	9.10	11.66	21.17	10.17	28.87
10	TetraPentaGly5	0.77	0.71	0.63	0.47	0.88	0.93	0.32	0.88	1.00
11	TetraTetra	21.40	29.86	21.30	10.26	21.56	21.66	5.63	21.24	7.93
12	TetraTri-Ac	1.47	0.00	1.89	2.78	1.66	1.21	7.23	1.34	0.92
13	TetraTetraTri	0.96	0.00	0.70	2.00	0.90	1.15	1.73	0.96	3.31
14	TetraTetra-Ac	1.65	4.68	2.55	0.74	1.96	0.29	0.00	1.01	0.00
15	TetraTetraTetra	2.54	3.11	2.46	1.41	2.90	4.10	1.67	2.68	1.04
16	TetraTriAnh I	1.10	0.00	0.30	2.14	1.06	0.87	2.12	1.06	2.58
17	TetraTriAnh II	2.36	0.00	1.01	5.93	2.32	2.57	6.23	2.54	6.87
18	TetraTetraAnh I	2.77	4.33	2.96	1.50	2.94	2.62	0.91	2.91	0.98
19	TetraTetraAnh II	5.20	7.51	5.52	2.44	5.51	4.98	1.27	5.36	1.53
20	TetraTetraTriAnh I	1.13	0.00	0.85	2.57	1.11	1.50	2.63	1.32	3.70
21	TetraTetraTetraAnh I	4.06	5.15	4.15	2.35	4.78	4.66	0.96	4.33	1.57
1 - 21	All known	96.80	94.59	92.67	95.38	94.54	98.37	92.70	97.27	98.41

[1] Peak numbers correspond to peaks labeled in the HPLC chromatogram shown in Fig. 5.

[2] Muropeptides are named according to Glauner, Holtje and Schwarz (Glauner *et al.*, 1988) and are depicted in Fig. 5E. Di, disaccharide dipeptide (disaccharide = β 1,4-linked N-acetylglucosamine-N-acetylmuramic acid); Tri, disaccharide tripeptide; Tetra, disaccharide tetrapeptide; Penta, disaccharide pentapeptide; Gly, glycine in position 5 of a peptide side chain; Ac, O-acetyl groups at the C-6 hydroxyl group of MurNAc; Anh, 1,6-anhydromuramic acid. Disaccharides are linked to form dimers or trimers by DD-crosslinks between amino acids 4 (D-Ala) and 3 (*meso*-DAP).

Table S5. Genomic changes relative to NCTC 11168-GS in Gen Bank (NC_002163; Parkhill *et al.*, 2000) in our laboratory stocks of the 11168-O (original) and 11168-GS (genome sequenced strain), and 11168-M strain (a 11168-O strain microaerophilically passaged 13 times).

									Sample (variant frequency)		
	**Locus**	**Putative function**	**Nucleotide position**	**Change**	**Codon change**	**Amino acid change**	**Polymorphism**	**Protein effect**	**11168-O**	**11168-GS**	**11168-M**
1	Cj0031	putative type IIS restriction/modification enzyme CDS	49,004	(G)10 -> (G)9			Deletion (tandem repeat)	Frame Shift	18.40%		63.40%
			49,005	(G)10 -> (G)9			Deletion (tandem repeat)	Frame Shift	32.00%	17.50%	79.50%
2	Cj0045c	putative iron-binding protein CDS<br>C9 and C10 (consensus) have little effect.	65,757	(C)10 -> (C)9			Deletion (tandem repeat)	Frame Shift		88.20%	
		C11 allows the translation of this CDS to extend by another 15aa, overlapping the start of Cj0044c	65,758	(C)10 -> (C)11			Insertion (tandem repeat)	Frame Shift	23.70%		35.20%
3	Cj0046	pseudogene (putative sodium:sulfate transmembrane transport protein) CDS	67,718	(G)11 -> (G)10			Deletion (tandem repeat)	Frame Shift	70.90%	15.20%	27.20%
4	Cj0088	dcuA gene, anaerobic C4-dicarboxylate transporter	97,940	C -> T	CCT -> TCT	P -> S	SNP (transition)				99.20%
5	Cj0183	putative serine/threonine protein phosphatase CDS, putative integral membrane protein with haemolysin domain	180,710	(AC)3 -> (AC)2			Deletion (tandem repeat)	Frame Shift		100.00%	94.60%
6	Cj0276	mreB CDS	253,191	A -> G	GAT -> GGT	D -> G	SNP (transition)	Substitution	98.80%[1]		100.00%
7	Cj0284c	cheA CDS, chemotaxis histidine kinase	262,345	A -> G	ATA -> ACA	I -> T	SNP (transition)	Substitution	100.00%[1]		100.00%
8	Cj0314	lysA CDS, diaminopimelate decarboxylase	284,954	T -> G	GGT -> GGG		SNP (transversion)	None	21.60%		
9	Cj0431	putative periplasmic ATP/GTP-binding protein CDS	393,542	T -> A			SNP (transversion)	Extension	100.00%[1]	98.70%	99.20%
10	Cj0437	sdhA CDS, succinate dehydrogenase flavoprotein subunit	405,774	A -> G	AAT -> GAT	N -> D	SNP (transition)	Substitution		100.00%	
11	Cj0447	putative NUDIX hydrolase family protein CDS,<br>NUDIX hydrolases = NUcleoside DIphosphate linked to some other moiety X<br>The family can be divided into a	414,589	+TATG			Insertion	Truncation			15.20%

		number of subgroups, of which MutT anti-mutagenic activity represents only one type; most of the rest hydrolyse diverse nucleoside diphosphate derivatives (including ADP-ribose, GDP- mannose, TDP-glucose, NADH, UDP-sugars, dNTP and NTP).									
12	Cj0455c	putative membrane protein CDS	420,550	A -> G			SNP (transition)	Extension	98.70%[1]		100.00%
13		Intergenic between Cj0564 (probable integral membrane protein) and Cj0565 (probable pseudogene)	527,388	(GG)6 -> (GG)5			Deletion (tandem repeat)		94.80%		
			527,388	(G)12 -> (G)11			Deletion (tandem repeat)			80.30%	69.60%
			527,389	(G)12 -> (G)11			Deletion (tandem repeat)			90.80%	89.10%
14	Cj0593c	putative integral membrane protein CDS	551,720	T -> C	AGC -> GGC	S -> G	SNP (transition)	Substitution		17.00%	
15	Cj0597	fba CDS, fructose-bisphosphate aldolase	554,844	T -> G	GGT -> GGG		SNP (transversion)	None			16.90%
16	Cj0617	hypothetical protein Cj0617 (617 family) CDS	577,595	(G)9 -> (G)10			Insertion (tandem repeat)	Frame Shift	33.30%	79.20%	
17	Cj0628	putative lipoprotein CDS (CapA autotransporter)	588,369	G -> T	GGG -> TGG	G -> W	SNP (transversion)	Substitution		15.50%	
			588,369	+TG			Insertion	Frame Shift		74.10%	
			588,379	(G)10 -> (G)11			Insertion (tandem repeat)	Frame Shift	71.40%		62.50%
			589,838	C -> T	TCT -> TTT	S -> F	SNP (transition)	Substitution		16.70%	
18	Cj0676	kdpA CDS, pseudogene (potassium-transporting ATPase A chain)	628,179	(G)9 -> (G)10			Insertion (tandem repeat)	Frame Shift			77.40%
19	Cj0685c	cipA CDS, Invasion protein CipA, possible sugar transferase	639,015	(G)9 -> (G)8			Deletion (tandem repeat)	Frame Shift	69.00%	86.80%	66.40%
20		Intergenic between Cj0742 (pseudogene, putative outer membrane protein) and 16s rRNA	695,953	(C)10 -> (C)11			Insertion (tandem repeat)		54.20%, 64.40%		27.80%, 53.30%
			695,953	A -> C			SNP (transversion)		20.30%		17.80%
			695,954	T -> C			SNP (transition)		17.20%		
21		5S rRNA	702,597	T -> G			SNP (transversion)				15.50%
22	Cj0807	putative oxidoreductase CDS	760,188	A -> G	AAA -> GAA	K -> E	SNP (transition)	Substitution	100.00%[1]	100.00%	100.00%
23	Cj0916c	conserved hypothetical protein Cj0916c CDS	852,193	T -> A	TTA -> TTT	L -> F	SNP (transversion)	Substitution			15.00%
24	Cj0935c	putative sodium:amino-acid symporter family protein CDS	871,680	C -> A	GTA -> TTA	V -> L	SNP (transversion)	Substitution	29.70%	21.20%	

			871,686	G -> A	CTT -> TTT	L -> F	SNP (transition)	Substitution	33.30%	20.60%	17.90%
			871,692	T -> A			SNP (transversion)	Truncation	20.00%		
25	Cj0945c	putative helicase CDS	885,046	+TTAAT			Insertion	Frame Shift	15.50%		80.60%
26	Cj1144c	hypothetical protein Cj1144c CDS (putative LOS glycosyltransferase)	1,079,748	(C)8 -> (C)9			Insertion (tandem repeat)	Frame Shift	85.20%		85.10%
27	Cj1213c	glcD CDS, putative glycolate oxidase subunit D	1,142,763	A -> C	GTG -> GGG	V -> G	SNP (transversion)	Substitution		25.60%	
28	Cj1259	porA CDS, major outer membrane protein	1,189,659	A -> G	GAA -> GGA	E -> G	SNP (transition)	Substitution	71.30%[1]		
29	Cj1262	racS CDS, two-component sensor (histidine kinase)	1,193,103	G -> A	ATG -> ATA	M -> I	SNP (transition)	Substitution		100.00%	
30	Cj1263	recR CDS, recombination protein	1,193,755	G -> A	GGA -> GAA	G -> E	SNP (transition)	Substitution	32.80%	22.40%	35.10%
31	Cj1286c	upp CDS, uracil phosphoribosyltransferase	1,218,203	A -> C	GGT -> GGG		SNP (transversion)	None	18.10%		
32	Cj1305c	hypothetical protein Cj1305c (617 family) CDS	1,234,931	(C)9 -> (C)10			Insertion (tandem repeat)	Frame Shift	64.90%		78.70%
33	Cj1306c	hypothetical protein Cj1306c (617 family) CDS	1,236,169	(C)9 -> (C)8			Deletion (tandem repeat)	Frame Shift		90.50%	
34	Cj1318	maf1 CDS	1,246,856	(G)11 -> (G)10			Deletion (tandem repeat)	Frame Shift	58.30%	89.60%	84.80%
35	Cj1320	putative aminotransferase (degT family) CDS	1,250,816	(G)10 -> (G)11			Insertion (tandem repeat)		48.30%		64.30%
36	Cj1325	putative methyltransferase CDS	1,253,678	(G)10 -> (G)9			Deletion (tandem repeat)	Frame Shift	82.50%		71.00%
37	Cj1335	maf4 CDS	1,263,684	(G)9 -> (G)10			Insertion (tandem repeat)	Frame Shift			16.00%
			1,263,684	T -> G	GGT -> GGG		SNP (transversion)	None			20.00%
38	Cj1342c	maf7 CDS	1,275,376	(C)9 -> (C)10			Insertion (tandem repeat)	Frame Shift	48.70%		56.10%
39	Cj1345c	Pgp1, DL-carboxypeptidase cleaving PG tripeptides to dipeptides	1,278,671	-T			Deletion	Frame Shift			100.00%
			1,278,694	-C			Deletion	Frame Shift		98.70%	
40	Cj1367c	putative nucleotidyltransferase CDS	1,304,325	(T)6 -> (T)7			Insertion (tandem repeat)	Frame Shift	44.10%		
41	Cj1379	selB CDS, putative triosephosphate isomerase	1,318,713	T -> G	GTT -> GGT	V -> G	SNP (transversion)	Substitution			17.30%
42	Cj1401c	tpiA CDS, probable triosephosphate isomerase	1,336,117	T -> C	AAT -> AGT	N -> S	SNP (transition)	Substitution		100.00%	
43		Intergenic between Cj1403c (GapA, glyceraldehyde 3-phosphate dehydrogenase) and Cj1404 (NadD, putative nicotinate-nucleotide	1,338,391	(A)7 -> (A)8			Insertion (tandem repeat)	Frame Shift	94.90%		93.30%

		adenylyltransferase)									
44	Cj1421c	putative sugar transferase CDS	1,355,967	(C)9 -> (C)10			Insertion (tandem repeat)	Frame Shift	26.40%		41.10%
45	Cj1422c	putative sugar transferase CDS	1,357,899	(C)9 -> (C)10			Insertion (tandem repeat)	Frame Shift	17.70%		33.70%
46	Cj1429c	hypothetical protein Cj1429c CDS	1,363,832	(C)10 -> (C)11			Insertion (tandem repeat)	Frame Shift			39.50%
47	Cj1437c	aminotransferase CDS	1,374,135	T -> C	ATA -> GTA	I -> V	SNP (transition)	Substitution	29.20%	15.80%	
48	Cj1470c	pesudogene (type II protein secretion system F protein) CDS	1,404,348	(T)5 -> (T)6			Insertion (tandem repeat)	Frame Shift	91.70%		91.00%
49	Cj1675	fliQ CDS probable flagellar biosynthetic protein	1,595,840	C -> T	CCT -> TCT	P -> S	SNP (transition)	Substitution			100.00%
								Total changes	36	25	41

[1] Change also detected in NCTC 11168-BN148 which is more similar to 11168-O in morphology, motility and invasion (Revez *et al.*, 2012).

Table S6. Genomic changes relative to NCTC 11168-GS in Gen Bank (NC_002163; Parkhill *et al.*, 2000) in our laboratory stocks of the 11168-O (original) and 11168-GS (genome sequenced strain), and 11168-M strain (a 11168-O strain microaerophilically passaged 13 times) that are either unique to our laboratory strain of 11168-GS or unique to 11168-O and 11168-M.[1]

									Sample (variant frequency)		
	**Locus**	**Putative function**	**Nucleotide position**	**Change**	**Codon change**	**Amino acid change**	**Polymorphism**	**Protein effect**	**11168-O**	**11168-GS**	**11168-M**
2	Cj0045c	putative iron-binding protein CDS C9 and C10 (consensus) have little effect.	65,757	(C)10 -> (C)9			Deletion (tandem repeat)	Frame Shift		88.20%	
		C11 allows the translation of this CDS to extend by another 15aa, overlapping the start of Cj0044c	65,758	(C)10 -> (C)11			Insertion (tandem repeat)	Frame Shift	23.70%		35.20%
**6**	**Cj0276**	**mreB CDS**	**253,191**	**A -> G**	**GAT -> GGT**	**D -> G**	**SNP (transition)**	**Substitution**	**98.80%[2]**		**100.00%**
7	Cj0284c	cheA CDS, chemotaxis histidine kinase	262,345	A -> G	ATA -> ACA	I -> T	SNP (transition)	Substitution	100.00%[2]		100.00%
10	Cj0437	sdhA CDS, succinate dehydrogenase flavoprotein subunit	405,774	A -> G	AAT -> GAT	N -> D	SNP (transition)	Substitution		100.00%	
**12**	**Cj0455c**	**putative membrane protein CDS**	**420,550**	**A -> G**			**SNP (transition)**	**Extension**	**98.70%[2]**		**100.00%**
17	Cj0628	putative lipoprotein CDS (CapA autotransporter)	588,369	G -> T	GGG -> TGG	G -> W	SNP (transversion)	Substitution		15.50%	
			588,369	+TG			Insertion	Frame Shift		74.10%	
			588,379	(G)10 -> (G)11			Insertion (tandem repeat)	Frame Shift	71.40%		62.50%
			589,838	C -> T	TCT -> TTT	S -> F	SNP (transition)	Substitution		16.70%	
20		Intergenic between Cj0742 (pseudogene, putative outer membrane protein) and 16s rRNA	695,953	(C)10 -> (C)11			Insertion (tandem repeat)		54.20%, 64.40%		27.80%, 53.30%
			695,953	A -> C			SNP (transversion)		20.30%		17.80%
			695,954	T -> C			SNP (transition)		17.20%		
26	Cj1144c	hypothetical protein Cj1144c CDS (putative LOS glycosyltransferase)	1,079,748	(C)8 -> (C)9			Insertion (tandem repeat)	Frame Shift	85.20%		85.10%
29	Cj1262	racS CDS, two-component sensor (histidine kinase)	1,193,103	G -> A	ATG -> ATA	M -> I	SNP (transition)	Substitution		100.00%	
**32**	**Cj1305c**	**hypothetical protein Cj1305c (617 family) CDS**	**1,234,931**	**(C)9 -> (C)10**			**Insertion (tandem repeat)**	**Frame Shift**	**64.90%**		**78.70%**
**33**	**Cj1306c**	**hypothetical protein Cj1306c (617 family) CDS**	**1,236,169**	**(C)9 -> (C)8**			**Deletion (tandem repeat)**	**Frame Shift**		**90.50%**	
35	Cj1320	putative aminotransferase (degT family) CDS	1,250,816	(G)10 -> (G)11			Insertion (tandem repeat)		48.30%		64.30%

36	Cj1325	putative methyltransferase CDS	1,253,678	(G)10 -> (G)9			Deletion (tandem repeat)	Frame Shift	82.50%		71.00%
38	Cj1342c	maf7 CDS	1,275,376	(C)9 -> (C)10			Insertion (tandem repeat)	Frame Shift	48.70%		56.10%
43		Intergenic between Cj1403c (GapA, glyceraldehyde 3-phosphate dehydrogenase) and Cj1404 (NadD, putative nicotinate-nucleotide adenylyltransferase)	1,338,391	(A)7 -> (A)8			Insertion (tandem repeat)	Frame Shift	94.90%		93.30%
**48**	**Cj1470c**	**pesudogene (type II protein secretion system F protein) CDS**	**1,404,348**	**(T)5 -> (T)6**			**Insertion (tandem repeat)**	**Frame Shift**	**91.70%**		**91.00%**
								Total changes			

[1] Changes that have not been highlighted by bold lettering are present in under 50% of the population or are in genes whose function would likely not affect morphology.

[2] Change also detected in NCTC 11168-BN148 which is more similar to 11168-O in morphology, motility and invasion (Revez *et al.*, 2012).

A

```
81-176          ATGAAAATTTTTTTAACAATTTTATTTTTTATAACAAGCATTTTTGCTTTAGATCTAGAC     60
33-1            ATGAAAATTTTTTTAACAATTTTATTTTTTATAACAAGCATTTTTGCTTTAGATCTAGAC     60
12-1/9          ATGAAAATTTTTTTAACAATTTTATTTTTTATAACAAGCATTTTTGCTTTAGATCTAGAC     60
dim118/129      ATGAAAATTTTTTTAACAATTTTATTTTTTATAACAAGCATTTTTGCTTTAGATCTAGAC     60
dim120          ATGAAAATTTTTTTAACAATTTTATTTTTTATAACAAGCATTTTTGCTTTAGATCTAGAC     60
dim122/133      ATGAAAATTTTTTTAACAATTTTATTTTTTATAACAAGCATTTTTGCTTTAGATCTAGAC     60
dim132          ATGAAAATTTTTTTAACAATTTTATTTTTTATAACAAGCATTTTTGCTTTAGATCTAGAC     60
11168-O         ATGAAATTTTTTTTAACAATTTTATTTTTTATAACAAGCATTTTTGCTTTAGAGCTAGAC     60
11168-GS        ATGAAATTTTTTTTAACAATTTTATTTTTTATAACAAGCATTTTTGCTTTAGAGCTAGAC     60
                ****** ********************************************** ******

81-176          TTTAGCGTAGGAGAAAATGGAAAAAGTCTAGATGATAATAATACAGTTTTAATTTTTGGT     120
33-1            TTTAGCGTAGGAGAAAATGGAAAAAGTCTAGATGATAATAATACAGTTTTAATTTTTGGT     120
12-1/9          TTTAGCGTAGGAGAAAATGGAAAAAGTCTAGATGATAATAATACAGTTTTAATTTTTGGT     120
dim118/129      TTTAGCGTAGGAGAAAATGGAAAAAGTCTAGATGATAATAATACAGTTTTAATTTTTGGT     120
dim120          TTTAGCGTAGGAGAAAATGGAAAAAGTCTAGATGATAATAATACAGTTTTAATTTTTGGT     120
dim122/133      TTTAGCGTAGGAGAAAATGGAAAAAGTCTAGATGATAATAATACAGTTTTAATTTTTGGT     120
dim132          TTTAGCGTAGGAGAAAATGGAAAAAGTCTAGATGATAATAATACAGTTTTAATTTTTGGT     120
11168-O         TTTAGCGTAGGAGAAAATGGAAAAAGTCTAGATGATAATAATACAGTTTTAATTTTTGGT     120
11168-GS        TTTAGCGTAGGAGAAAATGGAAAAAGTCTAGATGATAATAATACAGTTTTAATTTTTGGT     120
                ************************************************************

81-176          GGAATTCAAGGTGATGAGCCTGGTGGATTTCATGCGGCAAGTTTGCTTTTGAGTGATTAT     180
33-1            GGAATTCAAGGTGATGAGCCTGGTGGATTTCATGCGGCAAGTTTGCTTTTGAGTGATTAT     180
12-1/9          GGAATTCAAGGTGATGAGCCTGGTGGATTTCATGCGGCAAGTTTGCTTTTGAGTGATTAT     180
dim118/129      GGAATTCAAGGTGATGAGCCTGGTGGATTTCATGCGGCAAGTTTGCTTTTGAGTGATTAT     180
dim120          GGAATTCAAGGTGATGAGCCTGGTGGATTTCATGCGGCAAGTTTGCTTTTGAGTGATTAT     180
dim122/133      GGAATTCAAGGTGATGAGCCTGGTGGATTTCATGCGGCAAGTTTGCTTTTGAGTGATTAT     180
dim132          GGAATTCAAGGTGATGAGCCTGGTGGATTTCATGCGGCAAGTTTGCTTTTGAGTGATTAT     180
11168-O         GGAATTCAAGGCGATGAGCCTGGTGGATTTCATGCGGCAAGTTTACTTTTGAGTGATTAT     180
11168-GS        GGAATTCAAGGCGATGAGCCTGGTGGATTTCATGCG-CAAGTTTACTTTTGAGTGATTAT     179
                *********** ************************ ******* ***************

81-176          AATATCACCAAGGGTAAGATTATAGTGGCTCCAAATTTAGCTTTTGATAGCATTATCAAG     240
33-1            AATATCACCAAGGGTAAGATTATAGTGGCTCCAAATTTAGCTTTTGATAGCATTATCAAG     240
12-1/9          AATATCACCAAGGGTAAGATTATAGTGGCTCCAAATTTAGCTTTTGATAGCATTATCAAG     240
dim118/129      AATATCACCAAGGGTAAGATTATAGTGGCTCCAAATTTAGCTTTTGATAGCATTATCAAG     240
dim120          AATATCACCAAGGGTAAGATTATAGTGGCTCCAAATTTAGCTTTTGATAGCATTATCAAG     240
dim122/133      AATATCACCAAGGGTAAGATTATAGTGGCTCCAAATTTAGCTTTTGATAGCATTATCAAG     240
dim132          AATATCACCAAGGGTAAGATTATAGTGGCTCCAAATTTAGCTTTTGATAGCATTATCAAG     240
11168-O         AATATCACTAAGGGTAAGATTATAGTGGCTCCAAATTTAGCTTTTGATAGCATTATCAAG     240
11168-GS        AATATCACTAAGGGTAAGATTATAGTGGCTCCAAATTTAGCTTTTGATAGCATTATCAAG     239
                ******** ***************************************************

81-176          CGTTCGCGTGGGAATAATGGGGATTTAAACCGTAAATTTGCAAGCATTAGCCCAAAGGAT     300
33-1            CGTTCGCGTGGGAATAATGGGGATTTAAACCGTAAATTTGCAAGCATTAGCCCAAAGGAT     300
12-1/9          CGTTCGCGTGGGAATAATGGGGATTTAAACCGTAAATTTGCAAGCATTAGCCCAAAGGAT     300
dim118/129      CGTTCGCGTGGGAATAATGGGGATTTAAACCGTAAATTTGCAAGCATTAGCCCAAAGGAT     300
dim120          CGTTCGCGTGGGAATAATGGGGATTTAAACCGTAAATTTGCAAGCATTAGCCCAAAGGAT     300
dim122/133      CGTTCGCGTGGGAATAATGGGGATTTAAACCGTAAATTTGCAAGCATTAGCCCAAAGGAT     300
dim132          CGTTCGCGTGGGAATAATGGGGATTTAAACCGTAAATTTGCAAGCATTAGCCCAAAGGAT     300
11168-O         CGTTCTCGTGGGAATAATGGGGATTTAAACCGTAAATTTGCAAGCATTAGCCCAAAAGAT     300
11168-GS        CGTTCTCGTGGGAATAATGGGGATTTAAACCGTAAATTTGCAAGCATTAGCCCAAAAGAT     299
                ***** ************************************************** ***

81-176          CCTGATTATAAAACCGTGCAACGCATTAAAGAGCTTATTTACTTCCTGAAGTTAGTATG     360
33-1            CCTGATTATAAAACCGTGCAACGCATTAAAGAGCTTATTTACTTCCTGAAGTTAGTATG     360
12-1/9          CCTGATTATAAAACCGTGCAACGCATTAAAGAGCTTATTTACTTCCTGAAGTTAGTATG     360
dim118/129      CCTGATTATAAAACCGTGCAACGCATTAAAGAGCTTATTTACTTCCTGAAGTTAGTATG     360
dim120          CCTGATTATAAAACCGTGCAACGCATTAAAGAGCTTATTTACTTCCTGAAGTTAGTATG     360
dim122/133      CCTGATTATAAAACCGTGCAACGCATTAAAGAGCTTATTTACTTCCTGAAGTTAGTATG     360
dim132          CCTGATTATAAAACCGTGCAACGCATTAAAGAGCTTATTTACTTCCTGAAGTTAGTATG     360
11168-O         CCTGATTATAAAACCGTTCAACGCATTAAAGAGCTTATTTACTTCCTGAAGTTAGTATG     360
11168-GS        CCTGATTATAAAACCGTTCAACGCATTAAAGAGCTTATTTACTTCCTGAAGTTAGTATG     359
                ***************** ******************************************

81-176          GTGATCAATCTTCACGATGGTTGGGG-TTTTTATAAACCTACTTATATCGATGCGATGCA     419
33-1            GTGATCAATCTTCACGATGGTTGGGG-TTTTTATAAACCTACTTATATCGATGCGATGCA     419
```

```
12-1/9          GTGATCAATCTTCACGATGGTTGGGG-TTTTTATAAACCTACTTATATCGATGCGATGCA    419
dim118/129      GTGATCAATCTTCACGATGGTTGGGG-TTTTTATAAACCTACTTATATCGATGCGATGCA    419
dim120          GTGATCAATCTTCACGATGGTTGGGGGTTTTTATAAACCTACTTATATCGATGCGATGCA    420
dim122/133      GTGATCAATCTTCACGATGGTTGGGG-TTTTTATAAACCTACTTATATCGAT-CGATGCA    418
dim132          GTGATCAATCTTCACGATGGTTGGGG-TTTTTATAAACCTACTTATATCGATGCGATGCA    419
11168-O         GTGATCAATCTTCACGATGGTTGGGG-TTTTTATAAACCCACTTATATCGATGCGATGCA    419
11168-GS        GTGATCAATCTTCACGATGGTTGGGG-TTTTTATAAACCCACTTATATCGATGCGATGCA    418
                ************************** ************ ************ *******
81-176          AAATCCTAAGCGTTGGGGAAATTCAAGCGTGATTGACACAAGTGAAATCAATGCAAGCAA    479
33-1            AAATCCTAAGCGTTGGGGAAATTCAAGCGTGATTGACACAAGTGAAATCAATGCAAGCAA    479
12-1/9          AAATCCTAAGCGTTGGGGAAATTCAAGCGTGATTGACACAAGTGAAATCAATGCAAGCAA    479
dim118/129      AAATCCTAAGCGTTGGGGAAATTCAAGCGTGATTGACACAAGTGAAATCAATGCAAGCAA    479
dim120          AAATCCTAAGCGTTGGGGAAATTCAAGCGTGATTGACACAAGTGAAATCAATGCAAGCAA    480
dim122/133      AAATCCTAAGCGTTGGGGAAATTCAAGCGTGATTGACACAAGTGAAATCAATGCAAGCAA    478
dim132          AAATCCTAAGCGTTGGGGAAATTCAAGCGTGATTGACACAAGTGAAATCAATGCAAGCAA    479
11168-O         AAATCCTAAGCGTTGGGGAAATTCAAGCGTGATTGATACAAGCGAAATCAATGCAAGCAA    479
11168-GS        AAATCCTAAGCGTTGGGGAAATTCAAGCGTGATTGATACAAGCGAAATCAATGCAAGCAA    478
                ************************************ ***** *****************
81-176          ATACCCTGATCTTGAAAATATCGCCACTCAAACCGTAAATAGTGTCAATTCTTCACTTGC    539
33-1            ATACCCTGATCTTGAAAATATCGCCACTCAAACCGTAAATAGTGTCAATTCTTCACTTGC    539
12-1/9          ATACCCTGATCTTGAAAATATCGCCACTCAAACCGTAAATAGTTTCAATTCTTCACTTGC    539
dim118/129      ATACCCTGATCTTGAAAATATCGCCACTCAAACCGTAAATAGTGTCAATTCTTCACTTGC    539
dim120          ATACCCTGATCTTGAAAATATCGCCACTCAAACCGTAAATAGTGTCAATTCTTCACTTGC    540
dim122/133      ATACCCTGATCTTGAAAATATCGCCACTCAAACCGTAAATAGTGTCAATTCTTCACTTGC    538
dim132          ATACCCTGATCTTGAAAATATCGCCACTCAAACCGTAAATAGTGTCAATTCTTCACTTGC    539
11168-O         ATACCCTGATCTTGAAAATATCGCCACTCAAACCGTAAATAGTGTCAATTCTTCGCTTGC    539
11168-GS        ATACCCTGATCTTGAAAATATCGCCACTCAAACCGTAAATAGTGTCAATTCTTCGCTTGC    538
                ******************************************* ********** *****
81-176          TGATCCAAAGCATGCTTATCATCTGAAAAACACCAAAACCCAAGAGCTTGGCGATATGGA    599
33-1            TGATCCAAAGCATGCTTATCATCTGAAAAACACCAAAACCCAAGAGCTTGGCGATATGGA    599
12-1/9          TGATCCAAAGCATGCTTATCATCTGAAAAACACCAAAACCCAAGAGCTTGGCGATATGGA    599
dim118/129      TGATCCAAAGCATGCTTATCATCTGAAAAACACCAAAACCCAAGAGCTTGGCGATATGGA    599
dim120          TGATCCAAAGCATGCTTATCATCTGAAAAACACCAAAACCCAAGAGCTTGGCGATATGGA    600
dim122/133      TGATCCAAAGCATGCTTATCATCTGAAAAACACCAAAACCCAAGAGCTTGGCGATATGGA    598
dim132          TGATCCAAAGCATGCTTATCATCTGAAAAACACCAAAACCCAAGAGCTTGGCGATATGGA    599
11168-O         TGATCCAAAGCATGCTTATCATCTTAAAAACACCAAAACCCAAGAGCTTGGCGATACGGA    599
11168-GS        TGATCCAAAGCATGCTTATCATCTTAAAAACACCAAAACCCAAGAGCTTGGCGATACGGA    598
                ************************ ******************************* ***
81-176          AATGCTTAAAGCTTTGACTTATTTTGTGATTTCAAATCACAAAGCCGCTTTTGCAAATGA    659
33-1            AATGCTTAAAGCTTTGACTTATTTTGTGATTTCAAATCACAAAGCCGCTTTTGCAAATGA    659
12-1/9          AATGCTTAAAGCTTTGACTTATTTTGTGATTTCAAATCACAAAGCCGCTTTTGCAAATGA    659
dim118/129      AATGCTTAAAGCTTTGACTTATTTTGTGATTTCAAATCACAAAGCCGCTTTTGCAAATGA    659
dim120          AATGCTTAAAGCTTTGACTTATTTTGTGATTTCAAATCACAAAGCCGCTTTTGCAAATGA    660
dim122/133      AATGCTTAAAGCTTTGACTTATTTTGTGATTTCAAATCACAAAGCCGCTTTTGCAAATGA    658
dim132          AATGCTTAAAGCTTTGACTTATTTTGTGATTTCAAATCACAAAGCCGCTTTTGCAAATGA    659
11168-O         AATGCTTAAAGCTTTAACTTATTTTGTGATTTCAAATCACAAAGCCGCTTTTGCAAATGA    659
11168-GS        AATGCTTAAAGCTTTAACTTATTTTGTGATTTCAAATCACAAAGCCGCTTTTGCAAATGA    658
                *************** ********************************************
81-176          AGCGAGTAAAAATCTGCCTGTAAATTTAAGAGCTTATTATCATCTTTTGGCAATTGAAAA    719
33-1            AGCGAGTAAAAATCTGCCTGTAAATTTAAGAGCTTATTATCATCTTTTGGCAATTGAAAA    719
12-1/9          AGCGAGTAAAAATCTGCCTGTAAATTTAAGAGCTTATTATCATCTTTTGGCAATTGAAAA    719
dim118/129      AGCGAGTAAAAATCTGCCTGTAAATTTAAGAGCTTATTATCATCTTTTGGCAATTGAAAA    719
dim120          AGCGAGTAAAAATCTGCCTGTAAATTTAAGAGCTTATTATCATCTTTTGGCAATTGAAAA    720
dim122/133      AGCGAGTAAAAATCTGCCTGTAAATTTAAGAGCTTATTATCATCTTTTGGCAATTGAAAA    718
dim132          AGCGAGTAAAAATCTGCCTGTAAATTTAAGAGCTTATTATCATCTTTTGGCAATTGAAAA    719
11168-O         AGCGAGTAAAAATCTGCCTGTAAATTTAAGAGCTTATTATCATCTTTTGGCAATTGAAAA    719
11168-GS        AGCGAGTAAAAATCTGCCTGTAAATTTAAGAGCTTATTATCATCTTTTGGCAATTGAAAA    718
                ************************************************************
81-176          TTATTTAAAAACTGCAGGAATTGAATTTAGCAGAGATTTTGAACTCACTCCACAAGGGGT    779
33-1            TTATTTAAAAACTGCAGGAATTGAATTTAGCAGAGATTTTGAACTCACTCCACAAGGGGT    779
12-1/9          TTATTTAAAAACTGCAGGAATTGAATTTAGCAGAGATTTTGAACTCACTCCACAAGGGGT    779
dim118/129      TTATTTAAAAACTGCAGGAATTGAATTTAGCAGAGATTTTGAACTCACTCCACAAGGGGT    779
dim120          TTATTTAAAAACTGCAGGAATTGAATTTAGCAGAGATTTTGAACTCACTCCACAAGGGGT    780
dim122/133      TTATTTAAAAACTGCAGGAATTGAATTTAGCAGAGATTTTGAACTCACTCCACAAGGGGT    778
```

```
dim132          TTATTTAAAAACTGCAGGAATTGAATTTAGCAGAGATTTTGAACTCACTCCACAAGGGGT     779
11168-O         TTATTTAAAAACTGCAGGAATTGAATTTAGCAGAGATTTCGAACTCACTCCACAAGGAGT     779
11168-GS        TTATTTAAAAACTGCAGGAATTGAATTTAGCAGAGATTTCGAACTCACTCCACAAGGAGT     778
                ***************************************  ***************** **

81-176          TGATAAAGCCATAAATAAAGAGCTTGAAGTAAAGCTTTTTAATGATAGAATTTTACTTTC     839
33-1            TGATAAAGCCATAAATAAAGAGCTTGAAGTAAAGCTTTTTAATGATAGAATTTTACTTTC     839
12-1/9          TGATAAAGCCATAAATAAAGAGCTTGAAGTAAAGCTTTTTAATGATAGAATTTTACTTTC     839
dim118/129      TGATAAAGCCATAAATAAAGAGCTTGAAGTAAAGCTTTTTAATGATAGAATTTTACTTTC     839
dim120          TGATAAAGCCATAAATAAAGAGCTTGAAGTAAAGCTTTTTAATGATAGAATTTTACTTTC     840
dim122/133      TGATAAAGCCATAAATAAAGAGCTTGAAGTAAAGCTTTTTAATGATAGAATTTTACTTTC     838
dim132          TGATAAAGCCATAAATAAAGAGCTTGAAGTAAAGCTTTTTAATGATAGAATTTTACTTTC     839
11168-O         TGATAAAGCCATAAATAAAGAGCTTGAAGTAAAACTTTTTGATGATAGAATTTTACTTTC     839
11168-GS        TGATAAAGCCATAAATAAAGAGCTTGAAGTAAAACTTTTTGATGATAGAATTTTACTTTC     838
                ********************************* ****** *******************

81-176          TTTAAAAAAATCCAAGAAAGGTTATCAACTATGTGCCTTTTCCGGTAAATAAAGAGTTAAA     899
33-1            TTTAAAAAAATCCAAGAAAGGTTATCAACTATGTGCCTTTTCCGGTAAATAAAGAGTTAAA     899
12-1/9          TTTAAAAAAATCCAAGAAAGGTTATCAACTATGTGCCTTTTCCGGTAAATAAAGAGTTAAA     899
dim118/129      TTTAAAAAAATCCAAGAAAGGTTATCAACTATGTGCCTTTTCCGGTAAATAAAGAGTTAAA     899
dim120          TTTAAAAAAATCCAAGAAAGGTTATCAACTATGTGCCTTTTCCGGTAAATAAAGAGTTAAA     900
dim122/133      TTTAAAAAAATCCAAGAAAGGTTATCAACTATGTGCCTTTTCCGGTAAATAAAGAGTTAAA     898
dim132          TTTAAAAAAATCCAAGAAAGGTTATCAACTATGTGCCTTTTCCGGTAAATAAAGAGTTAAA     899
11168-O         TTTAAAAAAATCCAAGAAAGGTTATCAACTATGTGCCTTTTCCGGTAAATAAAGAGTTAAA     899
11168-GS        TTTAAAAAAATCCAAGAAAGGTTATCAACTATGTGCCTTTTCCGGTAAATAAAGAGTTAAA     898
                ************************************************************

81-176          TTATAACACAAGCAATGAACTTACCGCTGTTATCGCAGAAGGAAATTCCTTTTATATCCA     959
33-1            TTATAACACAAGCAATGAACTTACCGCTGTTATCGCAGAAGGAAATTCCTTTTATATCCA     959
12-1/9          TTATAACACAAGCAATGAACTTACCGCTGTTATCGCAGAAGGAAATTCCTTTTATATCCA     959
dim118/129      TTATAACACAAGCAATGAACTTACCGCTGTTATCGCAGAAGGAAATTCCTTTTATATCCA     959
dim120          TTATAACACAAGCAATGAACTTACCGCTGTTATCGCAGAAGGAAATTCCTTTTATATCCA     960
dim122/133      TTATAACACAAGCAATGAACTTACCGCTGTTATCGCAGAAGGAAATTCCTTTTATATCCA     958
dim132          TTATAACACAAGCAATGAACTTACCGCTGTTATCGCAGAAGGAAATTCCTTTTATATCCA     959
11168-O         TTATAACACAAGCAATGAACTTACGGCTGTTATCGCAGAAGGAAATTCTTTTTATATCCA     959
11168-GS        TTATAACACAAGCAATGAACTTACGGCTGTTATCGCAGAAGGAAATTCTTTTTATATCCA     958
                ************************ *********************** ***********

81-176          GTATGGCAATCGTTTTCAAACAAGATTATATCCTGAGTATTTAGAATTTAGCGATGCTTT     1019
33-1            GTATGGCAATCGTTTTCAAACAAGATTATATCCTGAGTATTTAGAATTTAGCGATGCTTT     1019
12-1/9          GTATGGCAATCGTTTTCAAACAAGATTATATCCTGAGTATTTAGAATTTAGCGATGCTTT     1019
dim118/129      GTATGGCAATCGTTTTCAAACAAGATTATATCCTGAGTATTTAGAATTTAGCGATGCTTT     1019
dim120          GTATGGCAATCGTTTTCAAACAAGATTATATCCTGAGTATTTAGAATTTAGCGATGCTTT     1020
dim122/133      GTATGGCAATCGTTTTCAAACAAGATTATATCCTGAGTATTTAGAATTTAGCGATGCTTT     1018
dim132          GTATGGCAATCGTTTTCAAACAAGATTATATCCTGAGTATTTAGAATTTAGCGATGCTTT     1019
11168-O         ATATGGCAATCGTTTTCAAACAAGATTATATCCTGAGTATTTAGAATTTAGCGATGCTTT     1019
11168-GS        ATATGGCAATCGTTTTCAAACAAGATTATATCCTGAGTATTTAGAATTTAGCGATGCTTT     1018
                ************************************************************

81-176          TAATGAAGTAACTTTTCAAGTTGATGGCAATGAAACCACCGTTCCTTTTGGAACCA-AAG     1078
33-1            TAATGAAGTAACTTTTCAAGTTGATGGCAATGAAACCACCGTTCCTTTTGGAACCA-AAG     1078
12-1/9          TAATGAAGTAACTTTTCAAGTTGATGGCAATGAAACCACCGTTCCTTTTGGAACCA-AAG     1078
dim118/129      TAATGAAGTAACTTTTCAAGTTGATGGCAATGAAACCACCGTTCCTTTTGGAACCA-AAG     1078
dim120          TAATGAAGTAACTTTTCAAGTTGATGGCAATGAAACCACCGTTCCTTTTGGAACCA-AAG     1079
dim122/133      TAATGAAGTAACTTTTCAAGTTGATGGCAATGAAACCACCGTTCCTTTTGGAACCA-AAG     1077
dim132          TAATGAAGTAACTTTTCAAGTTGATGGCAATGAAACCACCGTTCCTTTTGGAACCAAAAG     1079
11168-O         TAATGAAGTAACTTTTCAAGTTGATGGCAATGAAACCACAGTTCCTTTTGGAACCA-AGG     1078
11168-GS        TAATGAAGTAACTTTTCAAGTTGATGGCAATGAAACCACAGTTCCTTTTGGAACCA-AGG     1077
                *************************************** **************** * *

81-176          TAAAAGTCAAAGAGAATTTTCTTATTCCAAAGATAGCTAATGTGCGCGTAAATATCATAG     1138
33-1            TAAAAGTCAAAGAGAATTTTCTTATTCCAAAGATAGCTAATGTGCGCGTAAATATCATAG     1138
12-1/9          TAAAAGTCAAAGAGAATTTTCTTATTCCAAAGATAGCTAATGTGCGCGTAAATATCATAG     1138
dim118/129      TAAAAGTCAAAGAGAATTTTCTTATTCCAAAGATAGCTAATGTGCGCGTAAATATCATAG     1138
dim120          TAAAAGTCAAAGAGAATTTTCTTATTCCAAAGATAGCTAATGTGCGCGTAAATATCATAG     1139
dim122/133      TAAAAGTCAAAGAGAATTTTCTTATTCCAAAGATAGCTAATGTGCGCGTAAATATCATAG     1137
dim132          TAAAAGTCAAAGAGAATTTTCTTATTCCAAAGATAGCTAATGTGCGCGTAAATATCATAG     1139
11168-O         TAAAAGTAAAAGAGAATTTTCTTATTCCAAAGATAGCTAATGTGCGCGTAAATATCATAG     1138
11168-GS        TAAAAGTAAAAGAGAATTTTCTTATTCCAAAGATAGCTAATGTGCGCGTAAATATCATAG     1137
                ******* ****************************************************
```

```
81-176          GCTTTGATCATGGAAAAGATGAAAGTGGTATTTTAGTTCATAAAAAAAAATATGCAAACAC     1198
33-1            GCTTTGATCATGGAAAAGATGAAAGTGGTATTTTAGTTCATAAAAAAAAATATGCAAACAC     1198
12-1/9          GCTTTGATCATGGAAAAGATGAAAGTGGTATTTTAGTTCATAAAAAAAAATATGCAAACAC     1198
dim118/129      GCTTTGATCATGGAAAAGATGAAAGTGGTATTTTAGTTCATAAA-AAAATATGCAAACAC      1197
dim120          GCTTTGATCATGGAAAAGATGAAAGTGGTATTTTAGTTCATAAAAAAAAATATGCAAACAC     1199
dim122/133      GCTTTGATCATGGAAAAGATGAAAGTGGTATTTTAGTTCATAAAAAAAAATATGCAAACAC     1197
dim132          GCTTTGATCATGGAAAAGATGAAAGTGGTATTTTAGTTCATAAAAAAAAATATGCAAACAC     1199
11168-O         GCTTTGATCACTCAAAAGATGAAAGTGGTATTTTAGTTCATAAAAAAAAATATGCAAACAC     1198
11168-GS        GCTTTGATCACTCAAAAGATGAAAGTGGTATTTTAGTTCATAAAAAAAAATATGCAAACAC     1197
                **********   ******************************* ***************

81-176          AATACTCTTTAGACATGGCAGGTAAAATTTATAGAGTGGAATTTTATGAGTTAAGAGGAG      1258
33-1            AATACTTTTTAGACATGGCAGGTAAAATTTATAGAGTGGAATTTTATGAGTTAAGAGGAG      1258
12-1/9          AATACTCTTTAGACATGGCAGGTAAAATTTATAGAGTGGAATTTTATGAGTTAAGAGGAG      1258
dim118/129      AATACTCTTTAGACATGGCAGGTAAAATTTATAGAGTGGAATTTTATGAGTTAAGAGGAG      1257
dim120          AATACTCTTTAGACATGGCAGGTAAAATTTATAGAGTGGAATTTTATGAGTTAAGAGGAG      1259
dim122/133      AATACTCTTTAGACATGGCAGGTAAAATTTATAGAGTGGAATTTTATGAGTTAAGAGGAG      1257
dim132          AATACTCTTTAGACATGGCAGGTAAAATTTATAGAGTGGAATTTTATGAGTTAAGAGGAG      1259
11168-O         AATACTCTTTAGACATGGCAGGTAAAATTTATAGAGCGGAATTTTATGAGTTAAGAGGTG      1258
11168-GS        AATACTCTTTAGACATGGCAGGTAAAATTTATAGAGCGGAATTTTATGAGTTAAGAGGTG      1257
                ****** ***************************** ********************* *

81-176          CGAATTTGCAACAACTTTTAGAGGCAAATACCAATAGCAAGTTGATCAAAAATGCTAAAA      1318
33-1            CGAATTTGCAACAACTTTTAGAGGCAAATACCAATAGCAAGTTGATCAAAAATGCTAAAA      1318
12-1/9          CGAATTTGCAACAACTTTTAGAGGCAAATACCAATAGCAAGTTGATCAAAAATGCTAAAA      1318
dim118/129      CGAATTTGCAACAACTTTTAGAGGCAAATACCAATAGCAAGTTGATCAAAAATGCTAAAA      1317
dim120          CGAATTTGCAACAACTTTTAGAGGCAAATACCAATAGCAAGTTGATCAAAAATGCTAAAA      1319
dim122/133      CGAATTTGCAACAACTTTTAGAGGCAAATACCAATAGCAAGTTGATCAAAAATGCTAAAA      1317
dim132          CGAATTTGCAACAACTTTTAGAGGCAAATACCAATAGCAAGTTGATCAAAAATGCTAAAA      1319
11168-O         CAAATTTGCAACAACTTTTAGAGGCAAATATCAATAGCAAGTTGATCAAAAACGCTAAAA      1318
11168-GS        CAAATTTGCAACAACTTTTAGAGGCAAATATCAATAGCAAGTTGATCAAAAACGCTAAAA      1317
                * **************************** ********************* *******

81-176          ATTTGGATTTAAACACTCTTAAAATGGCAAGATCAAAAGATAAATTCTTAGGTTCTATTT      1378
33-1            ATTTGGATTTAAACACTCTTAAAATGGCAAGATCAAAAGATAAATTCTTAGGTTCTATTT      1378
12-1/9          ATTTGGATTTAAACACTCTTAAAATGGCAAGATCAAAAGATAAATTCTTAGGTTCTATTT      1378
dim118/129      ATTTGGATTTAAACACTCTTAAAATGGCAAGATCAAAAGATAAATTCTTAGGTTCTATTT      1377
dim120          ATTTGGATTTAAACACTCTTAAAATGGCAAGATCAAAAGATAAATTCTTAGGTTCTATTT      1379
dim122/133      ATTTGGATTTAAACACTCTTAAAATGGCAAGATCAAAAGATAAATTCTTAGGTTCTATTT      1377
dim132          ATTTGGATTTAAACACTCTTAAAATGGCAAGATCAAAAGATAAATTCTTAGGTTCTATTT      1379
11168-O         ATTTGGATTTAAATACTCTTAAAATGGCAAGATCAAAAGATAAATTCTTAGGTTCTATTT      1378
11168-GS        ATTTGGATTTAAATACTCTTAAAATGGCAAGATCAAAAGATAAATTCTTAGGTTCTATTT      1377
                ************* **********************************************

81-176          TAGTGGAATTTGAATGA        1395
33-1            TAGTGGAATTTGAATGA        1395
12-1/9          TAGTGGAATTTGAATGA        1395
dim118/129      TAGTGGAATTTGAATGA        1394
dim120          TAGTGGAATTTGAATGA        1396
dim122/133      TAGTGGAATTTGAATGA        1394
dim132          TAGTGGAATTTGAATGA        1396
11168-O         TAGTGGAATTTGAATGA        1395
11168-GS        TAGTGGAATTTGAATGA        1394
                *****************
```

B

```
81-176        MKIFLTILFFITSIFALDLDFSVGENGKSLDDNNTVLIFGGIQGDEPGGFHAASLLLSDY 60
33-1          MKIFLTILFFITSIFALDLDFSVGENGKSLDDNNTVLIFGGIQGDEPGGFHAASLLLSDY 60
12-1/9        MKIFLTILFFITSIFALDLDFSVGENGKSLDDNNTVLIFGGIQGDEPGGFHAASLLLSDY 60
11168-O       MKFFLTILFFITSIFALELDFSVGENGKSLDDNNTVLIFGGIQGDEPGGFHAASLLLSDY 60
              **:**************:******************************************

81-176        NITKGKIIVAPNLAFDSIIKRSRGNNGDLNRKFASISPKDPDYKTVQRIKELILLPEVSM 120
33-1          NITKGKIIVAPNLAFDSIIKRSRGNNGDLNRKFASISPKDPDYKTVQRIKELILLPEVSM 120
12-1/9        NITKGKIIVAPNLAFDSIIKRSRGNNGDLNRKFASISPKDPDYKTVQRIKELILLPEVSM 120
11168-O       NITKGKIIVAPNLAFDSIIKRSRGNNGDLNRKFASISPKDPDYKTVQRIKELILLPEVSM 120
              ************************************************************
```

```
81-176          VINLHDGWGFYKPTYIDAMQNPKRWGNSSVIDTSEINASKYPDLENIATQTVNSVNSSLA 180
33-1            VINLHDGWGFYKPTYIDAMQNPKRWGNSSVIDTSEINASKYPDLENIATQTVNSVNSSLA 180
12-1/9          VINLHDGWGFYKPTYIDAMQNPKRWGNSSVIDTSEINASKYPDLENIATQTVNSFNSSLA 180
11168-O         VINLHDGWGFYKPTYIDAMQNPKRWGNSSVIDTSEINASKYPDLENIATQTVNSVNSSLA 180
                ******************************************************.*****
81-176          DPKHAYHLKNTKTQELGDMEMLKALTYFVISNHKAAFANEASKNLPVNLRAYYHLLAIEN 240
33-1            DPKHAYHLKNTKTQELGDMEMLKALTYFVISNHKAAFANEASKNLPVNLRAYYHLLAIEN 240
12-1/9          DPKHAYHLKNTKTQELGDMEMLKALTYFVISNHKAAFANEASKNLPVNLRAYYHLLAIEN 240
11168-O         DPKHAYHLKNTKTQELGDTEMLKALTYFVISNHKAAFANEASKNLPVNLRAYYHLLAIEN 240
                ****************** *****************************************
81-176          YLKTAGIEFSRDFELTPQGVDKAINKELEVKLFNDRILLSLKNPRKVINYVPFPVNKELN 300
33-1            YLKTAGIEFSRDFELTPQGVDKAINKELEVKLFNDRILLSLKNPRKVINYVPFPVNKELN 300
12-1/9          YLKTAGIEFSRDFELTPQGVDKAINKELEVKLFNDRILLSLKNPRKVINYVPFPVNKELN 300
11168-O         YLKTAGIEFSRDFELTPQGVDKAINKELEVKLFDDRILLSLKNPRKVINYVPFPVNKELN 300
                *********************************:**************************
81-176          YNTSNELTAVIAEGNSFYIQYGNRFQTRLYPEYLEFSDAFNEVTFQVDGNETTVPFGTKV 360
33-1            YNTSNELTAVIAEGNSFYIQYGNRFQTRLYPEYLEFSDAFNEVTFQVDGNETTVPFGTKV 360
12-1/9          YNTSNELTAVIAEGNSFYIQYGNRFQTRLYPEYLEFSDAFNEVTFQVDGNETTVPFGTKV 360
11168-O         YNTSNELTAVIAEGNSFYIQYGNRFQTRLYPEYLEFSDAFNEVTFQVDGNETTVPFGTKV 360
                ************************************************************
81-176          KVKENFLIPKIANVRVNIIGFDHGKDESGILVHKKNMQTQYSLDMAGKIYRVEFYELRGA 420
33-1            KVKENFLIPKIANVRVNIIGFDHGKDESGILVHKKNMQTQYFLDMAGKIYRVEFYELRGA 420
12-1/9          KVKENFLIPKIANVRVNIIGFDHGKDESGILVHKKNMQTQYSLDMAGKIYRVEFYELRGA 420
11168-O         KVKENFLIPKIANVRVNIIGFDHSKDESGILVHKKNMQTQYSLDMAGKIYRAEFYELRGA 420
                ***********************.***************** *********.********
81-176          NLQQLLEANTNSKLIKNAKNLDLNTLKMARSKDKFLGSILVEFE        464
33-1            NLQQLLEANTNSKLIKNAKNLDLNTLKMARSKDKFLGSILVEFE        464
12-1/9          NLQQLLEANTNSKLIKNAKNLDLNTLKMARSKDKFLGSILVEFE        464
11168-O         NLQQLLEANINSKLIKNAKNLDLNTLKMARSKDKFLGSILVEFE        464
                ********* **********************************
```

Figure S1. Clustal O (1.2.1) alignment of the *C. jejuni pgp1* gene and gene product from the helical wild type 81-176 strain, straight 81-176 strains derived from CFW passage, helical 11168-O and straight 11168-GS. A, nucleotide sequence of the *C. jejuni pgp1* gene from the wild type 81-176 strain, straight 81-176 strains passaged on CFW (12-1/9 and 33-1), straight *dim* Tn mutants with the morphology being unlinked to the Tn insertion, 11168-O and 11168-GS. The *C. jejuni* 81-176 *pgp1* sequence was identical to the sequence deposited in GenBank (CP000538.1). **B**, amino acid sequences were determined from the nucleotide sequence in **A** and aligned. Mutations in the *pgp1* gene of the *dim* mutants and 11168-GS resulted in frameshift mutations producing premature stop codons and truncated Pgp1 proteins; therefore, to simplify the alignment, the amino acid sequences of the *dim* mutants were not included in the amino acid alignment. The peptidase-M14-like domain is indicated with blue font. Underlined blue text indicates residues from the predicted Zn binding site and yellow highlighted residues indicate putative active site residues. Nucleotide and amino acid changes between the helical 81-176 and 11168 strains are indicated by green font and are underlined. Nucleotide or amino acid changes in straight strains are highlighted by boxed red font. An asterisk (*) below the sequence indicates positions with conserved residues, a colon (:) indicates residues with strongly similar properties, and a period (.) residues with weakly similar properties.

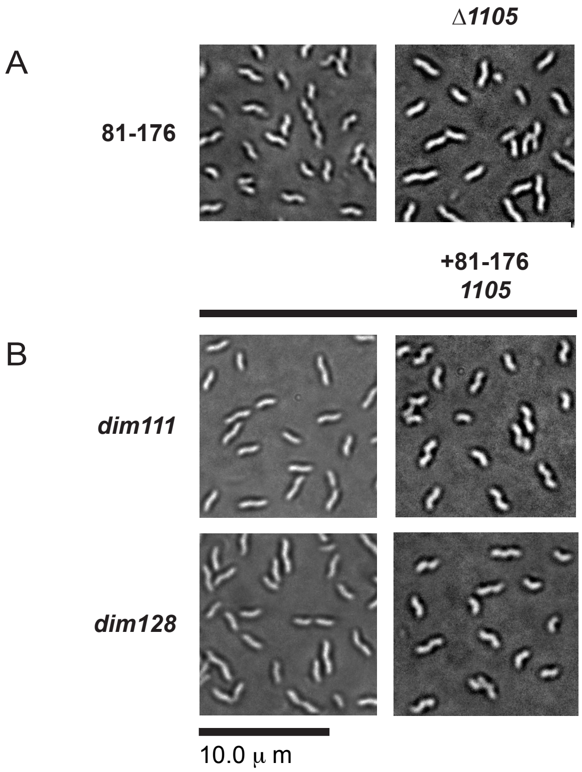

Figure S2. The change in morphology of *C. jejuni* 81-176 Tn mutants *dim111* and *dim128* are not due to the Tn insertion, but are a result of a deletion mutation in *cjj81176_1105.* **A**, DIC microscope images of *C. jejuni* wild type 81-176 and a non-polar insertion mutant in the *cjj81176_1105* gene that exhibits a cell straightening phenotype such as that of *dim111* and *dim128* shown in **B. B**, introduction of a wild type copy of the *1105* gene from plasmid pRRC-*1105* into the rRNA locus of *dim111* and *dim128* restored the morphology defect of those mutants.

A

```
81-176      TTGTTAAAACGACTTGCTTTATTAATTACACTTTCTTCATTGATGTTGCATGCCTCAGAT   60
25-1        TTGTTAAAACGACTTGCTTTATTAATTACACTTTCTTCATTGATGTTGCATGCCTCAGAT   60
            ************************************************************

81-176      CTTGTTAAAATTTATCTTAATCAAGGATTAGATGCTGTTGGTGTAGCGATTGAAAAAGAA   120
25-1        CTTGTTAAAATTTATCTTAATCAAGGATTAGATGCTGTTGGTGTAGCGATTGAAAAAGAA   120
            ************************************************************

81-176      TTAACTCAAAAGGATTTTTGGTTAAGTGAAATAGGAGATAAAAATATTTCACTTGGATAC   180
25-1        TTAACTCAAAAGGATTTTTGGTTAAGTGAAATAGGAGATAAAAATATTTCACTTGGATAC   180
            ************************************************************

81-176      TATGATGATAATGTTGCTATCGTGCTTACAAATAAAACAGATAAAATTCTCCGTGTTTAT   240
25-1        TATGATGATAATGTTGCTATCGTGCTTACAAATAAAACAGATAAAATTCTCCGTGTTTAT   240
            ************************************************************

81-176      TCTTATGAGGACGGAAAAATAAGAAAAGATTTTGAACAAAAAGAAATAATAACTGGATTA   300
25-1        TCTTATGAGGACGGAAAAATAAGAAAAGATTTTGAACAAAAAGAAATAATAACTGGATTA   300
            ************************************************************

81-176      ATGGGCGATAAAAAAATAGAAGGAGATTTGAAAACGCCAGTAGGTTTTTATGAGTTAGGC   360
25-1        ATGGGCGATAAAAAAATAGAAGGAGATTTGAAAACGCCAGTAGGTTTTTATGAGTTAGGC   360
            ************************************************************

81-176      CGTAAGTTTAATCCAGGTGATCCTTATTATGGTCCCTTTGCTTTTGCTACAACTTATCCA   420
25-1        CGTAAGTTTAATCCAGGTGATCCTTATTATTGTCCCTTTGCTTTTGCTACAACTTATCCA   420
            ****************************** *****************************

81-176      AATTTACTTGATAAAGTACAAGGAAAAACAGGTGGTGGTATTTGGATCCATGGCTATCCT   480
25-1        AATTTACTTGATAAAGTACAAGGAAAAACAGGTGGTGGTATTTGGATCCATGGCTATCCT   480
            ************************************************************

81-176      TTAGATGGTTCTAGACTTGATGAATTTAAAACAAGAGGATGTATTGCTTTATTTAATAAT   540
25-1        TTAGATGGTTCTAGACTTGATGAATTTAAAACAAGAGGATGTATTGCTTTATTTAATAAT   540
            ************************************************************

81-176      AATTTGGAGAAATTTGCACAAGTTGTACAAGATAAAAAAGTTTTTGTTATGACAGAAGAA   600
25-1        AATTTGGAGAAATTTGCACAAGTTGTACAAGATAAAAAAGTTTTTGTTATGACAGAAGAA   600
            ************************************************************

81-176      AAAGAAAAAATTAGAGCTAAAAAGGATCAAATAGCTAGTTTATTGGCTGATCTTTTTACA   660
25-1        AAAGAAAAAATTAGAGCTAAAAAGGATCAAATAGCTAGTTTATTGGCTGATCTTTTTACA   660
            ************************************************************

81-176      TGGAAACTAGCTTGGACAAATAGTGACACTAATACCTATTTAAGTTTTTATGATGAGCAA   720
25-1        TGGAAACTAGCTTGGACAAATAGTGACACTAATACCTATTTAAGTTTTTATGATGAGCAA   720
            ************************************************************

81-176      GAATTTAAACGTTTTGATAAAATGAAATTTGAACAGTTTGCTTCCATGAAAAAATCTATT   780
25-1        GAATTTAAACGTTTTGATAAAATGAAATTTGAACAGTTTGCTTCCATGAAAAAATCTATT   780
            ************************************************************

81-176      TTTTCTCGTAAAGAAGATAAAAAGATTAAATTTTCAGATATTAATATCAGCCCTTATCCG   840
25-1        TTTTCTCGTAAAGAAGATAAAAAGATTAAATTTTCAGATATTAATATCAGCCCTTATCCG   840
            ************************************************************

81-176      AATTTAGAAAATGAAACTATGTATAGAATTTCATTTTATGAGGATTATTACACTAAAAAC   900
25-1        AATTTAGAAAATGAAACTATGTATAGAATTTCATTTTATGAGGATTATTACACTAAAAAC   900
            ************************************************************

81-176      TATCAGTTTAGAGGCGATAAAATTTTATACGTTAAGATAGATAGTAAAGGTAAAATGAAA   960
25-1        TATCAGTTTAGAGGCGATAAAATTTTATACGTTAAGATAGATAGTAAAGGTAAAATGAAA   960
            ************************************************************

81-176      ATTTTAGCAGAGCAATAA        978
25-1        ATTTTAGCAGAGCAATAA        978
            ******************
```

B

```
81-76       LLKRLALLITLSSLMLHASDLVKIYLNQGLDAVGVAIEKELTQKDFWLSEIGDKNISLGY    60
25-1        LLKRLALLITLSSLMLHASDLVKIYLNQGLDAVGVAIEKELTQKDFWLSEIGDKNISLGY    60
            ************************************************************
81-76       YDDNVAIVLTNKTDKILRVYSYEDGKIRKDFEQKEIITGLMGDKKIEGDLKTPVGFYELG    120
25-1        YDDNVAIVLTNKTDKILRVYSYEDGKIRKDFEQKEIITGLMGDKKIEGDLKTPVGFYELG    120
            ************************************************************
81-76       RKFNPGDPYYGPFAFATTYPNLLDKVQGKTGGGIWIHGYPLDGSRLDEFKTRGCIALFNN    180
25-1        RKFNPGDPYYCPFAFATTYPNLLDKVQGKTGGGIWIHGYPLDGSRLDEFKTRGCIALFNN    180
            ********** *************************************************
81-76       NLEKFAQVVQDKKVFVMTEEKEKIRAKKDQIASLLADLFTWKLAWTNSDTNTYLSFYDEQ    240
25-1        NLEKFAQVVQDKKVFVMTEEKEKIRAKKDQIASLLADLFTWKLAWTNSDTNTYLSFYDEQ    240
            ************************************************************
81-76       EFKRFDKMKFEQFASMKKSIFSRKEDKKIKFSDINISPYPNLENETMYRISFYEDYYTKN    300
25-1        EFKRFDKMKFEQFASMKKSIFSRKEDKKIKFSDINISPYPNLENETMYRISFYEDYYTKN    300
            ************************************************************
81-76       YQFRGDKILYVKIDSKGKMKILAEQ 325
25-1        YQFRGDKILYVKIDSKGKMKILAEQ 325
            *************************
```

Figure S3. Clustal O (1.2.1) alignment of the *C. jejuni pgp2* gene and gene product from the wild type 81-176 strain and 81-176-derived strain 25-1. A, nucleotide sequence of the *C. jejuni pgp2* gene from the wild type 81-176 strain and the 25-1 strain selected for its straight phenotype after passage on CFW. The *C. jejuni* 81-176 *pgp2* sequence was identical to the sequence deposited in GenBank (NC_008787.1). **B**, amino acid sequences were determined from the nucleotide sequence in **A** and aligned. The LD-catalytic domain (YkuD ; pfam03734) is indicated with blue font. Nucleotide and amino acid changes are highlighted by boxed red font. An asterisk (*) below the sequence indicates positions with conserved residues.

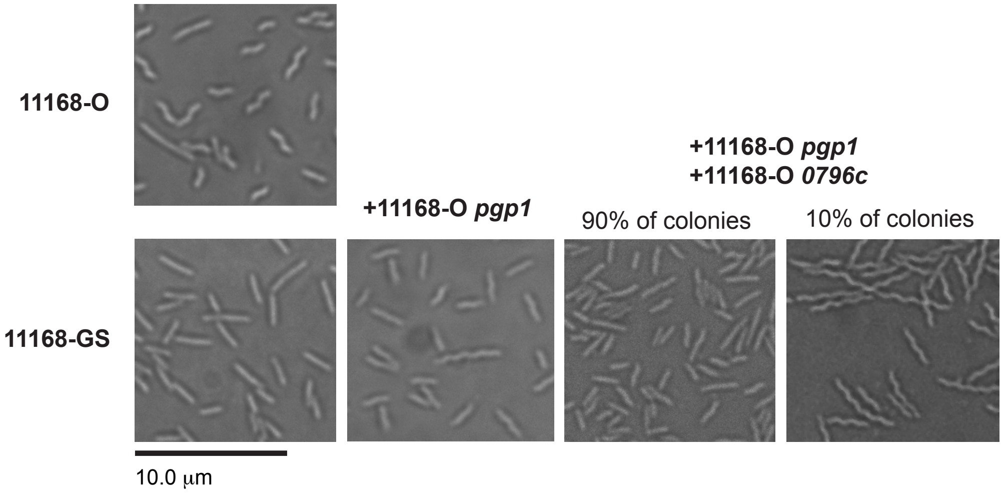

Figure S4. Expression of 11168-O *0796* in a 11168-GS strain expressing 11168-O *pgp1* results in helical filaments in 10% of the colonies. DIC microscope images of *C. jejuni* 11168-O (primarily helical), 11168-GS (rod), 11168-GS expressing 11168-O *pgp1* (very slightly helical) and 11168-GS expressing 11168-O *pgp1* and *cj079*6.

References

Cameron, A. & E.C. Gaynor, (2014) Hygromycin B and apramycin antibiotic resistance cassettes for use in *Campylobacter jejuni*. *PLoS One* **9**: e95084.

Donahue, J.P., D.A. Israel, R.M. Peek, M.J. Blaser & G.G. Miller, (2000) Overcoming the restriction barrier to plasmid transformation of *Helicobacter pylori*. *Mol Microbiol* **37**: 1066-1074.

Frirdich, E., J. Biboy, C. Adams, J. Lee, J. Ellermeier, L.D. Gielda, V.J. Dirita, S.E. Girardin, W. Vollmer & E.C. Gaynor, (2012) Peptidoglycan-modifying enzyme Pgp1 is required for helical cell shape and pathogenicity traits in *Campylobacter jejuni*. *PLoS Pathog* **8**: e1002602.

Frirdich, E., J. Vermeulen, J. Biboy, F. Soares, M.E. Taveirne, J.G. Johnson, V.J. DiRita, S.E. Girardin, W. Vollmer & E.C. Gaynor, (2014) Peptidoglycan LD-carboxypeptidase Pgp2 influences *Campylobacter jejuni* helical cell shape and pathogenic properties and provides the substrate for the DL-carboxypeptidase Pgp1. *J Biol Chem* **289**: 8007-8018.

Gaynor, E.C., S. Cawthraw, G. Manning, J.K. MacKichan, S. Falkow & D.G. Newell, (2004) The genome-sequenced variant of *Campylobacter jejuni* NCTC 11168 and the original clonal clinical isolate differ markedly in colonization, gene expression, and virulence-associated phenotypes. *J Bacteriol* **186**: 503-517.

Glauner, B., J.V. Holtje & U. Schwarz, (1988) The composition of the murein of *Escherichia coli*. *J Biol Chem* **263**: 10088-10095.

Karlyshev, A.V. & B.W. Wren, (2005) Development and application of an insertional system for gene delivery and expression in *Campylobacter jejuni. Appl Environ Microbiol* **71**: 4004-4013.

Korlath, J.A., M.T. Osterholm, L.A. Judy, J.C. Forfang & R.A. Robinson, (1985) A point-source outbreak of campylobacteriosis associated with consumption of raw milk. *J Infect Dis* **152**: 592-596.

Menard, R., P.J. Sansonetti & C. Parsot, (1993) Nonpolar mutagenesis of the ipa genes defines IpaB, IpaC, and IpaD as effectors of *Shigella flexneri* entry into epithelial cells. *J Bacteriol* **175**: 5899-5906.

Parkhill, J., B.W. Wren, K. Mungall, J.M. Ketley, C. Churcher, D. Basham, T. Chillingworth, R.M. Davies, T. Feltwell, S. Holroyd, K. Jagels, A.V. Karlyshev, S. Moule, M.J. Pallen, C.W. Penn, M.A. Quail, M.A. Rajandream, K.M. Rutherford, A.H. van Vliet, S. Whitehead & B.G. Barrell, (2000) The genome sequence of the food-borne pathogen *Campylobacter jejuni* reveals hypervariable sequences. *Nature* **403**: 665-668.

Revez, J., T. Schott, M. Rossi & M.L. Hanninen, (2012) Complete genome sequence of a variant of *Campylobacter jejuni* NCTC 11168. *J Bacteriol* **194**: 6298-6299.

Skirrow, M.B., (1977) *Campylobacter enteritis*: a "new" disease. *Br Med J* **2**: 9-11.
